# A specific serum lipid signature characterizes patients with glycogen storage disease type Ia

**DOI:** 10.1016/j.jlr.2024.100651

**Published:** 2024-09-19

**Authors:** Alessandro Rossi, Margherita Ruoppolo, Roberta Fedele, Francesca Pirozzi, Carmen Rosano, Renata Auricchio, Daniela Melis, Pietro Strisciuglio, Maaike H. Oosterveer, Terry G.J. Derks, Giancarlo Parenti, Marianna Caterino

**Affiliations:** 1 Section of Pediatrics, Department of Translational Medicine, University of Naples “Federico II”, Naples, Italy; 2 Section of Metabolic Diseases, Beatrix Children’s Hospital, University Medical Centre Groningen, University of Groningen, Groningen, The Netherlands; 3 Department of Molecular Medicine and Medical Biotechnology, University of Naples “Federico II”, Naples, Italy; 4 CEINGE Biotecnologie Avanzate s.c.ar.l., Naples, Italy; 5 Department of Medicine, Surgery and Dentistry "Scuola Medica Salernitana", University of Salerno, Salerno, Italy; 6 Department of Pediatrics and Laboratory Medicine, University Medical Center Groningen, University of Groningen, Groningen, The Netherlands; 7 Telethon Institute of Genetics and Medicine, Pozzuoli, Italy

**Keywords:** hyperlipidemia, serum lipidome, ceramides, bile acids, lysophosphatidylcholine

## Abstract

Glycogen storage disease type Ia (GSDIa) is a rare, inherited glucose-6-phosphatase-α (G6Pase-α) deficiency-induced carbohydrate metabolism disorder. Although hyperlipidemia is a hallmark of GSDI, the extent of lipid metabolism disruption remains incompletely understood. Lipidomic analysis was performed to characterize the serum lipidome in patients with GSDIa, by including age- and sex-matched healthy controls and age-matched hypercholesterolemic controls. Metabolic control and dietary information biochemical markers were obtained from patients with GSDIa. Patients with GSDIa showed higher total serum lysophosphatidylcholine (Fold Change, (FC) 2.2, *P* < 0.0001), acyl-acyl-phosphatidylcholine (FC 2.1, *P* < 0.0001), and ceramide (FC 2.4, *P* < 0.0001) levels and bile acid (FC 0.7, *P* < 0.001), acylcarnitines (FC 0.7, *P* < 0.001), and cholesterol esters (FC 1.0, *P* < 0.001) than those of healthy controls, and higher di- (FC 1.1, *P* < 0.0001; FC 0.9, *P* < 0.01) and triacylglycerol (FC 6.3, *P* < 0.0001; FC 3.9, *P* < 0.01) levels than those of healthy controls and hypercholesterolemic subjects. Both total cholesterol and triglyceride values correlated with Cer (d16:1/22:0), Cer (d18:1/20:0), Cer (d18:1/20:0(OH)), Cer (d18:1/22:0), Cer (d18:1/23:0), Cer (d18:1/24:1), Cer (d18:2/22:0), Cer (d18:2/24:1). Total cholesterol also correlated with Cer (d18:1/24:0), Cer (d18:2/20:0), HexCer (d16:1/22:0), HexCer (d18:1/18:0), and Hex2Cer (d18:1/20:0). Triglyceridelevels correlated with Cer (d18:0/24:1). Alanine aminotransferase values correlated with Cer (d18:0/22:0), insulin with Cer (d18:1/22:1) and Cer (d18:1/24:1), and HDL with hexosylceramide (HexCer) (d18:2/23:0). These results expand on the currently known involvement of lipid metabolism in GSDIa. Circulating Cer may allow for refined dietary assessment compared with traditional biomarkers. Because specific lipid species are relatively easy to assess, they represent potential novel biomarkers of GSDIa.

Glycogen storage disease type Ia (GSDIa, OMIM #232200) is an inherited carbohydrate metabolism disorder caused by pathogenic variants in the *G6PC1* gene that cause glucose-6-phosphatase-α (G6Pase-α) deficiency. G6Pase-α is an endoplasmic reticulum (ER)-protein catalyzing the hydrolysis of glucose 6-phosphate (G6P) to glucose and phosphate. G6P requires G6P transporter to enter the ER. Thus, G6Pase-α deficiency results in G6P accumulation in the ER lumen and cytosol. Diet is the cornerstone of the treatment of patients with GSDIa. The prescribed dietary regimens comprise a combination of frequent daytime feedings of uncooked cornstarch (UCCS) and/or extended-release cornstarch (Glycosade®) and/or continuous nocturnal gastric drip-feeding (CNGDF) (1, 2). Despite strict (dietary) management, patients may develop metabolic decompensation (3) and several long-term complications, including liver neoplasms (4), renal diseases (5), and endocrine disorders (6, 7).

As elevated total cholesterol (TC) and triglycerides (TGs) are hallmarks of GSDIa, having been reported in more than 90% and 50% of patients with GSDIa, respectively (8), research has investigated the possible cause(s) of hyperlipidemia in GSDIa, proposing several mechanisms, including increased de novo lipogenesis (9), impaired VLDL clearance (10, 11), and hepatic fatty acid oxidation inhibition (12). However, the exact nature and consequences of hyperlipidemia remain unclear. Currently, TC and TG are major biomarkers for patient monitoring (13). This is particularly relevant because hyperlipidemia can be resistant to current treatments in some patients with GSDIa (8, 14). In addition, elevated circulating TG levels during childhood are associated with an increased risk of liver adenomas in patients with GSDIa (15) and can concur with the progression of renal disease (5).

To date, research on lipid metabolism in GSDIa has mainly focused on traditional components (i.e. TC and TG) (16). In the dietary management treatment of patients with GSDIa, dietary regimens and macronutrient intake are highly personalized to meet individual needs (2). Consequently, any study assessing circulating lipid levels is likely to be affected by the observed heterogeneity among patients with GSDIa (13).

To establish the role of TC and TG in the biosynthesis of complex lipids, a more extensive assessment of the lipid status in GSDIa is warranted. Incidentally, the accumulation of lipid metabolism products in patients with GSDIa has been suggested (6). Elevated plasma 1-deoxysphingolipid concentrations (17), as well as increased long-chain fatty acids, have also been reported (18), suggesting a greater disruption of lipid metabolism in GSDIa. By detecting hundreds of different lipid species, lipidomics provides a quantitative catalog of all lipids present in a specific biological sample. Therefore, it appears to be a promising tool to potentially clarify the extent of lipid metabolism changes and identify potential novel GSDIa biomarkers (19). Proof-of-concept studies using omics technologies have provided novel insights into the pathophysiology of several diseases, including nonalcoholic steatohepatitis (20) and diabetes (21). The current study aimed to perform serum lipidome analysis to further characterize GSDIa-related hyperlipidemia.

## Materials and Methods

### Study participants

This study was conducted in accordance with the principles of the Declaration of Helsinki. All studies were performed after informed consent was obtained from adult participants or the parents of infants. For patients (P11 and P12) followed up at the University Medical Center Groningen, the local Medical Ethical Committee indicated that the Medical Research Involving Human Subjects Act was not applicable and official study approval by the Medical Ethical Committee (METc) was not required according to protocol n. METc 2019/119. The experimental protocols were approved by the METc at the University Medical Center Groningen in accordance with the relevant guidelines and regulations included in the n. METc 2019/119 protocol.

For patients (P1-P10) followed up at the University of Naples Federico II, the study protocol was approved by the Ethics Committee of the University of Naples Federico II n. 77/21. The experimental protocols were approved by the Ethics Committee at the University of Naples Federico II in accordance with the relevant guidelines and regulations included in n. 77/21 protocol.

The participants were recruited over a 12-month period. The study enrolled 12 patients with GSDIa (8 males and 4 females, median age 16.5 ± 9 years, age range 5.0–34.0 years), 13 age- and sex-matched healthy controls (HCs) (9 males and 4 females, median age 17.0 ± 9.1 years, age range 5.0–31.5 years), and 7 participants with hypercholesterolemia due to inherited conditions other than GSDIa (“hypercholesterolemic controls (4 males and 3 females, median age 12.0 ± 2.7 years, age range 9.0–17.0 years). There was no significant difference between the three groups. GSDIa diagnosis was based on the mutation analysis of the *G6PC1* gene. The diagnosis of hyperlipidemia due to inherited conditions other than GSDIa was based on mutation analysis of the *LDLR* (*c.661G > A, c.682G > A, c.1646G > A, c.352G > A, IVS15-3C > A* pathogenic variants) or *APOB* (*c.9175C > T* pathogenic variant) genes. Among the patients, one was aged <6 years, five were aged 10–16 years, and six were aged >17 years. Among the healthy controls (HCs), one was aged <6 years, five were aged 10–16 years, and seven were aged >17 years. Among the hypercholesterolemic controls, six were aged 9–14 years, and one was aged 17 years.

All patients were on dietary treatment. The HCs did not receive any dietary or pharmacological treatments. Hypercholesterolemic controls were treated with atorvastatin and/or ezetimibe, followed by a low-lipid diet. For patients with GSDIa, the following clinical data were recorded: genotype, BMI, traditional biochemical markers (glucose, lactate, uric acid, and TC), HDL cholesterol, LDL cholesterol, TGs, aspartate aminotransferase (AST), alanine aminotransferase (ALT), serum insulin levels, estimated glomerular filtration rate, and microalbuminuria). In 9 out of 13 HCs and in 7 out of 7 hypercholesterolemic controls, the following clinical data were recorded: traditional biochemical markers (glucose, uric acid, and TC), TGs, AST, and ALT. In addition, HDL and LDL cholesterol levels were measured in the hypercholesterolemic controls.

### Dietary assessment

Each patient with GSDIa received frequent feeding along with UCCS and/or CNGDF. Dietary regimens varied among the patients according to their families’ requests and attitudes. Data on feeding frequency, dietary regimen, UCCS intake, and the proportion of macronutrients contributing to the total energy intake (TEI) were collected. The following data on macronutrient intake were recorded: grams UCCS (or Glycosade)/day, grams/kg UCCS (or Glycosade)/day, grams carbohydrates (CHO)/day, grams CHO/kg/day, kcal from CHO/kg/day, grams protein/day, grams protein/kg/day, grams fat/day, grams fat/kg/day, Kcal from fat tot/day, kcal/day [i.e, total energy intake total day, %CHO to TEI, % protein to TEI, % fat to TEI. Information on any drugs and/or supplementations was also recorded.

### Serum collection and preparation

Blood samples were collected in a preprandial state according to each patient’s fasting tolerance (3–6 h). To minimize bias due to short fasting times, healthy and hypercholesterolemic controls were asked to schedule their blood sampling after the same fasting time as their age- and sex-matched GSDIa counterparts. Moreover, 4 out of 12 patients underwent additional blood sampling at two times distant from each other for 6 days to assess intrapatient variability. Blood was centrifuged (10 min, 2000*g* at 4°C), and serum was aliquoted into separate polypropylene tubes that were immediately stored at −80°C.

### Targeted lipidomic analysis

The serum lipidome was characterized using targeted metabolomics. According to Mxp Quant 500 protocols (Biocrates Life Sciences, Innsbruck, Austria), the lipidomic mass spectrometry-based platform was set to measure the concentration of 549 lipids belonging to six classes: (i) acylcarnitines (ACs) (n = 40); (ii) free fatty acids (n = 12); (iii) sterol lipids, including cholesterol esters (CEs) (n = 22) and bile acids (BAs) (n = 14); (iv); glycerolipids, including diacylglycerols (DGs) (n = 44) and TGs (n = 242); (v) glycerophospholipid molecules, including lysophosphatidylcholine (LysoPC) (n = 14), acyl-acyl-phosphatidylcholine (PCaa) (n = 38), and alkyl-acyl-phosphatidylcholine (PCae) (n = 38); vi) sphingolipids, including sphingomyelins (SMs) (n = 15), ceramides (Cer) (n = 28), dihydroceramides and hydroxyceramides (dihydroCer and hydroxyCer) (n = 8), and hexosylceramides, dihexosylceramides, trihexosylceramides (HexCer, Hex2Cer, Hex3Cer) (n = 34). Quantification was carried out using internal standards and a calibration curve (Cal 1 to Cal 7). Three human plasma samples spiked with different concentrations of reference analytes (QC1-3) were analyzed as quality control, according to the manufacturer’s protocol. Briefly, 10 μl of samples (serum sample, quality controls, zero samples, or calibrators) were processed (22, 23, 24, 25). Subsequently, 10 μl of samples were added onto filter inserts and dried for 30 min under a nitrogen stream. The serum samples were derivatized for 20 min in 5% phenyl isothiocyanate in ethanol/water/pyridine (ratio 1/1/1, v/v/v), and the mixture was subsequently dried for 60 min under a nitrogen stream. Lipids were extracted with 300 μl methanol containing 5 mM ammonium acetate by shaking for 30 min, and then eluted by centrifugation for 5 min at room temperature and 450 rpm.

The mass spectrometry-based lipidome platform combined a flow injection analysis (FIA) and a LC method. For the LC part, 150 μl of each extract was diluted with an equal volume of HPLC grade water, while for the FIA part, 10 μl of each extract was diluted with 490 μl of FIA solvent (provided by Biocrates). After dilution, LC-MS/MS measurements were performed to target and quantify BA and free fatty acids, while FIA-MS/MS measurements were performed to target and quantify the remaining lipids using a multiple reaction monitoring-based method. Lipidomics analysis was performed using a Triple Quad™ 5500+ System QTrap-Ready (AB Sciex) coupled to an Agilent 1260 Infinity II HPLC. The injection volume for liquid chromatography analysis was 5 μl. LC was performed on a C18 column (Zorbax eclipse XDB 3 × 100 mm, 3.5 μm) using acetonitrile and water with 0.2% formic acid as mobile phase. For FIA analysis, the injection volume was 20 μl using the FIA kits running solvent mobile at an initial flow rate of 0.03 ml/min for 1.6 min, followed by 0.20 ml/min for 1.6 min and 0.02 ml/min for 0.20 min. The autosampler was cooled at 10°C. The ion source was operated in positive ion mode with the following parameters: spray voltage 55 kV, temperature 450°C, GS1 20, GS2 40, CUR 30, CAD 8. The ion source was operated in negative ion mode with the following parameters: spray voltage 55 kV, temperature 450°C, GS1 20, GS2 40, CUR 30, CAD 8. Data were acquired using Analyst software (version 1.7 Ab Sciex) and lipid concentrations were calculated directly in MetIDQ™ Oxygen 2976 (Biocrates Life Sciences Innsbruck, Austria).

### Feature selection and data analysis

The serum lipidome dataset was filtered to remove features with more than 50% missing values. The remaining missing values were replaced with one-fifth of the minimum positive value of each variable in the dataset. The data were then log_10_-transformed and scaled according to the Pareto scaling method. Chemometric and cluster analyzes were performed using MetaboAnalyst 4.0 (http://www.metaboanalyst.ca) (26). Differential lipid abundance in GSDIa patients versus controls was assessed using a volcano plot analysis based on log10 of abundance-difference, using a false discovery rate of 1% to correct for multiple comparisons and a two-stage step-up (Benjamini, Krieger, and Yekutieli) adjustment method.

Statistical analysis of the serum GSDIa dataset was performed according to the univariate method using GraphPad Prism 9.0 (GraphPad Software, Boston, MA, USA) (27, 28). The single molecule concentrations are reported as the mean ± standard error of the mean (mean ± SEM). Total lipid content is expressed as the sum of the lipid quantity within a specific lipid class (mean ± SEM) in patients with GSDIa and healthy and hypercholesterolemic controls. Significant differences were established by performing: (i) one-way ANOVA and Tukey’s multiple comparison test with Holm–Sidak’s multiple comparison test for normally distributed datasets or nonparametric Kruskal–Wallis test with Dunn’s multiple comparison test in nonnormally distributed datasets; normal distribution was verified according to D’Agostino and Pearson’s tests; (ii) two-way ANOVA and Tukey’s multiple comparison test, with individual variances computed for each comparison. Analysis of regression was performed according to computed nonparametric Spearman’s rank correlations (two-tailed and 95% confidence interval) to assess the relationships between serum lipid abundances and GSDIa biochemical markers and/or dietary intake data. A P_two-tailed < 0.01% and 95% agreement interval was considered statistically significant. Bland–Altman analyzes were performed to determine the interday variability of the serum abundances of a selected group of lipids, considering two different blood sampling times. Data were further analyzed using the coefficient of variation to examine intersubject and intrasubject variability.

## Results

### Patients with GSDIa display altered serum lipid content

The GSDIa serum lipidomic profile was determined by analyzing a cohort of 12 patients with GSDIa and 13 age and sex-matched HCs. Genetic and clinical features, including dietary regimens, drugs, and/or supplements, and the biochemical parameters of patients with GSDIa are presented in Tables 1 and 2, respectively. Detailed information on macronutrient intake was available for 10 patients with GSDIa (Supplemental Table S1).

**Table 1:** Genetic and clinical features of patients with GSDIa

Subject	Age (years)	Gender	Protein variation	BMI (kg/m^2^)	Dietary regimen	Drugs/Supplements	Notes
1	5	M	p.Arg83Cys	21.0	Frequent feedings	Vitamin D	Intellectual disability
			p.Arg83Cys		UCCS (3.8 g/kg/day)		
					CNGDF		
2	10	M	p.Arg83Cys	19.7	Frequent feedings	Allopurinol	-
			p.Arg83Cys		CNGDF	Vitamin D	
						ω3 fatty acids	
3	17	M	p.Trp63Arg	21.4	Frequent feedings	-	Previous GH deficiency
			p.Arg83Cys		Glycosade (3.1 g/kg/day)		(rhGH withdrawn 6 months before study enrollment)
4	25.5	F	p.Trp63Arg	24.4	Frequent feedings	Allopurinol	Proteinuria
			p.Arg83Cys		Glycosade (3.2 g/kg/day)	Ramipril	
						Fenofibrate	
5	24	F	p.Arg83Cys	22.5	Frequent feedings	ω3 fatty acids	-
			p.Arg83Cys		UCCS (3.9 g/kg/day)		
6	34	M	p.Arg83Cys	26.7	Frequent feedings	Allopurinol	-
			p.Arg83Cys		Glycosade (4.5 g/kg/day)	Carbamazepine	
7	11	F	p.Arg83Cys	22.2	Frequent feedings	Allopurinol	Glomerular hyperfiltration
			p.Arg83Cys		UCCS (4.8 g/kg/day)	Ramipril	
						Vitamin D	
						ω3 fatty acids	
8	28.5	M	p.Arg83Cys	29.6	Frequent feedings	Oxybutynin	Urinary incontinence
			p.Arg83Cys		UCCS (1.5 g/kg/day)		
					CNGDF		
9	24	F	p.Arg83Cys	26.3	Frequent feedings	Allopurinol	Glomerular hyperfiltration
			p.Arg83Cys		UCCS (4.3 g/kg/day)	Ramipril	
						Vitamin D	
						ω3 fatty acids	
10	12	M	p.Arg83Cys	19.7	Frequent feedings	Vitamin D	-
			p.Arg83Cys		UCCS (5.2 g/kg/day)		
11	10	M	p.Gly270Val	21.7	Frequent feedings	Vitamin D	-
			p.Gly270Val		UCCS (6.3 g/kg/day)		
12	16	M	p.Gly270Val	20.2	Frequent feedings	Allopurinol	Hepatocarcinoma
			p.Gly270Val		Glycosade (2.1 g/kg/day)	Gemfibrozil	
					CNGDF		

UCCS, uncooked cornstarch; continuous nocturnal gastric drip-feeding; GH, growth hormone.

**Table 2:** Biochemical markers in patients with GSDIa

Subject	Glucose (mmol/l)	Lactate (mmol/l)	Uric acid (μmol/l)	Total cholesterol (mmol/l)	HDL (mmol/l)	LDL (mmol/l)	Triglycerides (mmol/l)	AST (U/l)	ALT (U/l)	Insulin (μU/ml)	eGFR Ml/min/1,73 m^2^	Microalbuminuri (mg/dl)
Patients with GSDIA												
P1	4.7	5.8	392.6	6.9	1.0	5.1	5.1	78	94	4.7	120	47.0
P2	9.1	6.7	499.6	10.6	0.9	2.1	26.1	121	136	62.3	155	12.7
P3	4.0	5.3	386.6	5.8	1.7	3.4	3.1	132	79	11.2	181	8.0
P4	7.9	-	294.8	6.5	1.1	4.9	6.1	18	17	13.5	185	4.0
P5	5.2	4.0	166.5	4.8	1.3	3.0	2.0	20	17	15.7	131	15.0
P6	5.5	11.0	237.9	4.8	0.3	3.3	2.6	18	19	32.0	154	16.3
P7	3.9	5.7	404.5	5.6	1.3	1.1	10.3	87	86	11.8	176	0.0
P8	5.0	3.8	303.9	-	-	-	-	23	44	40.8	-	0.0
P9	4.6	6.2	279.6	6.9	0.6	-	5.5	53	64	27.3	170	66.0
P10	4.5	3.5	410.4	5.0	1.2	3.4	2.6	40	56	10.1	154	-
P11	4.9	5.3	422.3	6.0	-	-	5.4	43	34	-	245	-
P12	5.0	4.1	422.3	8.1	0.7	4.8	13.6	43	20	-	-	-

AST, aspartate aminotransferase; ALT, alanine aminotransferase; eGFR, estimated glomerular filtration rate; -, not available; SE, standard error.

Targeted lipidomics analysis was performed on serum samples from patients with GSDIa and HCs to detect and quantify 549 lipids. A detailed list of the identified and quantified lipids, including their names, abbreviations, and group classifications, is shown in Supplemental Table S2, and the analytical lipid concentrations of each patient and control are summarized in Supplemental Table S3.

**Fig. 1:**
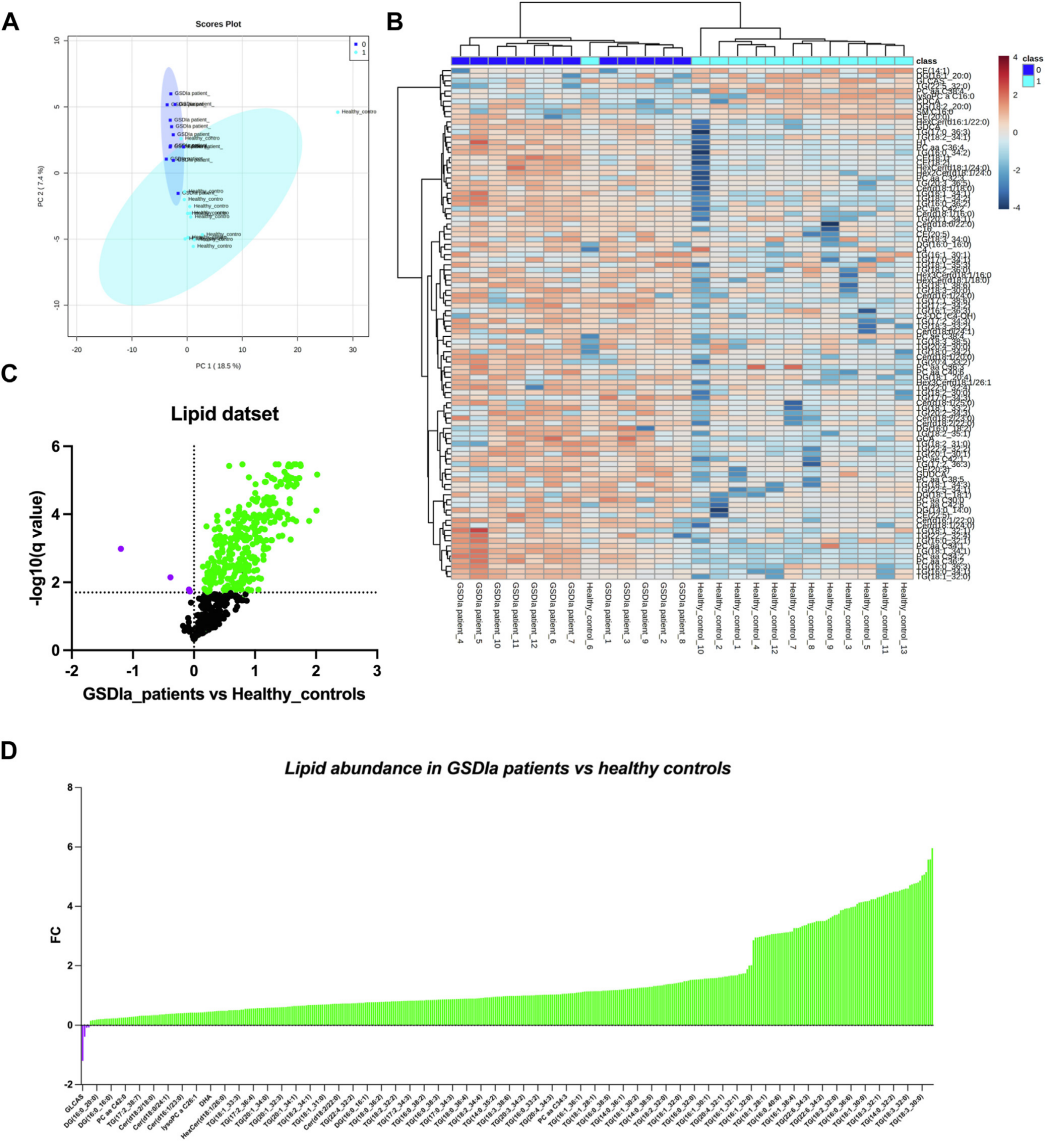
Glycogen storage disease type Ia (GSDIa) lipidomic signature model. A: A principal component analysis (PCA) was performed using the normalized lipid dataset. Patients with GSDIa and healthy controls are represented by different colors (*dark blue*: patients with GSDIa; *light blue*: healthy controls). Phenotype conditions showed clear separation according to two principal components (PCs), explaining 25.9% of the total variance (PC1 18.5% and PC2 7.4%). B: Hierarchical cluster analysis and heatmap visualization of the lipid dataset top 100 (*y*-axis) ranked by a *t* test (*P* < 0.05). Serum lipid abundance was log (10) transformed and Pareto scaled. The color code of the heatmap represents the relative metabolite abundance: *red* and *blue* represent increased and decreased levels of each lipid in patients with GSDIa versus healthy controls, respectively. C: Volcano plot analysis of differential lipid abundance in patients with GSDIa versus healthy controls. The relative abundance of each lipid was plotted against its statistical significance, reported as Difference (log 10 abundance) and -log10 (q-value), respectively. *Purple* and *green dots* refer to significantly decreased and increased lipids. *Black dots* refer to all the lipids identified in the dataset whose relative abundance is not significantly changed between patients with GSDIa vs. healthy controls. D: The plot shows the differential lipid abundance in patients with GSDIa versus healthy controls. *Purple* and *green bars* refer to significantly decreased and increased lipids, respectively.

Multivariate and univariate statistical analyzes were used to identify the most significant alterations in serum lipid levels between patients with GSDIa and controls. Specifically, the lipid dataset was processed using principal component analysis to evaluate the differences in rate and variance between the groups (patients with GSDIa and HCs). The principal component analysis score plot revealed a clear separation between the analyzed data groups according to the PC1 and PC2 variances of 18.0% and 7.2%, respectively. Outliers were found in the HC group but not in the GSDIa group (Fig. 1A). To further highlight the alterations in the serum lipidome of patients with GSDIa, a heatmap was used to visualize the differentially abundant lipids in patients with GSDIa and HCs (Fig. 1B), and the lipid concentrations were ranked by the *t* test (*P* < 0.05). The dendrogram structure using Euclidean distance showed correct sample group clustering, excluding one HC (control_6). Heatmap visualization of the top 100 identified and quantified lipids exhibited a distinct pattern of lipid abundance between the GSDIa and control groups. Differential lipid patterns may be associated with pathological phenotypes.

To assess the general lipid accumulation related to GSDIa, a binary comparison was performed using a volcano plot (Fig. 1C), and all differentially represented lipids are shown in Figure 1D and Supplemental Table S4. A total of 319 lipids were found to be more abundant in the serum of patients with GSDIa than in the serum of HCs in a volcano plot analysis. Of these, TGs are the most abundant (Fig. 1D). The least abundant lipid in the serum of patients with GSDIa was found to be glycolithocholic acid sulfate (GLCAS). To further elucidate the additional lipidome components that accounted for the observed accumulation, specific lipid classes were investigated.

### Several serum lipid classes increase in patients with GSDIa

To define altered lipid content as specifically being disease-related, an additional control group was investigated, including individuals with hypercholesterolemia due to inherited conditions other than GSDIa (hypercholesterolemic controls). Tables 3 and 4 lists the comparison between the traditional serum biochemical profile of 9 patients with GSDIa and 9 age- and sex-matched HCs and between 6 patients with GSDIa and 7 age-matched hypercholesterolemic controls, respectively. Significant differences in serum levels of uric acid (*P* < 0.05), TC (*P* ≤ 0.001), TG, AST, and ALT (*P* ≤ 0.01) were observed between patients with GSDIa and HCs (Table 3). Significant differences in serum levels of uric acid (*P* ≤ 0.00001), TG and ALT (*P* < 0.05), AST (*P* ≤ 0.01) were observed between patients with GSDIa and hypercholesterolemic controls (Table 4).

**Table 3:** Biochemical markers in 9/13 Healthy controls (HC) and patients with GSDIa age and sex matched

Subject	Glucose (mmol/l)	Uric acid (μmol/l)	Total cholesterol (mmol/l)	Triglycerides (mmol/l)	AST (U/l)	ALT (U/l)
HC						
HC1	4.1	279.6	3.8	0.6	16	12
HC2	4.8	434.2	3.2	0.6	30	52
HC3	4.1	297.4	4	0.6	15	13
HC7	5	261.7	3.5	0.7	16	20
HC9	4.4	261.7	4.1	1.1	17	15
HC10	4.3	339	4	0.5	20	12
HC11	3.3	255.8	3.4	0.6	25	14
HC12	3.8	303.4	4.3	0.5	18	8
HC13	4.1	356.9	4.4	0.7	18	6
Mean	4.2	310.0	3.9	0.7	19.4	16.9
SE	0.2	22.0	0.2	0.1	1.9	5.2
Patients with GSDIa age and sex matched						
P1	4.7	392.6	6.9	5.1	78	94
P2	9.1	499.6	10.6	26.1	121	136
P3	4.0	386.6	5.8	3.1	132	79
P6	5.5	237.9	4.8	2.6	18	19
P7	3.9	404.5	5.6	10.3	87	86
P8	5.0	303.9	-	-	23	44
P10	4.5	410.4	5.0	2.6	40	56
P11	4.9	422.3	6.0	5.4	43	34
P12	5.0	422.3	8.1	13.6	43	20
Mean	5.2	386.7	6.6	8.6	65.0	63.1
SE	0.5	22.4	0.6	2.5	12.4	11.6

Significance (*P* value)						
	Glucose (mmol/l)	Uric acid (μmol/l)	Total cholesterol (mmol/l)	Triglycerides (mmol/l)	AST (U/l)	ALT (U/l)
GSDIa versus HC	0.095	0.028	0.001	0.010	0.005	0.004

AST, aspartate aminotransferase; ALT, alanine aminotransferase; -, not available; SE, standard error.

**Table 4:** Biochemical markers in 7/7 hypercholesterolemic controls (Hyper_controls) and patients with GSDIa age matched

Subject	Glucose (mmol/l)	Uric Acid (μmol/l)	Total Cholesterol (mmol/l)	HDL (mmol/l)	LDL (mmol/l)	Triglycerides (mmol/l)	AST (U/l)	ALT (U/l)
Hyper_controls								
Hyper_control1	3.7	255.8	8.7	1	7.4	1.2	28	34
Hyper_control2	4.2	255.8	5.2	1.3	3.7	0.6	19	15
Hyper_control3	4.3	226	6.2	1.3	4.8	0.9	27	28
Hyper_control4	3.6	178	5.1	1.3	3.7	0.7	23	19
Hyper_control5	3.7	309.3	6.6	1.1	6	0.7	19	15
Hyper_control6	4.2	249.8	3.5	0.9	2.5	0.8	44	41
Hyper_control7	3.3	237.9	3.1	1.2	1.4	1.9	31	61
Mean	3.9	244.7	5.5	1.2	4.2	1.0	27.3	30.4
SE	0.1	14.9	0.7	0.1	0.8	0.2	3.3	6.3
Patients with GSDIa age matched								
P2	9.1	499.6	10.6	0.9	2.1	26.1	121	136
P3	4.0	386.6	5.8	1.7	3.4	3.1	132	79
P7	3.9	404.5	5.6	1.3	1.1	10.3	87	86
P10	4.5	410.4	5.0	1.2	3.4	2.6	40	56
P11	4.9	422.3	6.0	-	-	5.4	43	34
P12	5.0	422.3	8.1	0.7	4.8	13.6	43	20
Mean	5.2	424.3	6.9	1.2	3.0	10.2	77.7	68.5
SE	0.8	16.0	0.9	0.2	0.6	3.6	17.1	17.0

Significance (*P* value)								
	Glucose (mmol/l)	Uric acid (μmol/l)	Total cholesterol (mmol/l)	HDL (mmol/l)	LDL (mmol/l)	Triglycerides (mmol/l)	AST (U/l)	ALT (U/l)
GSDIa versus Hyper_controls	0.092	000.001	0.248	0.986	0.266	0.019	0.010	0.047

AST, aspartate aminotransferase; ALT, alanine aminotransferase; -, not available; SE, standard error.

**Fig. 2:**
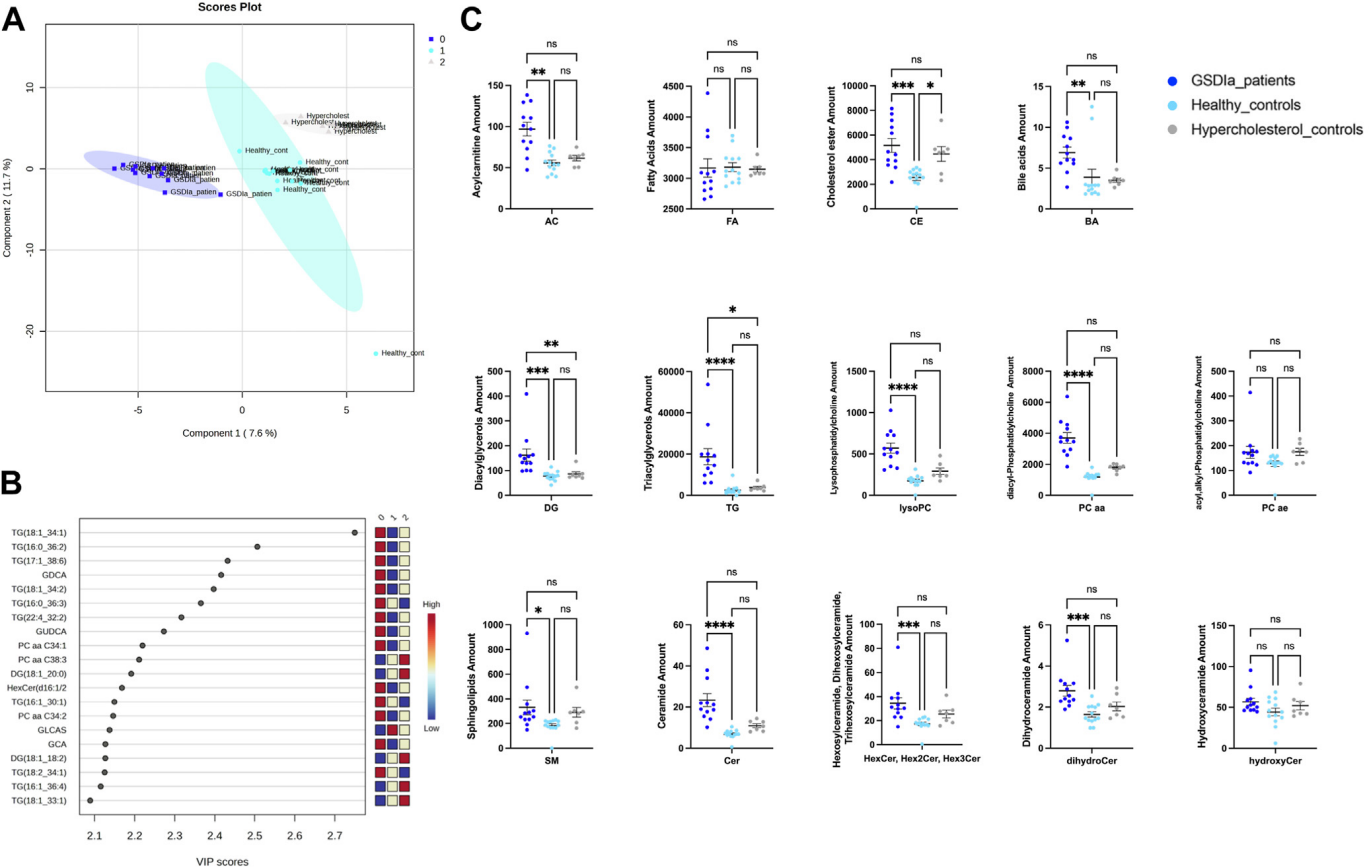
Descriptive discriminant analysis of serum glycogen storage disease type Ia (GSDIa) lipidome. A: Differential segregation (components 1 and 2: 7.2% and 12.5%, respectively) was observed in the serum lipid content of patients with GSDIa and healthy and hypercholesterolemic controls. B: Discriminant features were identified according to the variable importance in projection (VIP) score, and the 20 most important molecules with VIP scores >2.0 were reported. Box color intensity represents the relative abundance for each group. The concentrations of the identified lipids were normalized, log(10) transformed, and Pareto scaled. C: Total lipid content, expressed as the sum of lipid quantity within a specific lipid class (mean ± SEM) in patients with GSDIa (*dark blue*) and healthy (*light blue*) and hypercholesterolemic controls (gray). Ceramides reflect those of Supplemental Table S2 with sphingosine and sphingadiene; hydroxyceramides those with sphingosine and sphinganine.

The serum lipid datasets of patients with GSDIa and healthy and hypercholesterolemic controls were analyzed using supervised partial least squares-discriminant analysis to investigate the most discriminant lipid molecules among the three groups, extracting a combination of variables/lipids that could be considered to have predictive value (Fig. 2A). The variable importance in projection (VIP) measure was used to identify the most discriminant lipids characterizing patients compared with each control group (Fig. 2B) and allowed the identification of a subcluster of 20 lipids that were largely affected by the pathological phenotype according to their VIP score (VIP >2). Specifically, the levels of TG (18:1_34:1), TG (16:0_36:2), TG (17:1_38:6), glycodeoxycholic acid (GDCA), TG (18:1_34:2), TG (16:0_36:6), and TG (22:4_32:2) allowed discrimination between the analyzed groups (VIP> 2.3). The distribution profile of serum lipid classes in the three groups (i.e patients with GSDIa and healthy and hypercholesterolemic controls) was achieved by summing all normalized quantities of identified lipids within a single class, expressed as nmol of each lipid in 1 μl serum (Fig. 2C). As expected, the total CE and TG contents were higher in patients with GSDIa than control groups, reflecting traditional biochemical plasma parameters, as TC and TG levels reported in supplemental Figure S1 (Tables 3 and 4).

**Fig. 3:**
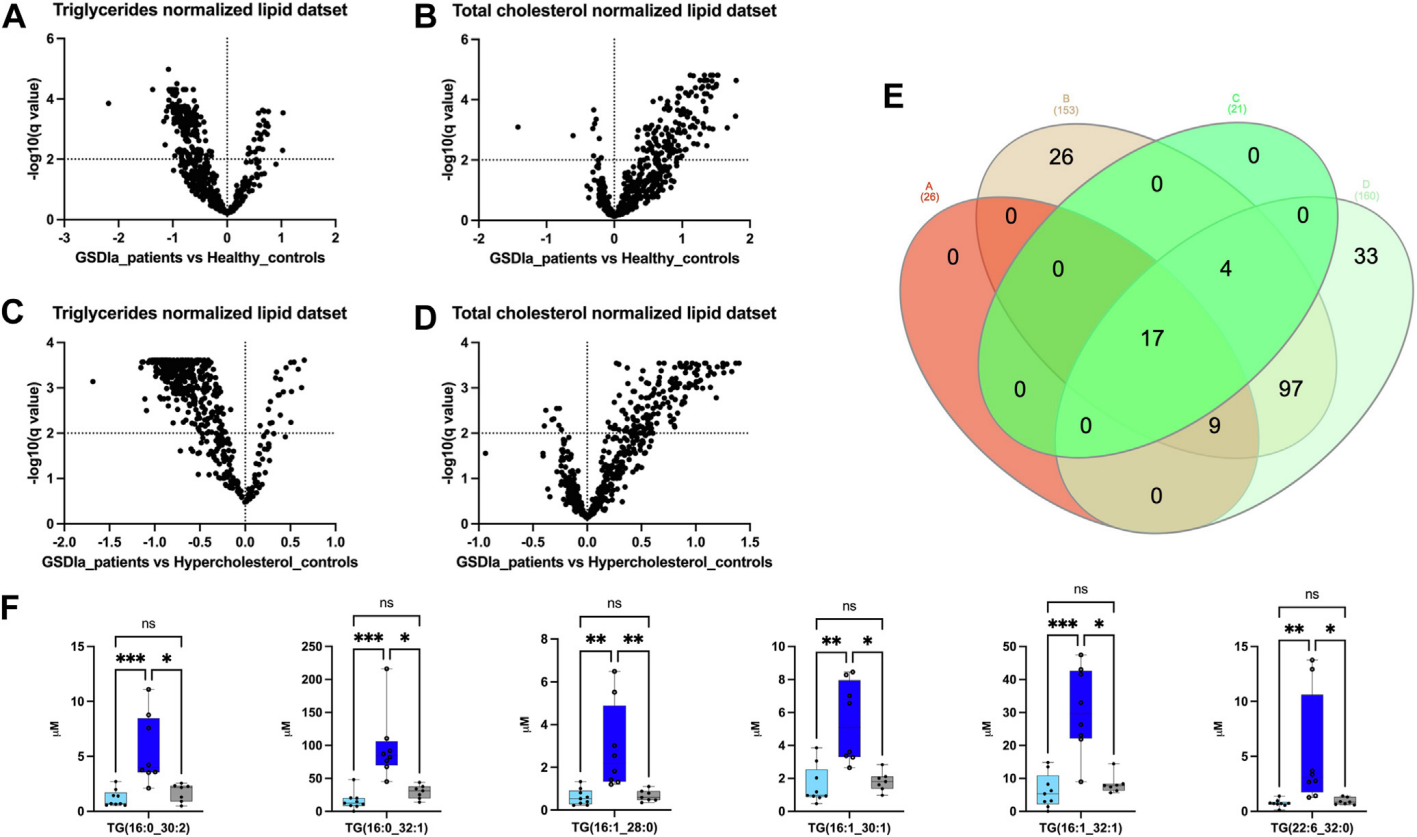
Differential lipidome abundance in patients with GSDIa versus healthy controls and hypercholesterol controls. The datasets were normalized according to plasma level of triglycerides and total cholesterol. The relative abundance of each lipid was plotted against its statistical significance, reported as Difference (log 10 abundance) and -log10 (q-value), respectively. GSDIa versus. healthy controls volcano plot analysis of (A) plasma triglycerides and (B) total cholesterol normalized datasets; GSDIa versus hypercholesterol controls volcano plot analysis of (C) plasma triglycerides and (D) total cholesterol normalized datasets; (E) Euler Venn diagram selection of 17 lipid common species shared by all binary comparisons. F: Box and whisker plots summarizing the concentrations of 6 selected lipids, found to be more significant abundant in patients with GSDIa compared to both control groups. Significant differences were established by performing one-way ANOVA followed by a Holm–Sidak multiple comparison test for normally distributed datasets and Kruskal–Wallis and Dunn’s multiple comparison tests for nonnormally distributed datasets (∗*P* < 0.05, ∗∗*P* < 0.01, ∗∗∗*P* < 0.001 ∗∗∗∗*P* < 0.0001, ns = not significant). Normality was verified according to D’Agostino and Pearson tests. GSDIa, glycogen storage disease type Ia.

Additionally, ACs, DG, BAs, lysoPC, PCaa, SMs, Cer, HexCer, and dihydroceramides (DihydroCer) were highly increased in patients with GSDIa relative to those in HCs. Except for the DG and TG class, no statistical differences were found in the serum lipid concentrations in respect to hypercholesterolemic controls. In addition, to relate the effect of hyperlipidemia to the patients’ lipidomic profiles, the full dataset was normalized to biochemical plasma measurement of TC and TGs, which was available for 8 out of 12 GSDIa patients, 9 out of 13 HCs and for 7 out of 7 hypercholesterolemic controls. The binary comparative analyses of normalized lipid datasets were performed by comparing GSDIa to HCs (Fig. 3A, B) and GSDIa to hypercholesterolemic controls (Fig. 3C, D). The more abundant lipid species that were statistically significant in both normalized lipid datasets in GSDIa patients relative to the two control groups are presented in an Euler Venn diagram (Fig. 3E). In detail, 17 lipid species were found common in all binary comparisons (Supplemental Table S5). Among these, 6 lipids, including TG (16:0_30:2), TG (16:0_32:1), TG (16:1_28:0), TG (16:1_30:1), TG (16:1_32:1), TG (22:6_32:0) were found to be more significantly abundant in patients with GSDIa compared to both control groups. Graphical plots showing the abundances of the aforementioned lipids are shown in Fig. 3F.

### Specific lipid species characterize the serum of patients with GSDIa

To describe the pathological phenotypic signature, univariate statistical approaches were used to select relevant changes in specific lipid molecules within each subclass (Supplemental Fig. S2). The lipidome of hypercholesterolemic controls was used as a negative control.

In detail, the serum levels of lipid molecules, including C2, CE (16:1), DG (14:0_18:1), DG (14:0_18:1), DG (16:0_16:0), DG (16:0_16:1), DG (16:0_18:1), DG (16:0_18:2), DG (16:1_18:1), DG (18:1_18:2), TG (14:0_34:1), TG (14:0_34:2), TG (16:0_32:1), TG (16:0_32:2), TG (16:0_34:1), TG (16:0_34:2), TG (16:0_34:3), TG (16:0_36:2), TG (16:0_36:3), TG (16:0_36:4), TG (16:1_32:0), TG (16:1_32:1), TG (16:1_34:1), TG (16:1_34:2), TG (18:1_30:0), TG (18:1_30:1), TG (18:1_32:0), TG (18:1_32:1), TG (18:1_34:1), TG (18:1_34:2), TG (18:1_36:2, TG (18:1_36:3), TG (18:2_32:0), TG (18:2_32:1), TG (18:2_34:1), TG (18:2_34:2), cholic acid, glycochenodeoxycholic acid (GCDCA), lysoPC a C18:0, lysoPC a C18:2, PC aa C36:3, PC aa C38:3, PC aa C38:6, HexCer (d18:1/23:0), HexCer (d18:1/24:0), Cer (d18:1/20:0(OH)), Cer (d18:1/22:0), Cer (d18:1/23:0), Cer (d18:1/24:0), Cer (d18:1/24:1), and Cer (d18:2/24:0) were altered in GSDIa compared with those in healthy and hypercholesterolemic controls, whereas they did not significantly differ between the two control groups (Supplemental Table S6). Conversely, CE (18:3), DG (18:1_18:1), TG (16:0_32:0), TG (16:0_34:0), TG (16:1_36:2), TG (18:1_32:2), SM C18:0, SM C24:1, lysoPC a C18:1, PC aa C36:1, PC aa C38:5, PC aa C40:6, PC ae C34:1, PC ae C38:6, Hex2Cer (d18:1/16:0), HexCer (d18:1/16:0), HexCer (d18:2/22:0), HexCer (d18:2/24:0), and Cer[d18:1/18:0(OH)] were differentially abundant in patients with GSDIa versus HCs, whereas these species were not significantly different between patients with GSDIa and hypercholesterolemic controls. The significantly different abundant lipids species are summarized in Supplemental Table S6. To assess measurement accuracy, we verified the agreement between two quantitative measurements of samples from the same subject over time. The magnitude of the intraindividual variability in the GSDIa serum lipidome was investigated by evaluating the serum abundance of 20 ACs (Supplemental Table S7) over time. Four patients (P1, P2, P7, and P9) out of twelve provided additional blood samples at two times distant from each other for 6 days. No significant intraindividual variability (*P* > 0.01) over time was observed (Supplemental Fig. S3), considering a 95% agreement interval.

In addition, the intraindividual and interindividual variabilities were estimated using variation coefficients. The intraindividual variabilities were 8.9%, 3.0%, 2.6%, and 1.1% for patients P1, P2, P7, and P9, respectively. The average interindividual variability was 17.8% for the infant patients P1 (5 years), P2 (10 years), and P7 (11 years).

### Diet-dependent serum lipid profiles of patients with GSDIa

A correlation analysis was performed to investigate the contribution of diet to the lipid alterations observed in the serum of patients with GSDIa. A correlation matrix based on Spearman linear regression distance was used to explore the relationships among the 16 dietary parameters. All detected lipid species within each specific class showed significant and specific alterations in lipid profiles when the GSDIa lipidome was compared with that of the two control groups. No significant correlations were observed between altered lipid species and any of the dietary data (Supplemental Fig. S4). To further assess the effect of the dietary regimen on the lipid alterations in the serum of patients with GSDIa, the dataset was analyzed according to patient dietary regimen by comparing patients with GSDIa receiving CNGDF (n = 4) and UCCS/Glycosade only (n = 8). No significant differences were observed in any of the lipid species (Supplemental Fig. S5).

### Age-dependent serum lipid profiles of patients with GSDIa

To evaluate the age-dependence of the lipid alterations in the serum of patients with GSDIa, the dataset was analyzed according to patient age, comparing GSDIa_Children (n = 7, age <18 years, 11.0 ± 4.0 years) and GSDIa_Adult (n = 5, age >18, 25.5 ± 4.2 years) with their corresponding control groups, i.e, HC_Children (n = 7, age <18 years, 11.1 ± 4.0 years) and HC_Adult (n = 6, age >18, 28.5 ± 2.6 years) (Supplemental Figs. S6–S8, and Table S8). Notably, TG (14:0_34:1), TG (16:0_32:2), TG (16:1_32:1), TG (18:1_30:0), TG (18:3_32:0), TG (18:3_32:1), TG (18:3_34:2), TG (20:4_32:1), glycocholic acid, GCDCA, GDCA, SM C24:1, lysoPC a C18:1, lysoPC a C18:2, HexCer (d18:1/16:0), HexCer (d18:1/23:0), and Cer (d18:2/24:0) were specifically increased in the serum of children with GSDIa. Significant differences in serum lipid species were not found when the adult patient group was compared with the adult control group or between children and adult patients. Finally, increased levels of CE (18:1), CE (18:2), DG (16:0_16:0), DG (16:0_18:1), TG (22:6_34:1), and lysoPC (C16:0, PC aa C34:2) were found in GSDIa_Children compared with HC_Children, GSDIa_Adults compared with HC_Adults, and GSDIa_Children compared with GSDIa_Adults. In particular, among these CE (18:2) was found to correlate (*P* < 00,046) with the age parameter in Spearman-based correlation analysis (Supplemental Table S9). CE (20:4), which was only found to be elevated in GSDIa_Adults compared to HC_Adults, was also found to correlate with age (*P* < 00,091) (Supplemental Table S9). SM C16:0, HexCer (d18:1/24:1), TG (16:0_32:1), TG (16:0_34:2), TG (16:1_34:1), TG (18:1_32:1), TG (18:1_34:1), and TG (18:3_34:1) were increased in GSDIa_Children compared with those in HC_Children as well as in GSDIa_Adults compared with those in HC_Adults, whereas they were decreased in GSDIa_Children compared with those in GSDIa_Adults (Supplemental Table S8).

### Clinical parameters correlated with GSDIa altered lipidomic profile

**Fig. 4:**
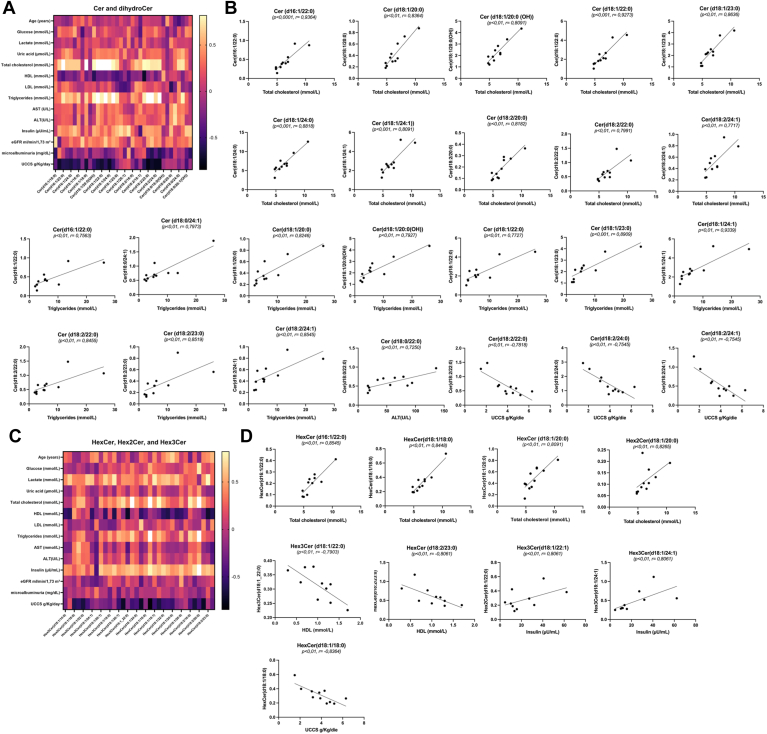
Spearman’s rank correlations of ceramides/dihydroceramides and hexosylceramides in glycogen storage disease type Ia (GSDIa). The matrix correlates GSDIa serum ceramide/dihydroceramide (A) and hexosylceramide (C) abundances with the diagnostic parameters of patients with GSDIa. The negative and positive associations are represented by *dark black* and *light yellow*, respectively. B: Ceramides/dihydroceramides were significantly associated with (i) total cholesterols, (ii) total triacylglycerides, and (iii) ALT (*P* < 0.01; *r* > 0.5), as well as (iv) daily UCCS/Glycosade intake (*P* < 0.01, *r* < −0.5). D: Hexosylceramides were significantly associated with (i) total cholesterols (ii) HDL, and (iii) insulin (*P* < 0.01; *r* > 0.5), as well as (iv) daily UCCS/Glycosade intake (*P* < 0.01; *r* < −0.5). The specific values of Spearman rank correlation coefficient are reported in Supplemental Table S7. ALT, alanine aminotransferase; UCCS, uncooked cornstarch.

The relationship between the biochemical parameters of GSDIa and serum lipidomes was investigated. A correlation matrix based on Spearman’s correlation distance was used to explore the correlations among the 20 clinical parameters and age (Table 2; Supplemental Table S9) and all detected lipid species within each specific class. Significant and specific alterations in lipid profiles were observed when the GSDIa lipidome was compared with those of the two control groups. The analysis revealed significant correlations between Cer, DihydroCer (Fig. 4A, B), and HexCer (Fig. 4C, D) and GSDIa clinical parameters. Specifically, Cer (d16:1/22:0), Cer (d18:1/20:0), Cer [d18:1/20:0(OH)], Cer (d18:1/22:0), Cer (d18:1/23:0), Cer (d18:1/24:1), Cer (d18:2/22:0), Cer (d18:2/24:1) correlated with TC and TG levels; Cer (d18:1/24:0), Cer (d18:2/20:0), HexCer (d16:1/22:0), HexCer (d18:1/18:0), and Hex2Cer (d18:1/20:0) linearly correlated with TC levels. In addition, Cer (d18:0/24:1) correlated with TG, Cer (d18:0/22:0) with ALT, Cer (d18:1/22:1) and Cer (d18:1/24:1) with insulin. A negative correlation was observed between daily UCCS/Glycosade intake and HexCer (d18:1/18:0), Cer (d18:2/22:0), Cer (d18:2/24:0), and Cer (d18:2/24:1) levels, and between HexCer (d18:2/23:0) and HDL levels. Conversely, no correlation was observed between serum TG and TC levels and daily UCCS/Glycosade intake in patients with GSDIa.

## Discussion

Hyperlipidemia is a hallmark of GSDIa. However, the exact nature and potential consequences of hyperlipidemia in patients with GSDIa remain unclear. To extensively characterize GSDIa serum lipid status, lipidomic analysis was performed in patients, as well as in age- and sex-matched healthy and age-matched hypercholesterolemic controls. The collected data showed that patients with GSDIa display a unique lipid profile with increased levels of specific lipid classes, including AC, CE, BA, DG, LysoPC, Pcaa, SM, Cer, HexCer, and DihydroCer. The observed accumulation of these complex lipid molecules may be due to increased de novo synthesis as a consequence of increased availability of substrates such as fatty acids and cholesterol. Changes in metabolic fluxes induced by cytosolic G6P accumulation and subsequent increases in other phospho-monoesters markedly enhance substrate availability for de novo synthesis of fatty acids and cholesterol (9, 29). Detailed insights into lipid class composition are expected to have a profound impact on the management of GSDIa, from which liver adenomas, kidney disease, and insulin resistance/metabolic syndrome can develop (16). This is of particular relevance as current lipid biomarkers do not appear to be sufficiently accurate for stratifying phenotypic heterogeneity (10).

Among the identified lipids, Cer appear to be the key to defining GSDIa in both childhood and adulthood.

Cers are not a single lipid entity; rather, they are a family of signaling lipids that represent the central metabolites of the sphingolipid family and form the lipid backbone to which a diverse array of headgroup structures is conjugated, regulating distinct physiological processes (30). Cers also act as signaling molecules that regulate cellular processes such as ER stress, apoptosis, and insulin sensitivity, which in turn may affect whole-body physiology.

Cer synthesis in mammals is catalyzed by a family of six Cer synthases (CerS1–6), which introduce a variable-length fatty acyl-coenzyme A (CoA) to the amine group of a sphingolipid base. Previous studies have shown that different CerS isoforms exhibit strong preferences for fatty acyl-CoAs with different carbon chain lengths. CerS1 exclusively uses 18 carbon (C18) fatty acids to form Cer (d18:1/18:0); conversely, CerS2 forms d18:1/24:0 and d18:1/24:1 Cer (31). Other studies have linked insulin resistance and hepatic steatosis to excess hepatic Cer in mouse models by not specifically affecting Cer synthesis by blocking total sphingolipid synthesis (32). The present study revealed the altered abundance of Cer class in the GSDIa serum lipidome and uncovered novel clues to the characterization of this class of molecules, which has never been investigated in relation to GSDIa pathology. The significant correlation between GSDIa serum Cer and DihydroCer levels and patient diagnostic parameters, such as TC and TG levels, highlights the importance of Cer in disease pathophysiology.

Preclinical studies in CerS1^−/−^ mice have shown a higher respiratory capacity, as demonstrated by increased levels of respiratory complex proteins in liver and adipose tissue (30), suggesting increased mitochondrial activity. In contrast, the accumulation of Cer, whose synthesis is strictly dependent on palmitoyl availability, indirectly suggests reduced mitochondrial capacity with a slowdown in β-oxidation. Previous studies have indicated that in GSDIa, hepatic fatty acid oxidation inhibition occurs due to increased lipogenic flux, driving malonyl-CoA accumulation (12). Based on the present results, we propose that Cer accumulation may also reflect fatty acid overload in GSDIa (33).

Cer accumulation has also been observed in patients with Cushing disease (34). In addition, hypercortisolism has been reported in patients with GSDIa (7). Since cortisol levels were not assessed in the present study, a possible mechanism linking cortisol and Cer levels remains to be established.

The diet also appears to contribute to circulating Cer levels (35). Although evidence on the specific Cer affected by diet and the direction thereof is conflicting (36), increased circulating Cer levels have been observed in overfed individuals (37). Hence, we hypothesized that dietary (over) treatment contributes to Cer accumulation in patients with GSDIa. Unexpectedly, no direct correlation was found between serum lipid species and any dietary parameters in patients with GSDIa. Conversely, a negative correlation was found between a subgroup of Cer and daily UCCS/Glycosade intake, consistent with previous findings showing a paradoxical decrease in Cer levels following 4-weeks dietary supplementation with SM and Cer (38). Similarly, UCCS/Glycosade may modulate Cer absorption and/or microbial metabolism and, ultimately, circulating Cer levels (36). Since no correlation between daily UCCS/Glycosade intake and TC/TG was observed in the present study, these observations suggest that the circulating Cer profile may allow a more refined assessment of UCCS/Glycosade treatment in patients with GSDIa than current biomarkers. Future studies investigating the relationship between UCCS/Glycosade intake and enterocyte metabolism in patients with GSDIa are warranted.

Hyperlipidemia in GSDIa is linked to the progression of long-term complications and is a major focus of management strategies. To evaluate age-specific patterns in serum lipid composition, we defined, for the first time, an age-matched lipid signature by comparing lipid abundance in groups of patients of different ages, i.e., pediatric and adult patients.

Indeed, the relative abundances of specific lipids (i.e., CE, DG, TG, LysoPC, PCaa, and HexCer classes), which discriminate the disease condition from the healthy control state, were also affected by the age of patients with GSDIa. The discriminant lipid profile, characterizing adult patients with GSDIa relative to infant patients with GSDIa, may also reflect a higher risk of disease complications in aging patients with GSDIa.

Serum BA and lysoPC levels are also of particular interest. Our findings indicate a possible imbalance in lipid transport and absorption, as demonstrated by alterations in serum BA levels. In addition, besides facilitating lipid absorption, BAs act as signaling molecules that modulate glucose and lipid metabolism. Uncontrolled hyperglycemia and insulin resistance perturb BA metabolism (39). As BA species have distinct physicochemical properties that determine their efficacy in promoting fat and cholesterol absorption, as well as their signaling functions, specific BAs affect key metabolic pathways. We found altered serum abundances of CA and GCDCA between patients with GSDIa and controls, while CA, glycocholic acid, GCDCA, and GDCA were identified as age-dependent discriminants in patients. These findings are supported by those of previous animal studies showing that intrahepatic G6P accumulation in GSDIa regulates BA metabolism, specifically by increasing CA synthesis (29). Notably, CA levels and serum chenodeoxycholic acid levels increased in 83% and 100% of patients with GSDIa compared with previously reported reference ranges. These data suggest possible cholestatic liver disease in patients with GSDIa, which is not addressed by the current guidelines, as it may be missed by traditional biomarkers (e.g., bilirubin and γ-glutamyltransferase) (1, 2). Therefore, further studies investigating BA levels in larger populations are warranted.

Finally, an imbalance in circulating lysoPC levels has been proposed as a putative marker of oxidative stress and mitochondrial dysfunction. In the liver, lysophosphatidylcholines upregulate genes involved in cholesterol biosynthesis and downregulate those involved in hepatic fatty acid oxidation (40). Higher lysophosphatidylcholine concentrations disrupt mitochondrial integrity and enhance cytochrome C release by hepatocytes. Previous studies investigating mitochondrial volume in the livers of young patients with GSDIa (41) and tricarboxylic acid cycle intermediates in cellular and animal models have proposed derangements in mitochondrial structure and a decrease in mitochondrial number in hepatic GSDIa (42).

The present study has both strengths and weaknesses. First, the study population included both pediatric and adult patients, allowing us to investigate the effect of age on the relative abundance of lipid species. Second, two control groups (i.e healthy and hypercholesterolemic) were included to assess the effect of hyperlipidemia (irrespective of its origin) on the serum lipidomic profile. However, a relatively small number of patients with GSDIa were enrolled without a second independent population, limiting the statistical power to evaluate differences in lipid signatures between the subgroups analyzed. Although this issue was partially mitigated by the inclusion of two control groups, small sample sizes are a common and acknowledged issue in studies on rare disorders. Therefore, intraindividual and interindividual variability was compared, thereby supporting the reliability of our results. Additionally, the relative contribution of G6Pase-α deficiency per se and diet to the lipid abnormalities observed in the present study remains difficult to elucidate. Dietary treatment to prevent GSDIa-related hypoglycaemia in patients with GSDIa is highly personalized (2). Therefore, any attempt to standardize dietary schemes appears to be poorly feasible and poses major ethical issues in GSDIa. Although all patients with GSDIa enrolled in this study were on a regular diet according to the current guidelines (2), the dietary regimens were highly personalized to meet each individual’s needs and fasting tolerance. Notably, lipids accounted for 8.4–25.5% of TEI in patients with GSDIa and were below 20% of TEI in 70% of patients with GSDIa compared with <30% in a healthy population (https://www.who.int/news-room/fact-sheets/detail/healthy-diet). Furthermore, no correlation was observed between the serum lipid species and dietary macronutrient intake in patients with GSDIa. Collectively, these observations suggest that macronutrient intake did not contribute to the differences in the lipid species observed in the present study. Thus, preclinical studies investigating the lipidome under standardized dietary conditions are warranted. Finally, 8 of the 12 patients with GSDIa carried the homozygous p.Arg83Cys variant in the *G6PC* gene that is predicted to completely abolish G6Pase-α activity (43); therefore, correlation analysis of the serum lipidome and genotype was not possible in the present study.

In summary, the present study demonstrates that characterizing the serum lipidomic profile is feasible in patients with GSDIa. A unique GSDIa serum lipidomic profile, particularly with respect to Cer, BAs, and lysoPC, was identified. The results of this study provide novel insights into the extent of lipid metabolism disruption in patients with GSDIa. As such, they have potential translational applications not only in clinical care but also in clinical trials evaluating novel treatment options. Recent international priority-setting partnerships for liver GSDs have emphasized the need for new methods to monitor metabolic control (44).

Circulating Cers have been proposed as biomarkers for predicting the stratification of metabolic syndrome-associated risks (45, 46), and a Cer score has been developed (47). Circulating Cers have also been used as effective biomarkers of novel lipid-lowering drugs (48, 49, 50). Therefore, the results of our study promote drug repurposing for treating GSDIa-related hyperlipidemia (8, 14).

Overall, our findings pave the way for improved monitoring of patients with GSDIa, potentially allowing for refined (dietary) management and early diagnosis of disease complications. Future cross-sectional and prospective studies assessing lipid biomarkers in (un)treated individuals with GSDIa are warranted.

## Data availability

Data supporting the findings of this study are available in the article and Supplementary Information.

## Supplemental data

This article contains supplemental data.

## Conflict of interest

The authors declare that they have no conflicts of interest with the contents of this article.
