# Supplementary material for: A specific serum lipid signature characterizes patients with glycogen storage disease type Ia
Source: J Lipid Res. 2024 Sep 19;65(10):100651. doi: 10.1016/j.jlr.2024.100651 (PMC11526085; doi:10.1016/j.jlr.2024.100651)

Supplementary Figure S1

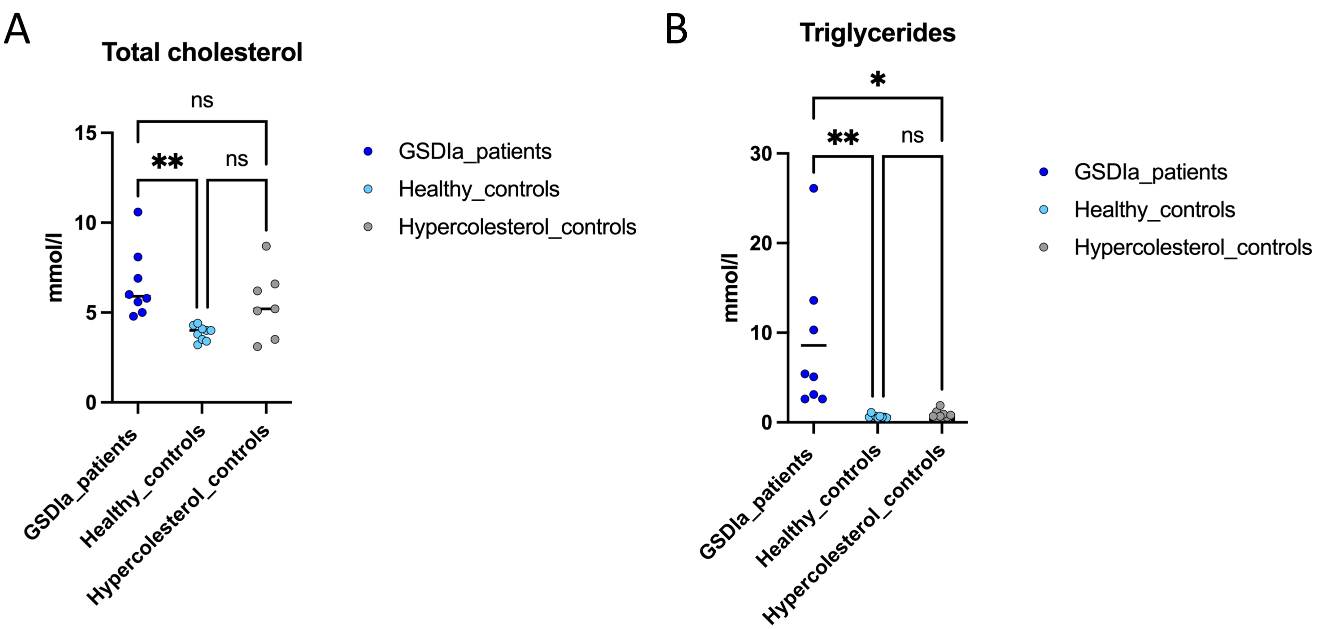

Supplementary Figure S2

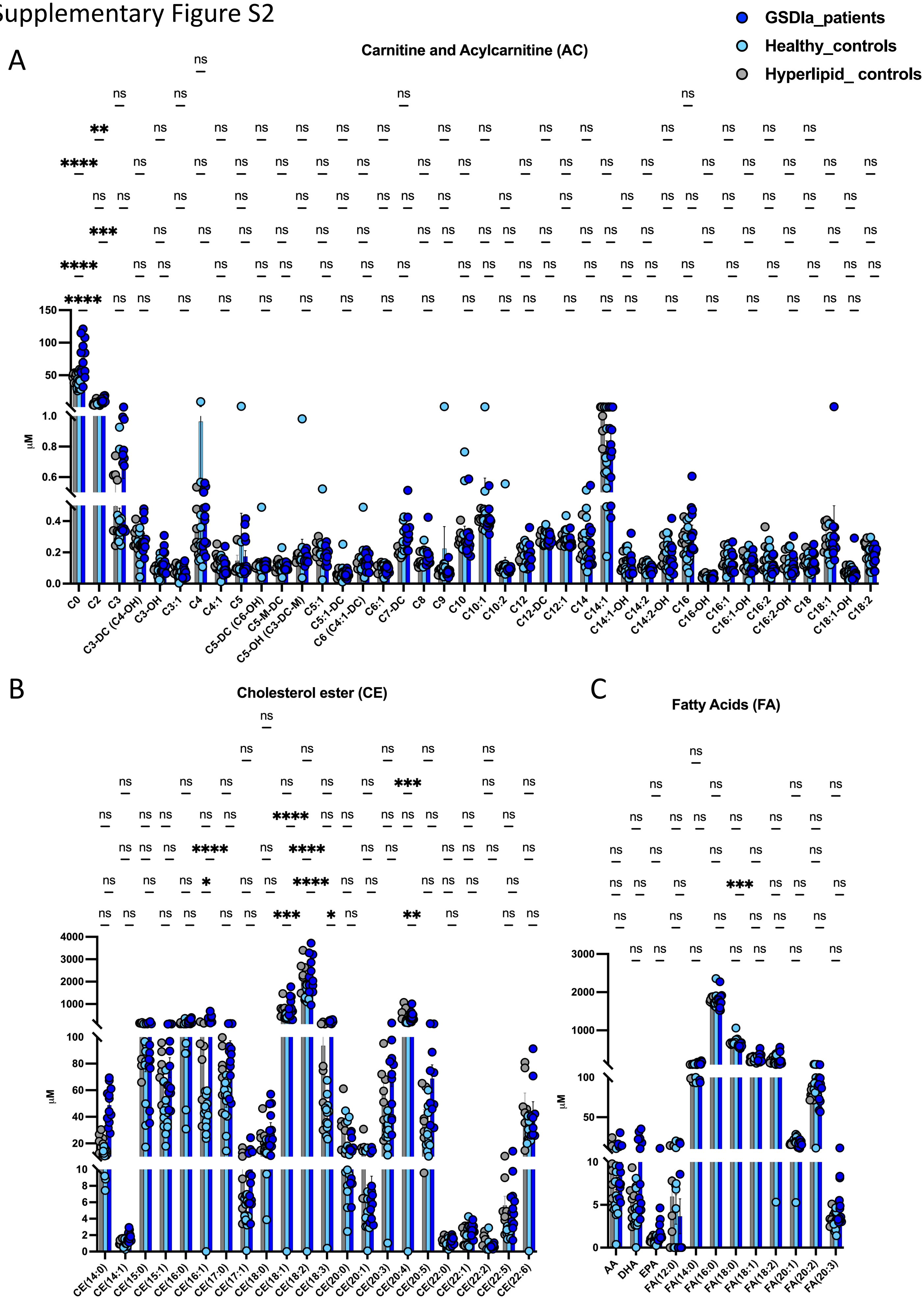

Supplementary Figure S2

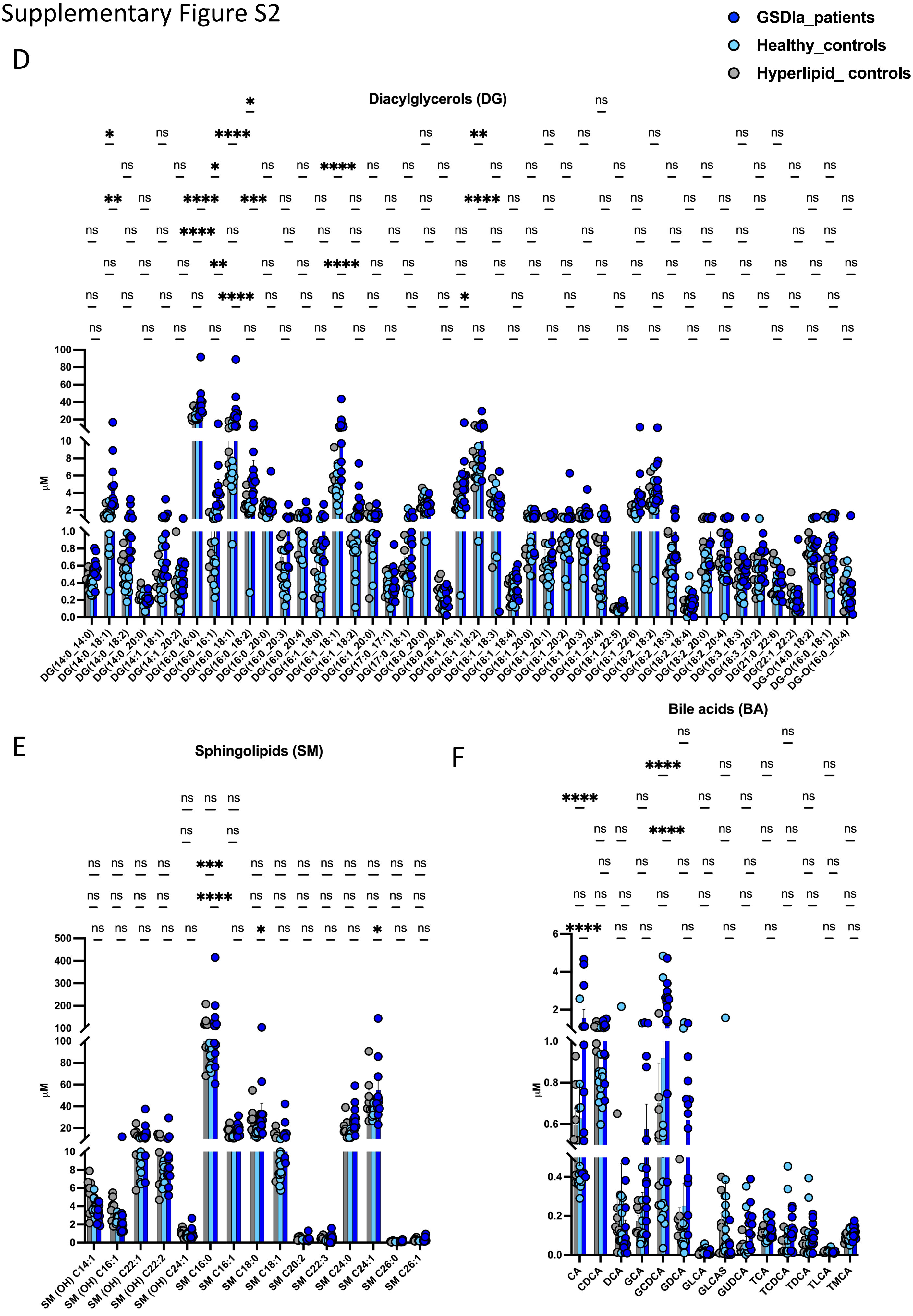

Supplementary Figure S2

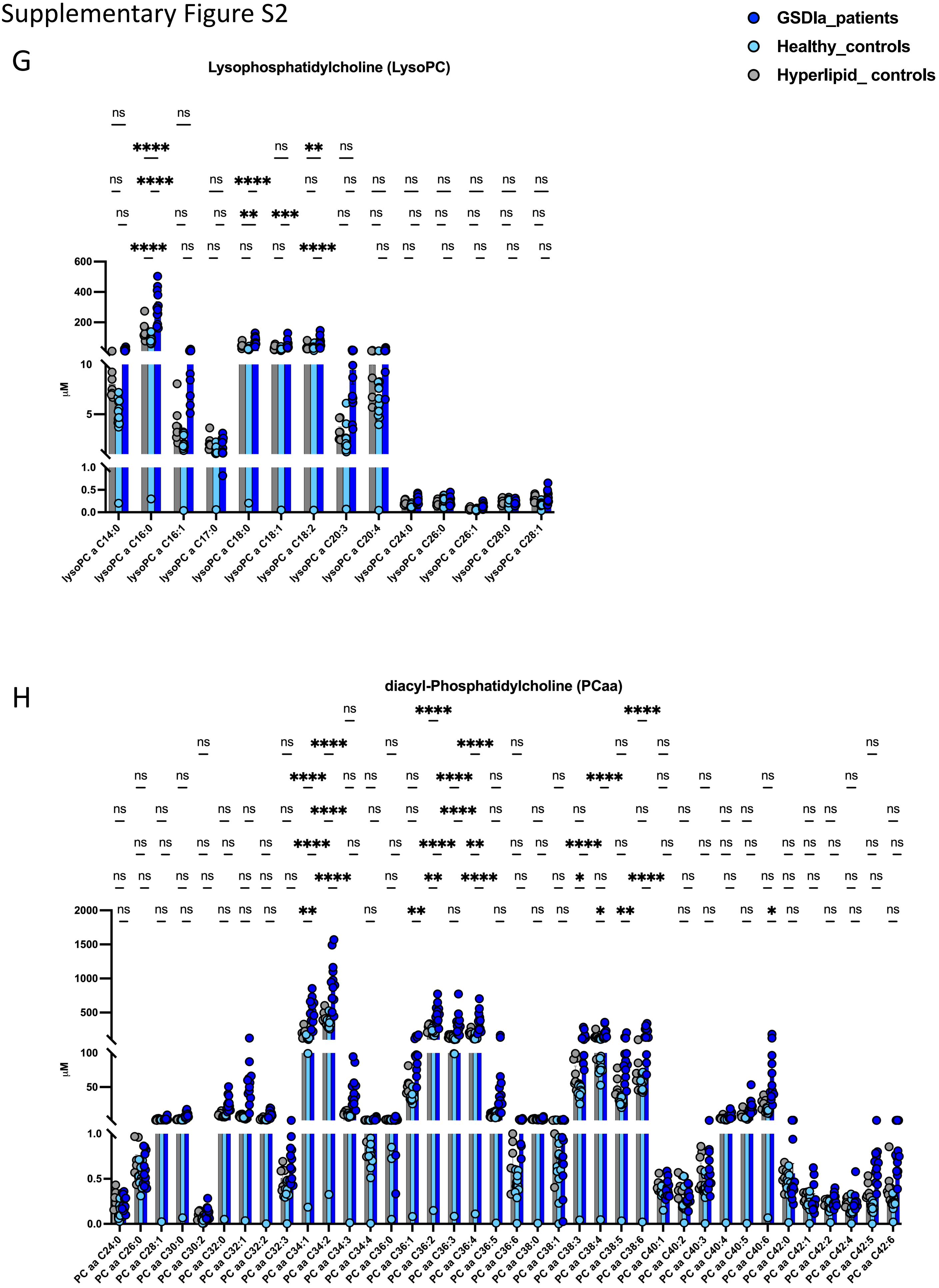

Supplementary Figure S2

GSDIa_patients
Healthy_controls
Hyperlipid_ controls

I

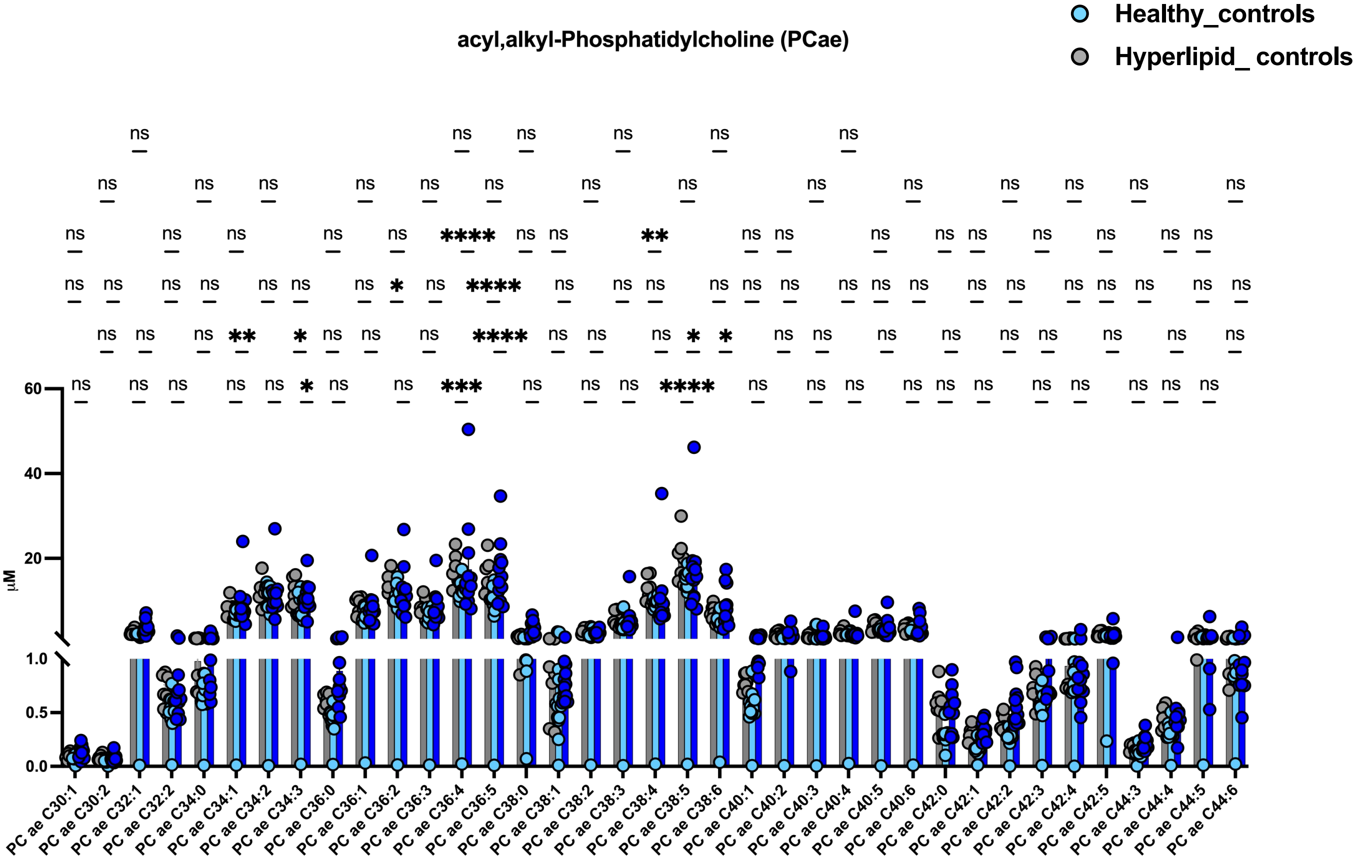

J

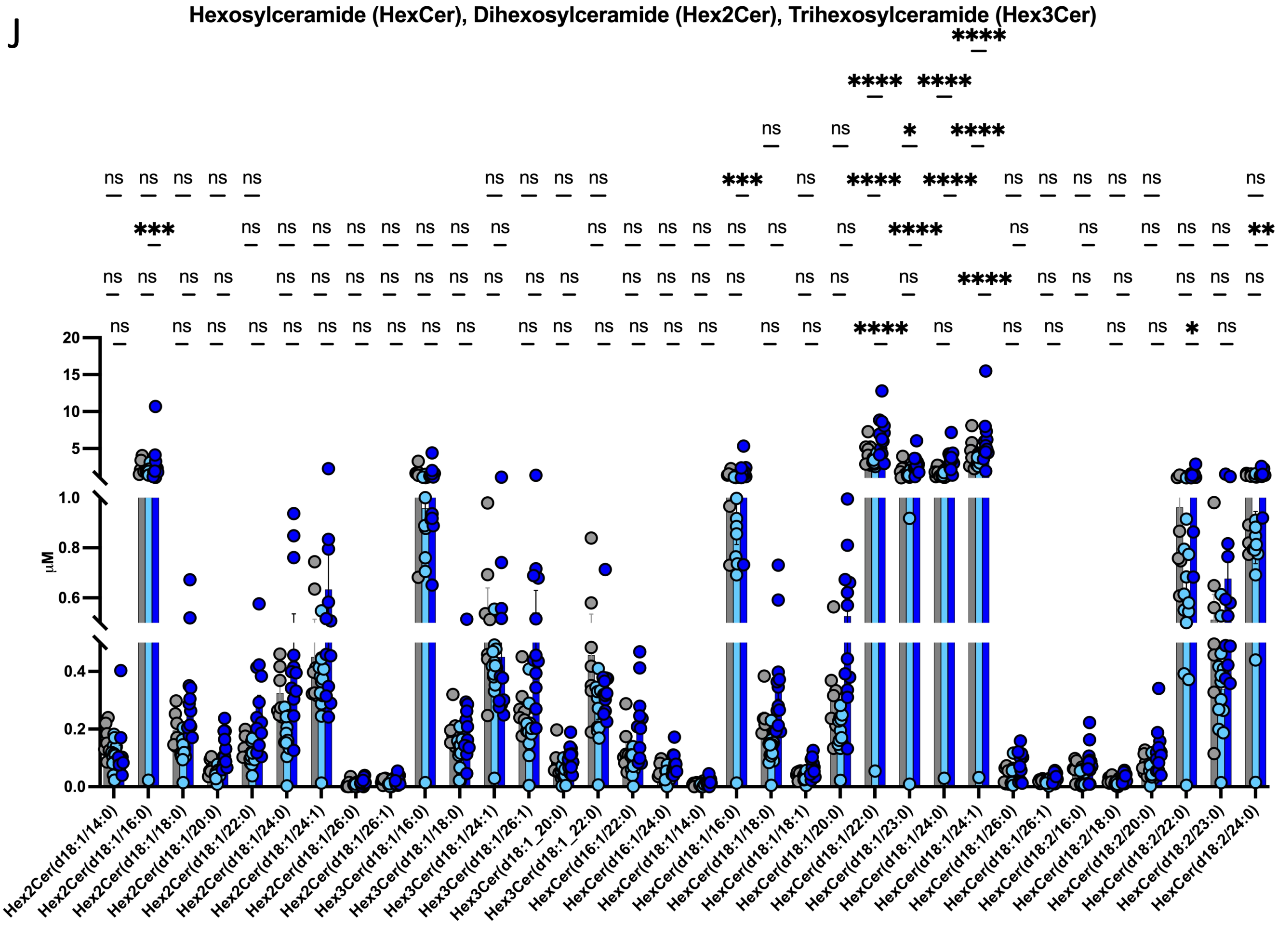

Supplementary Figure S2

K

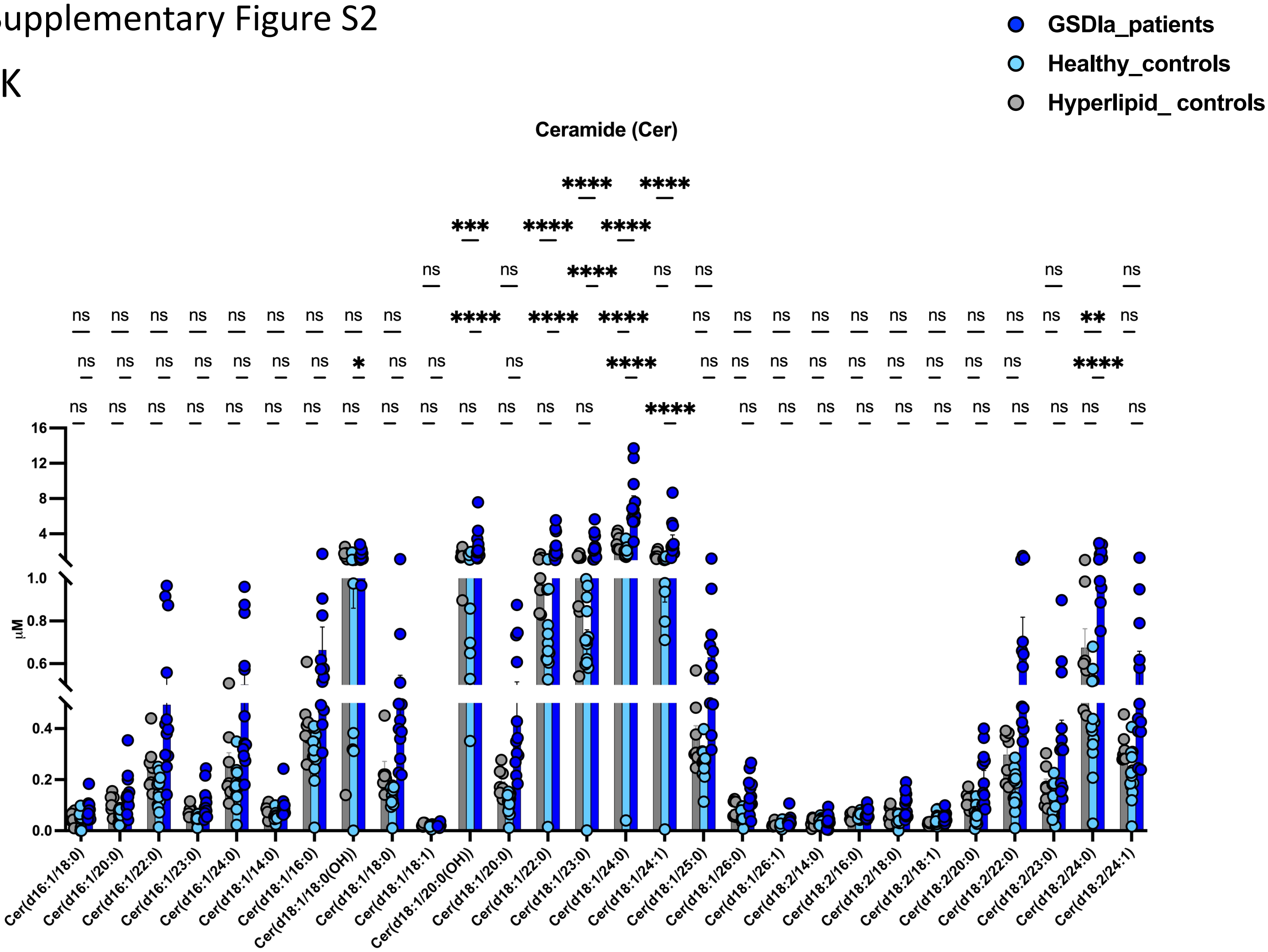

L

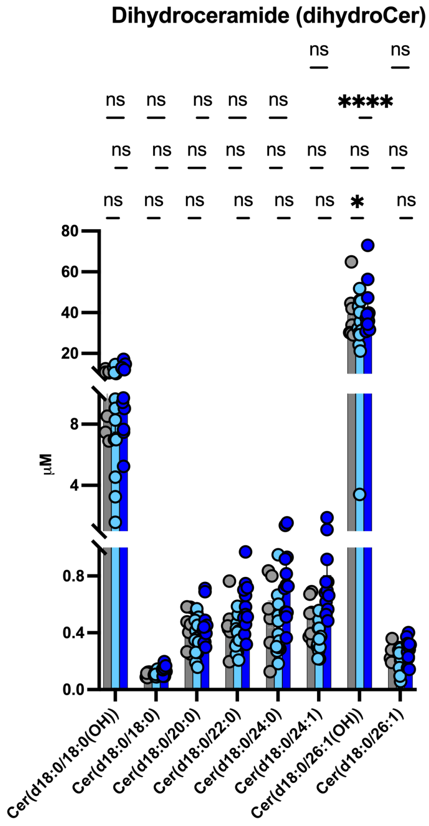

Supplementary Figure S3

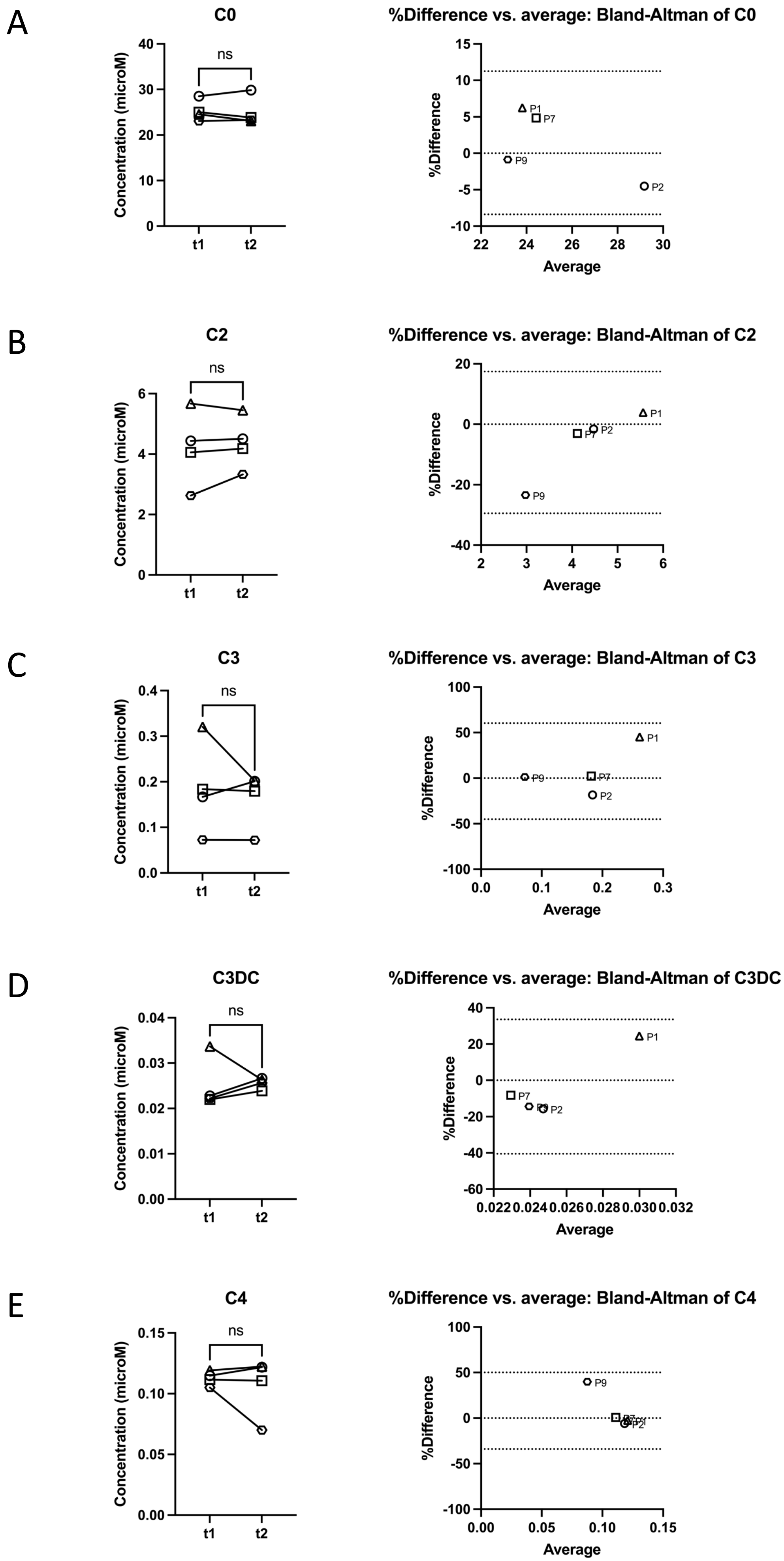

Supplementary Figure S3

F

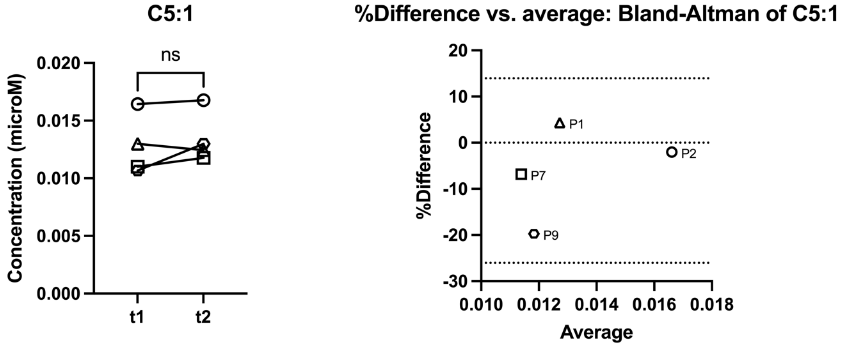

G

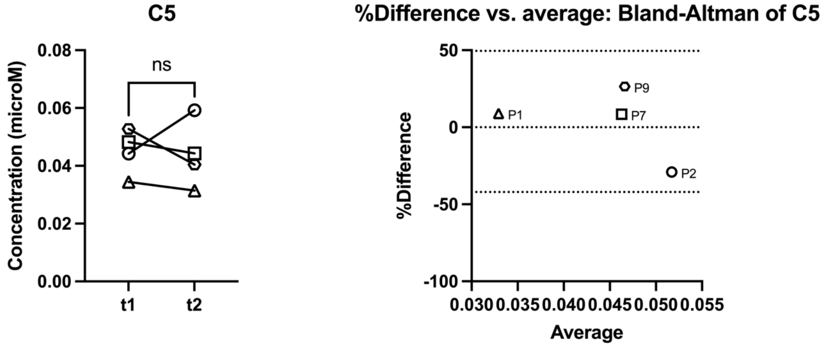

H

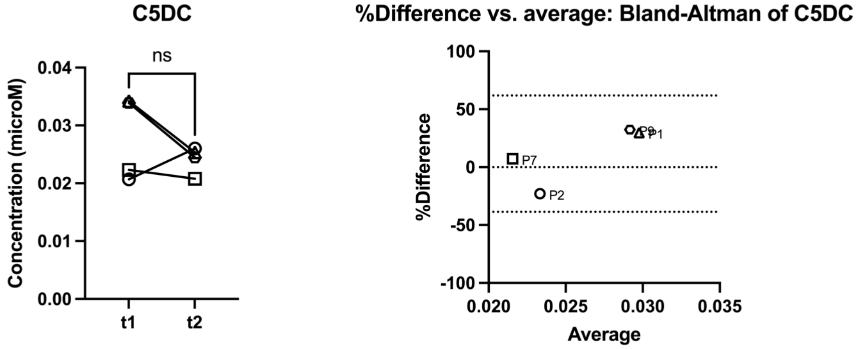

I

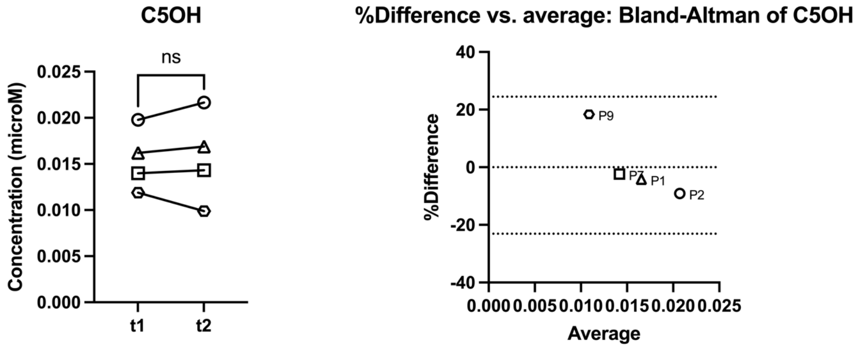

J

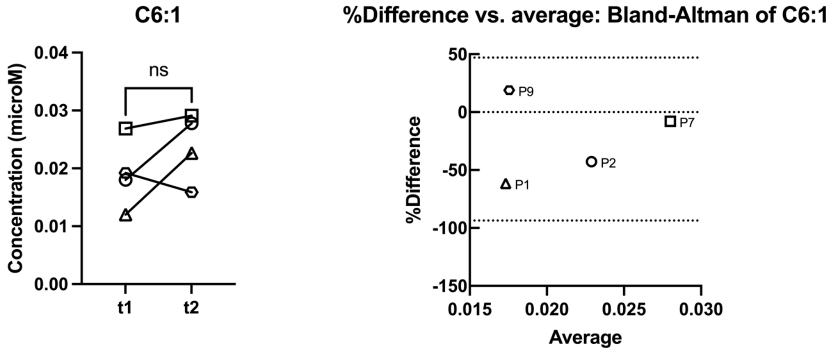

Supplementary Figure S3

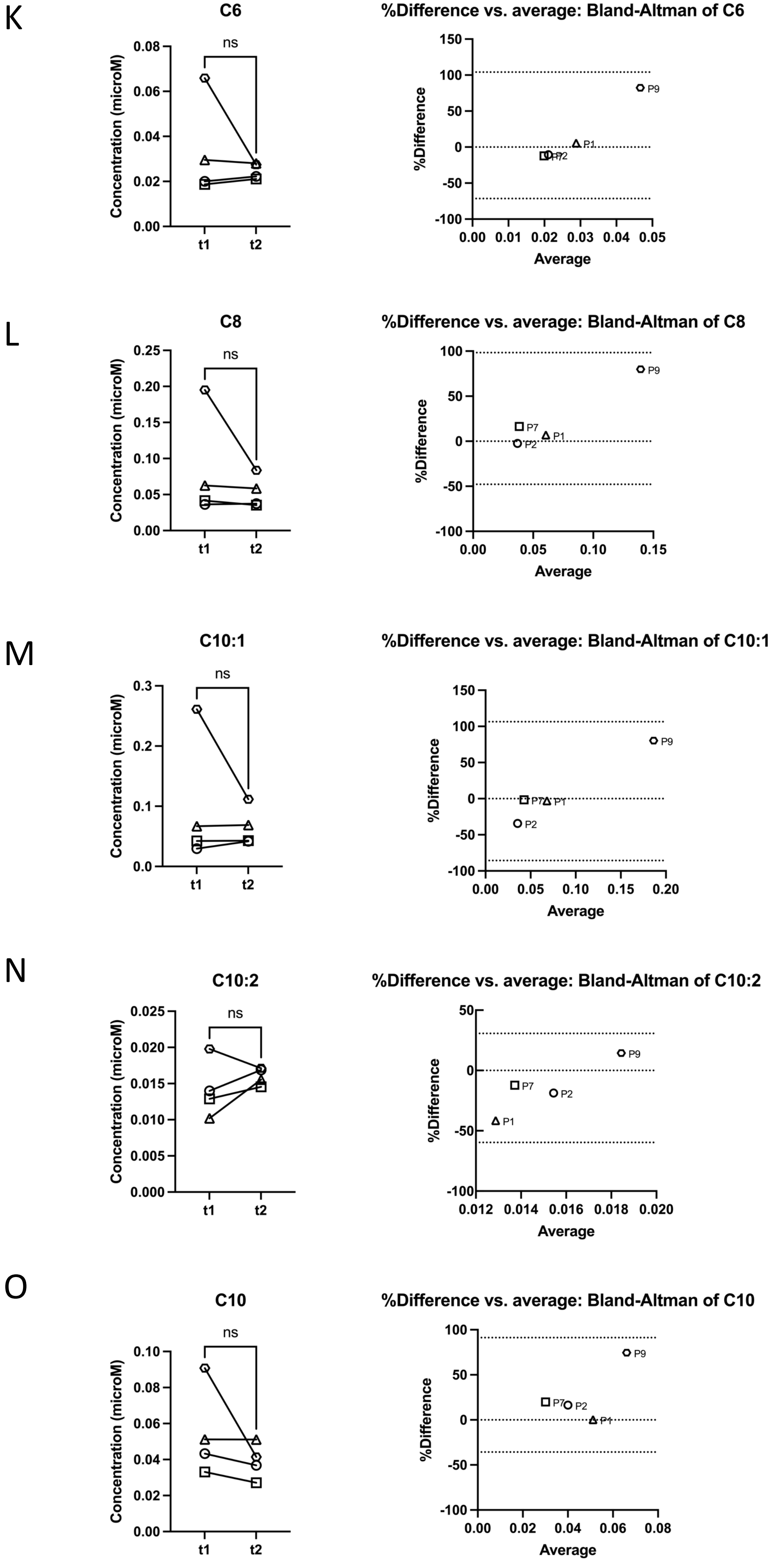

Supplementary Figure S3

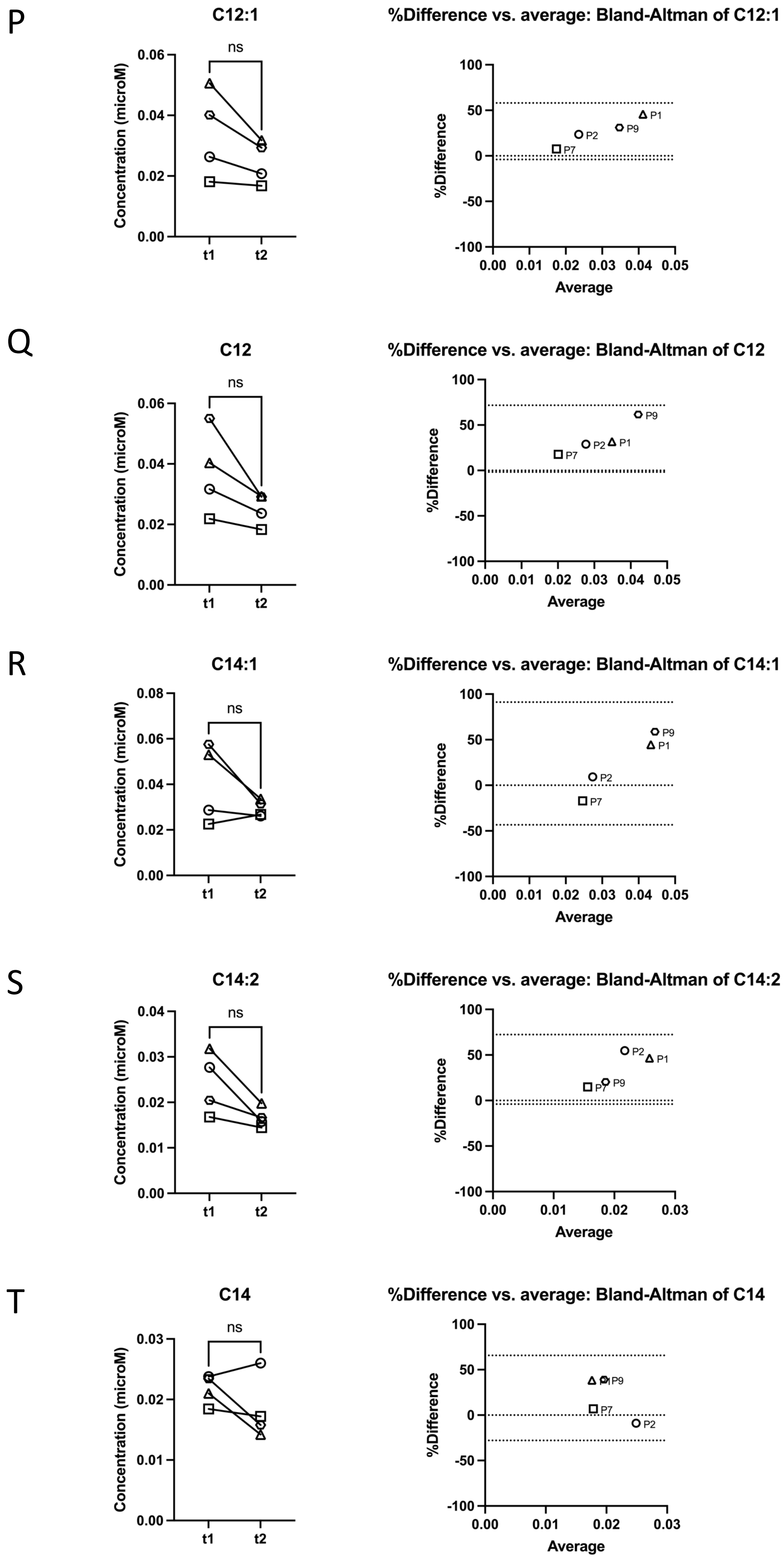

Supplementary Figure S4

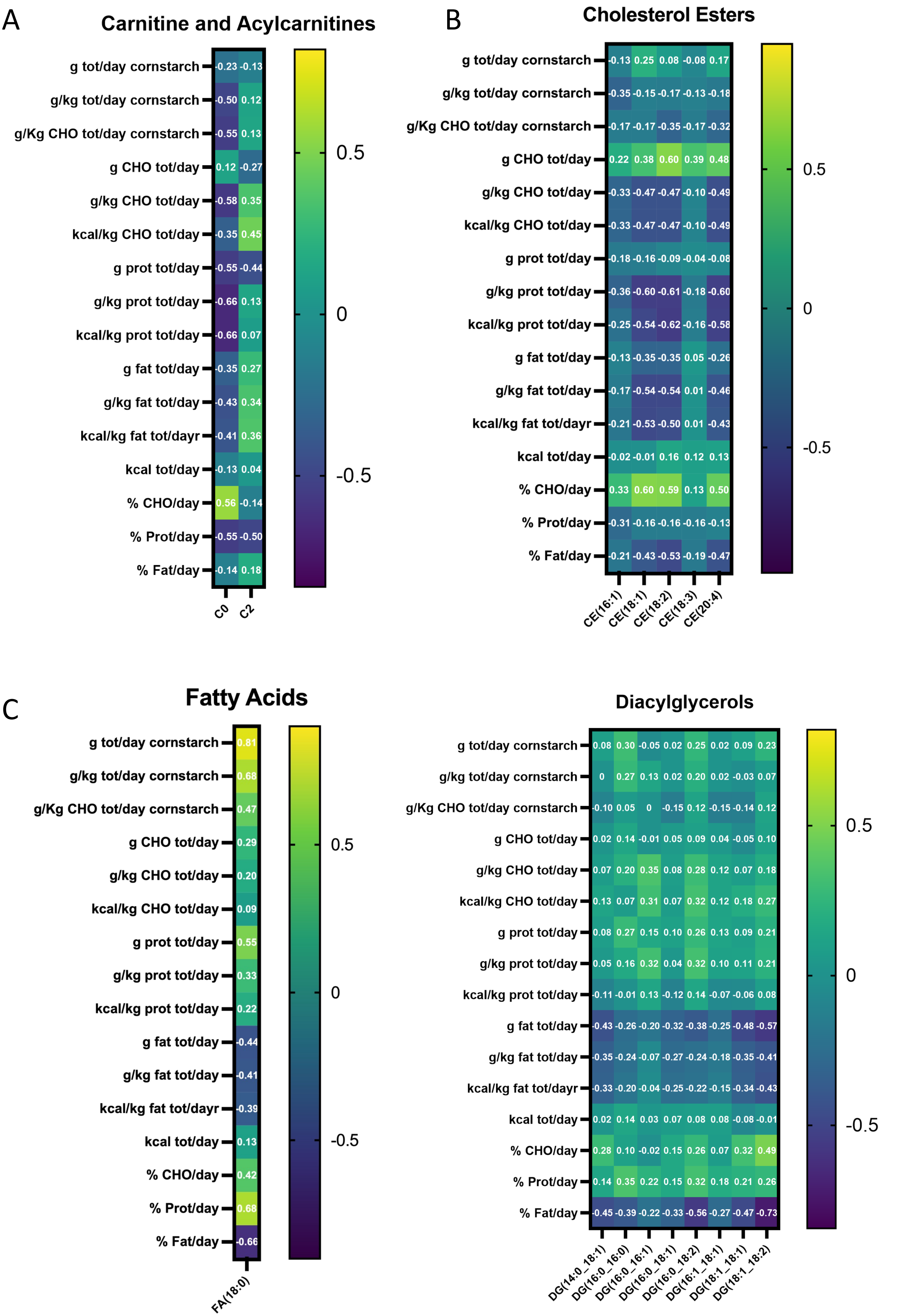

Supplementary Figure S4

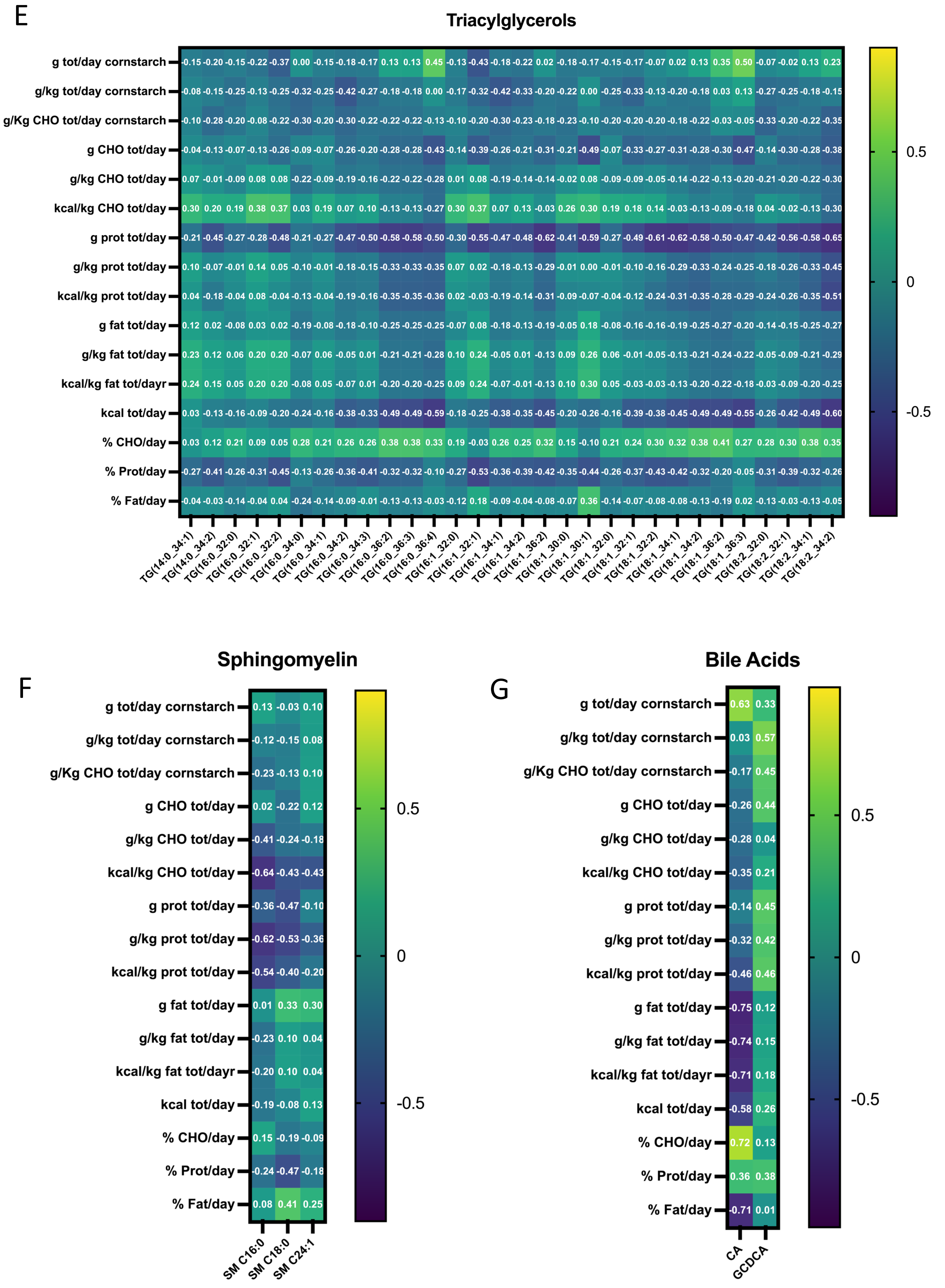

Supplementary Figure S4

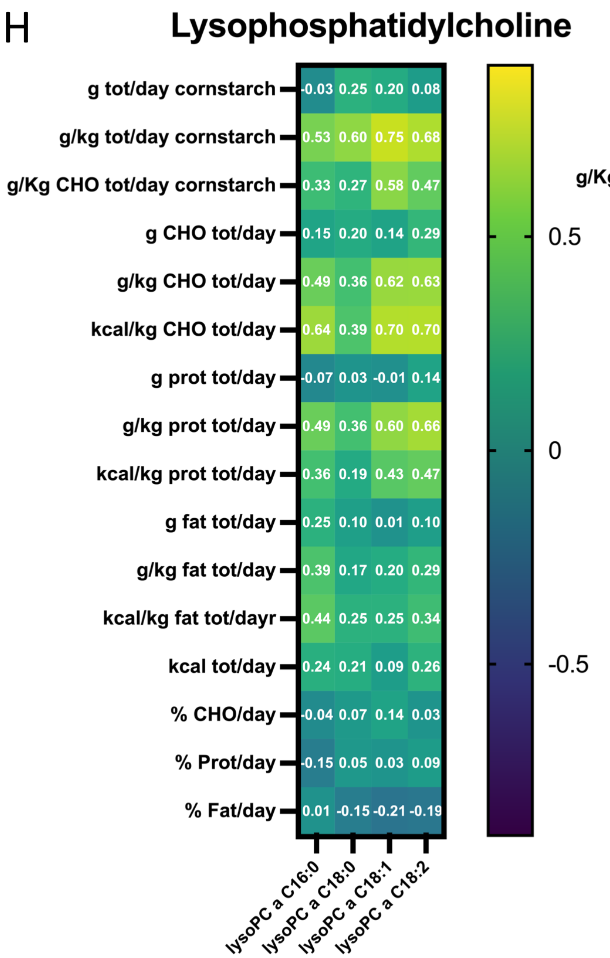

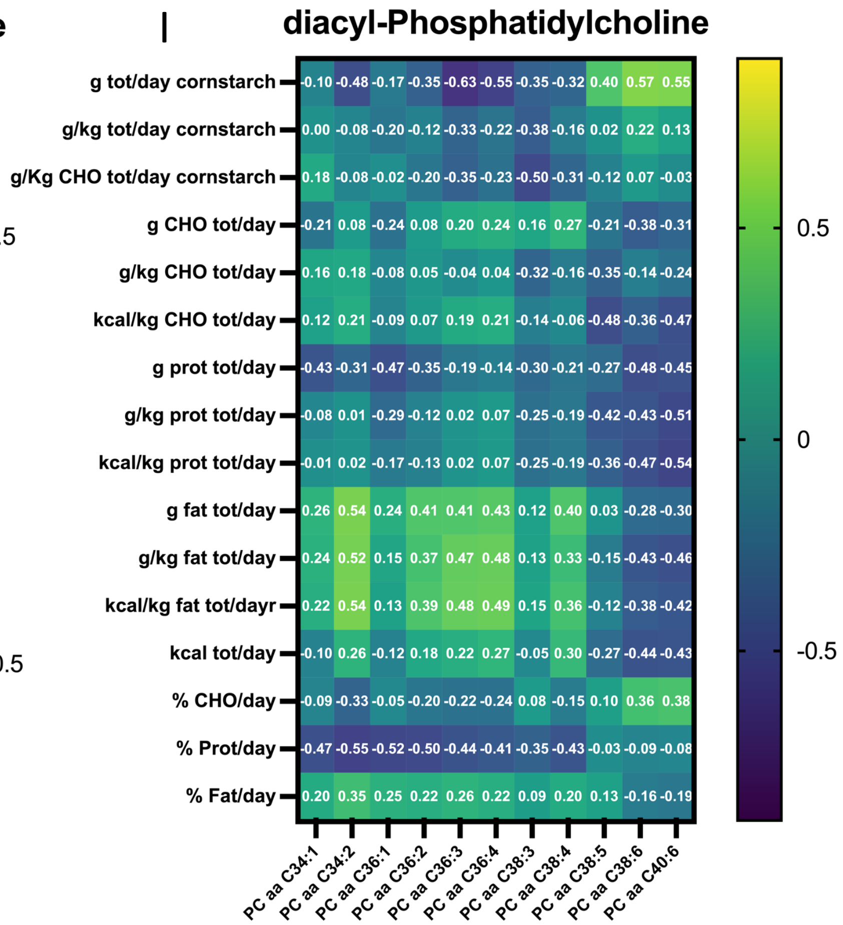

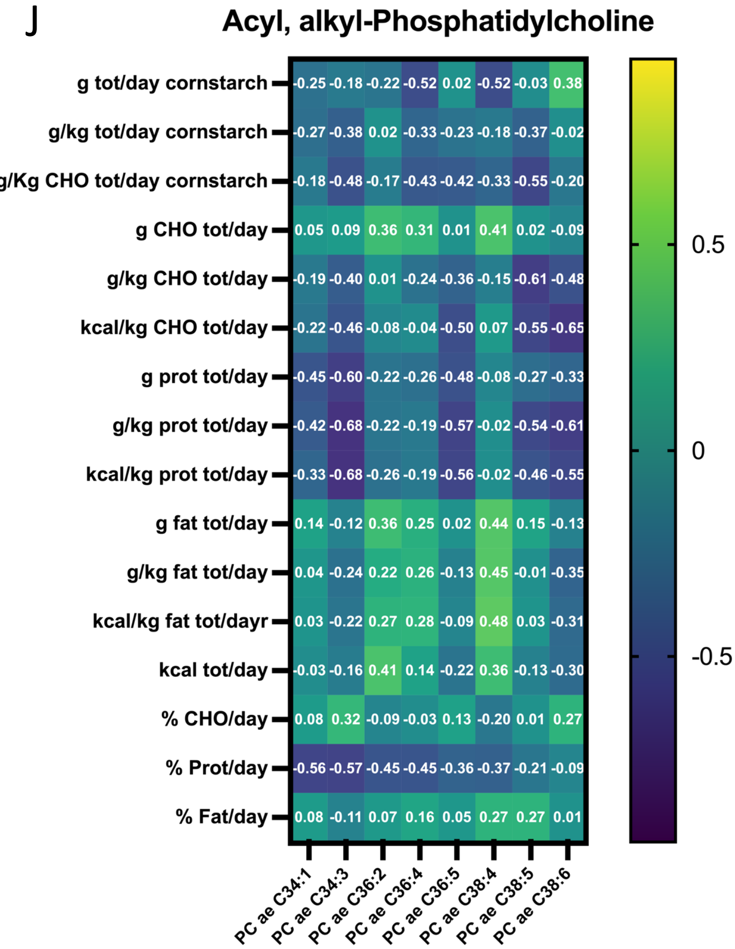

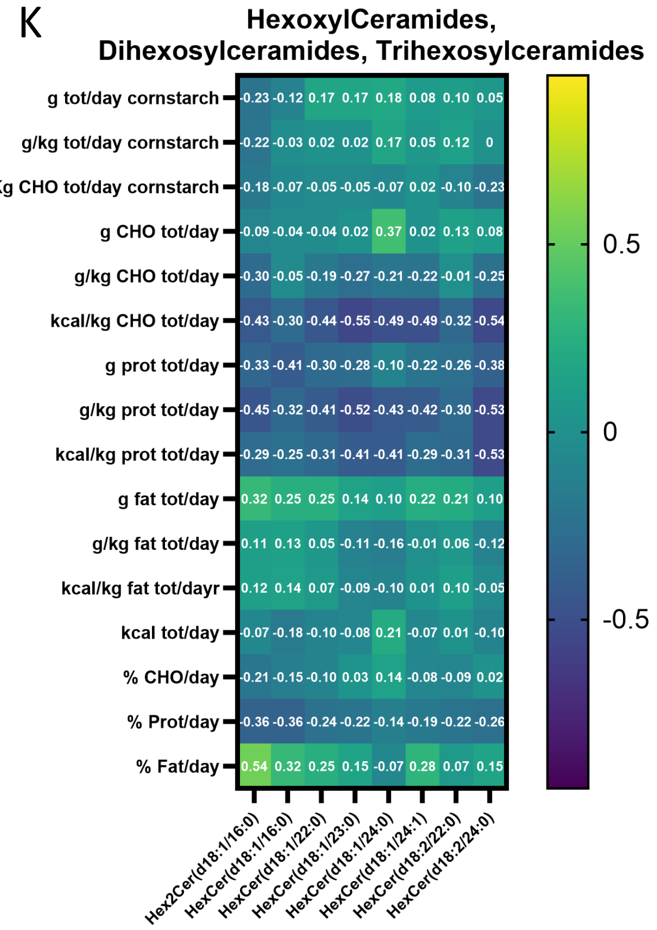

Supplementary Figure S4

L

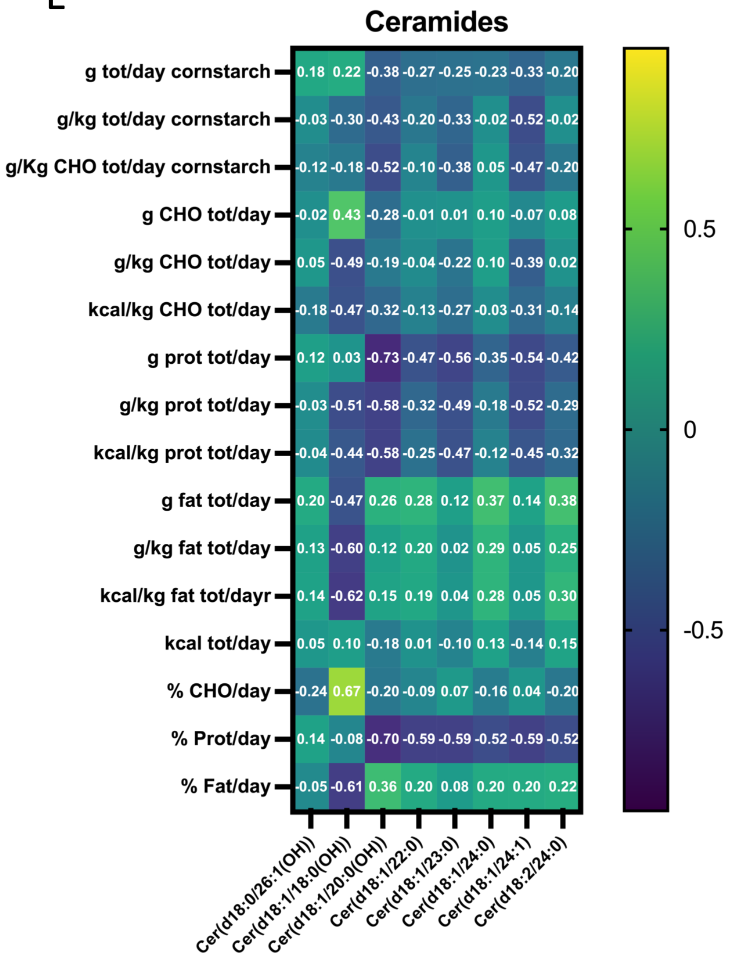

Supplementary Figure S5

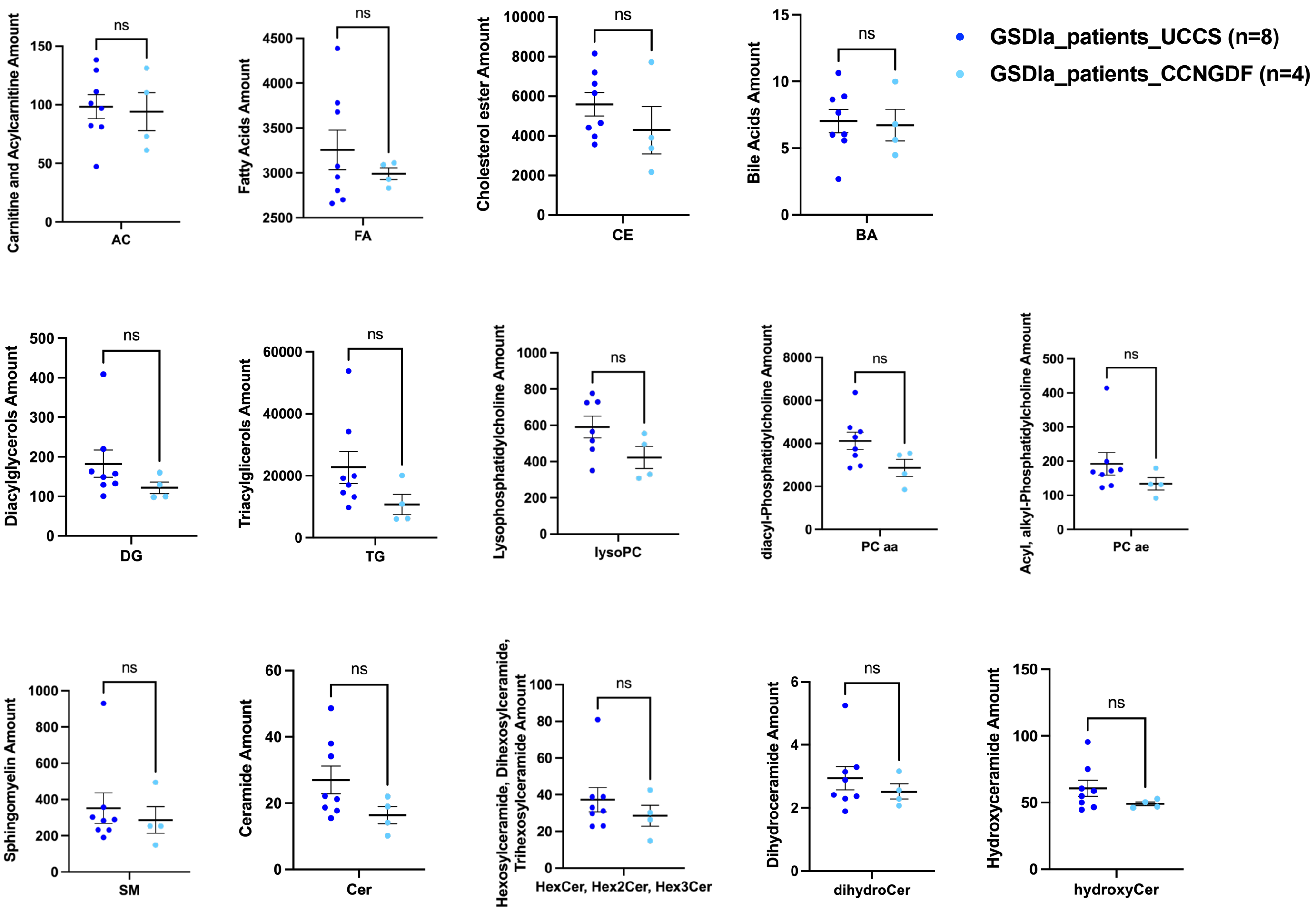

Supplementary Figure S6

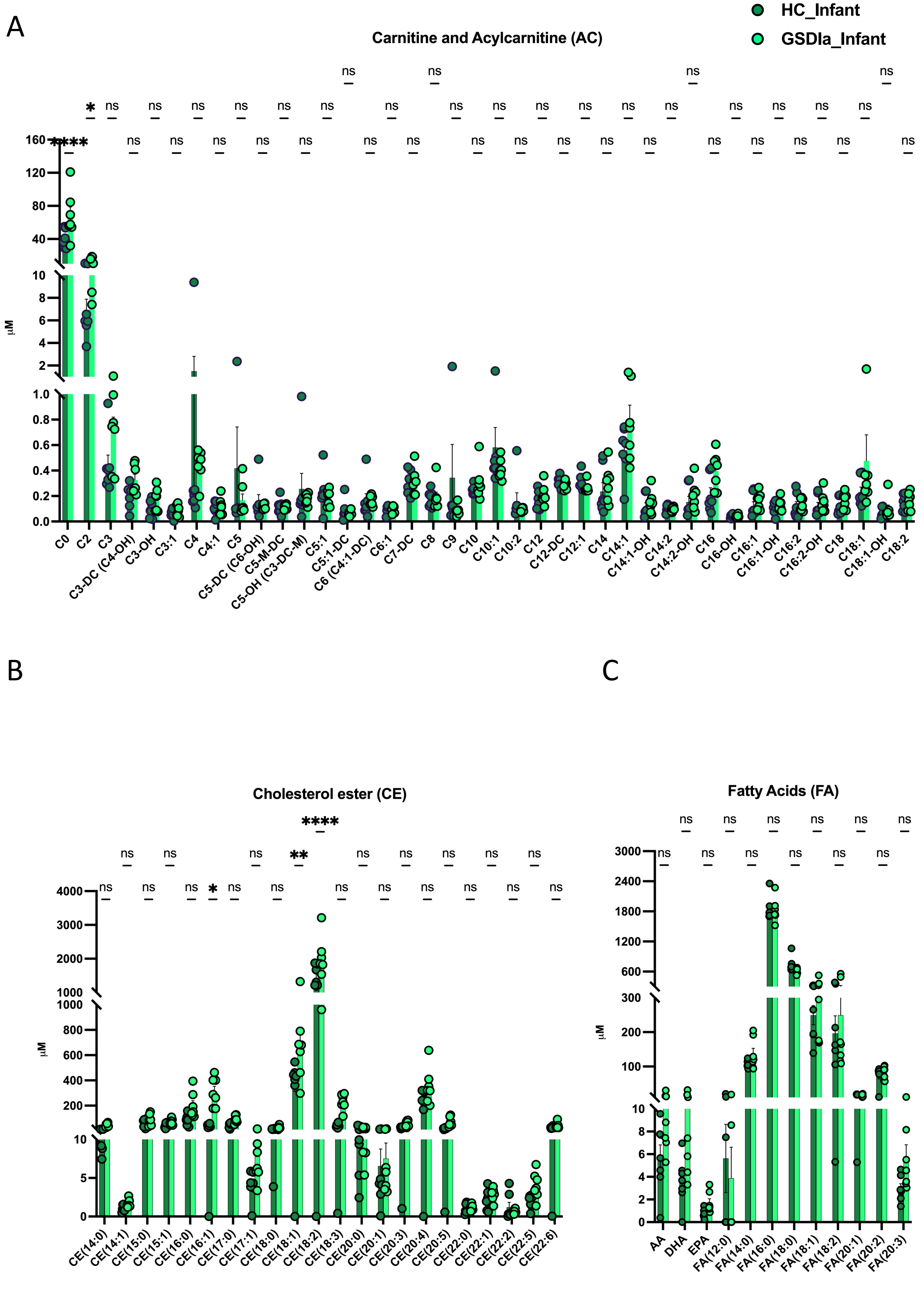

Supplementary Figure S6

D

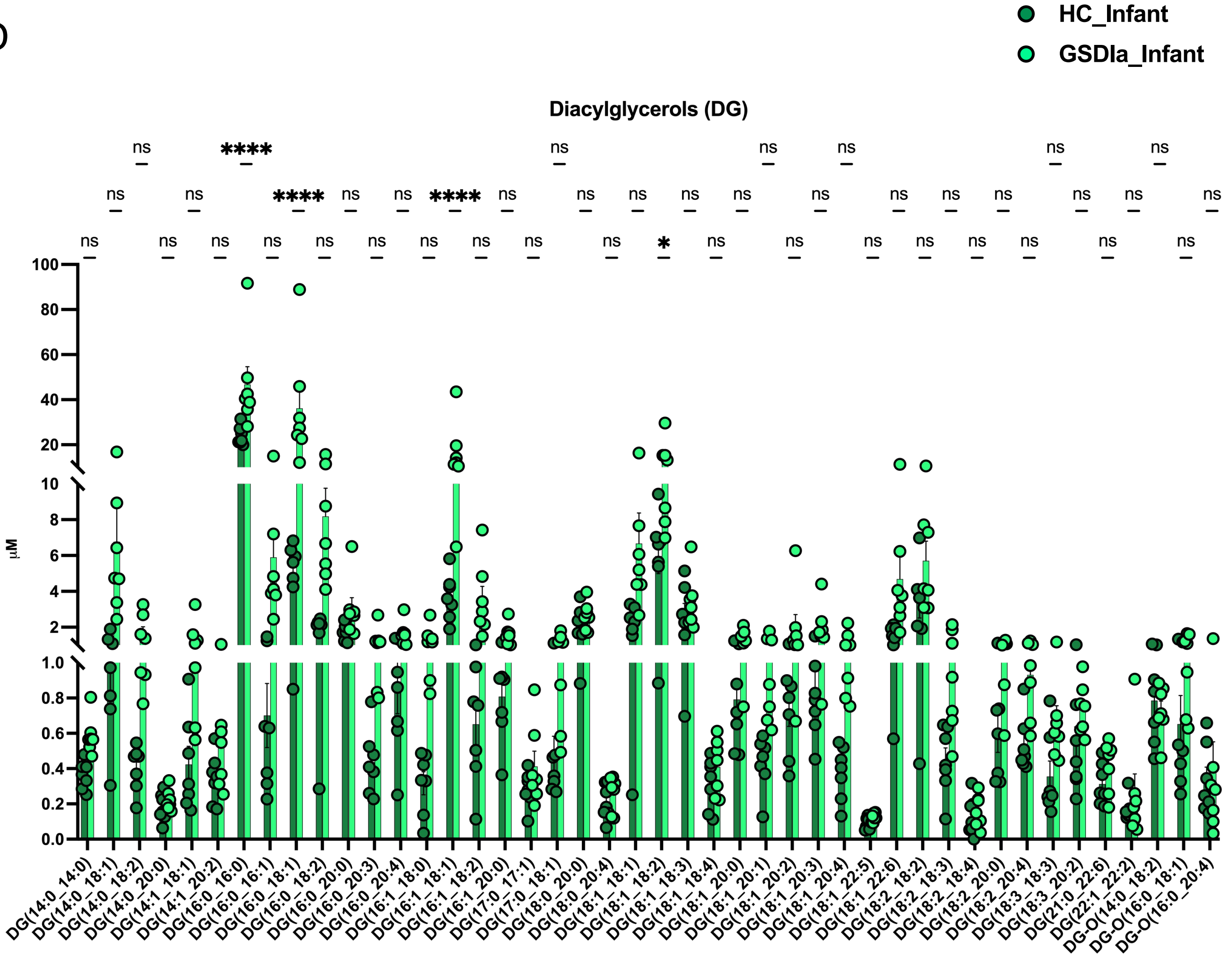

E

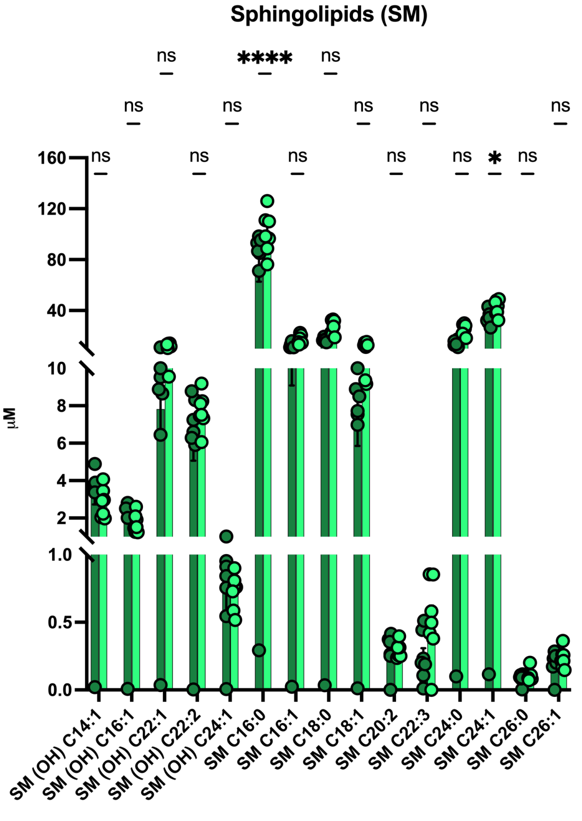

F

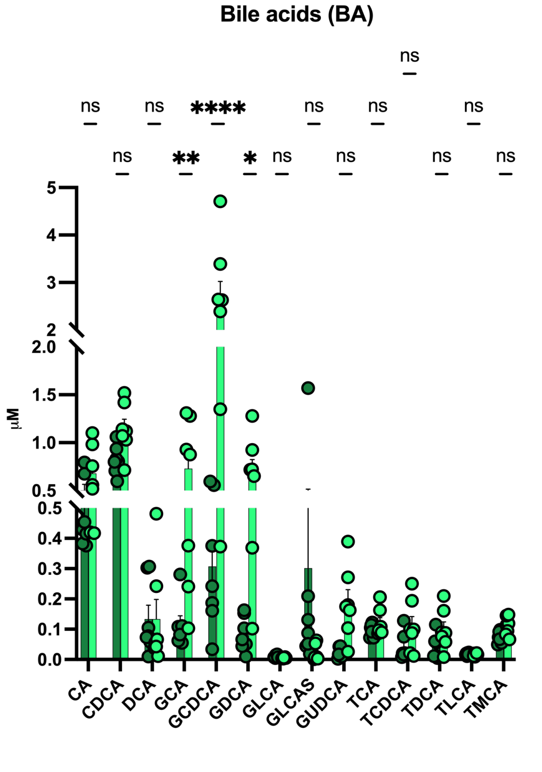

Supplementary Figure S6

G

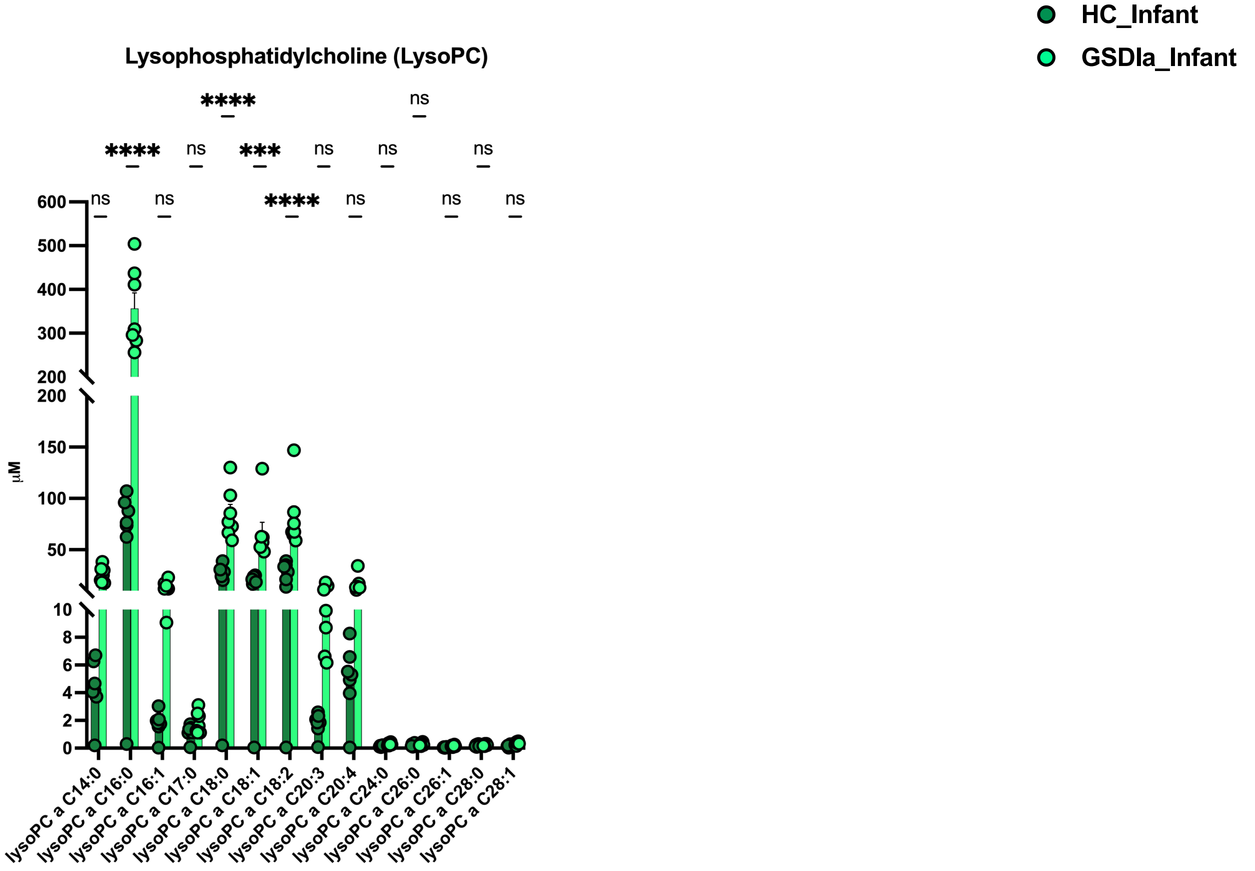

H

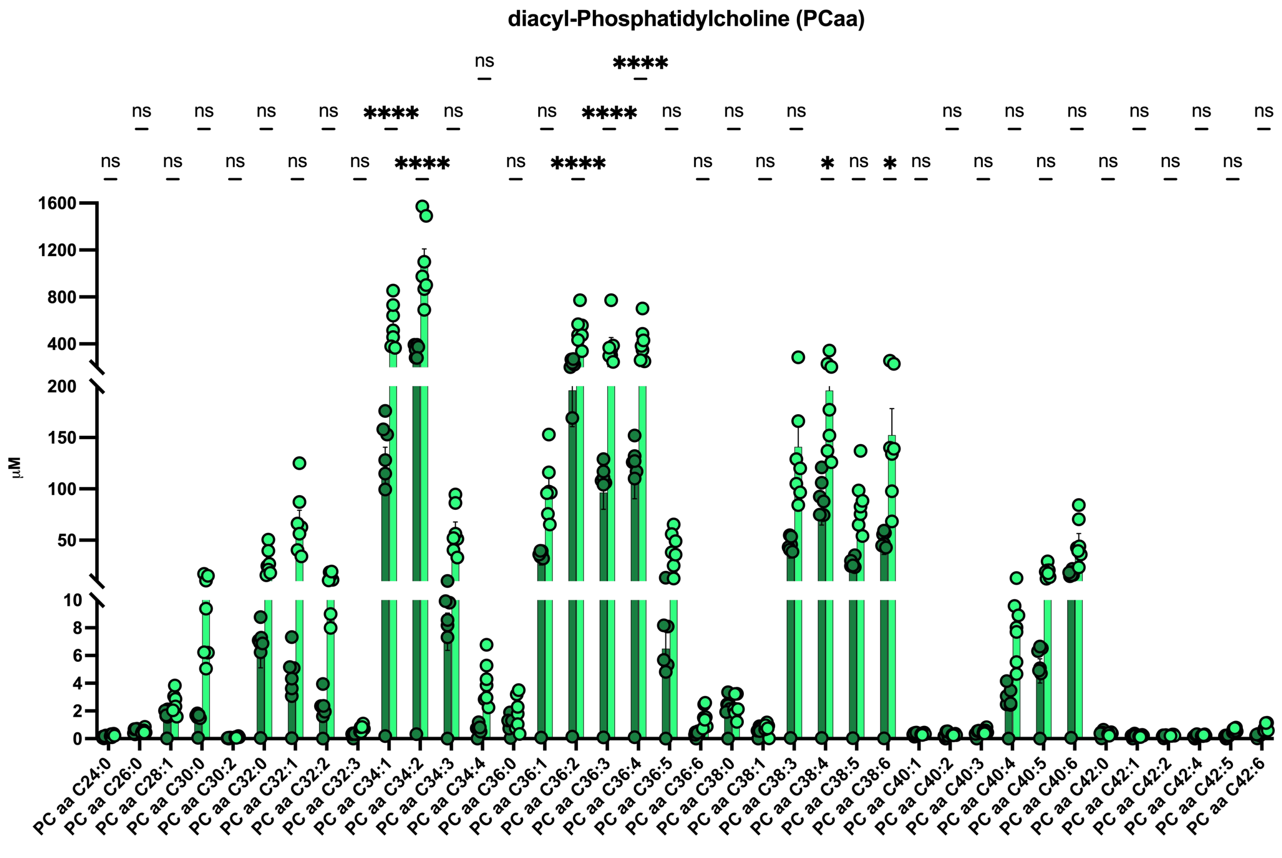

Supplementary Figure S6

I

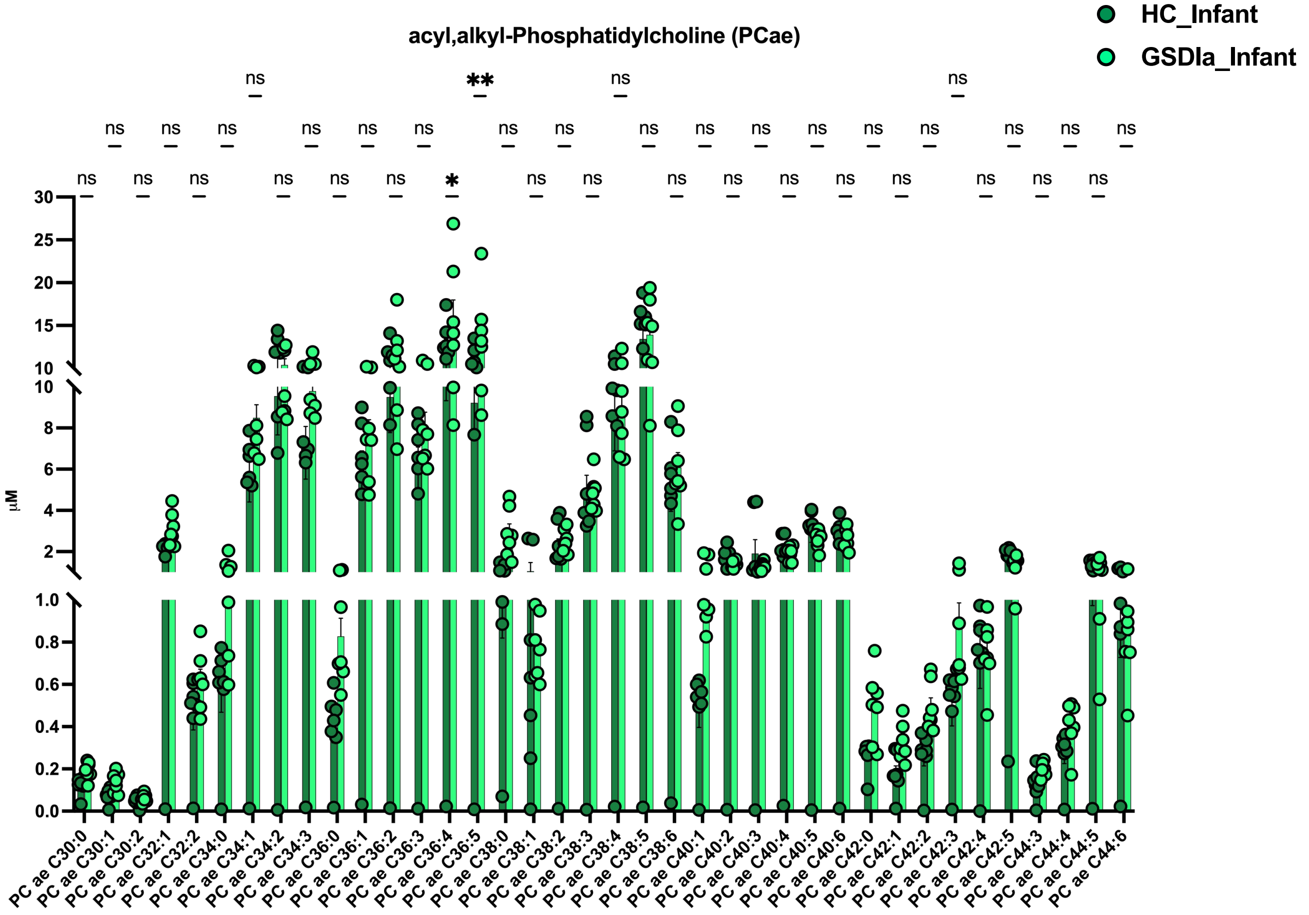

J

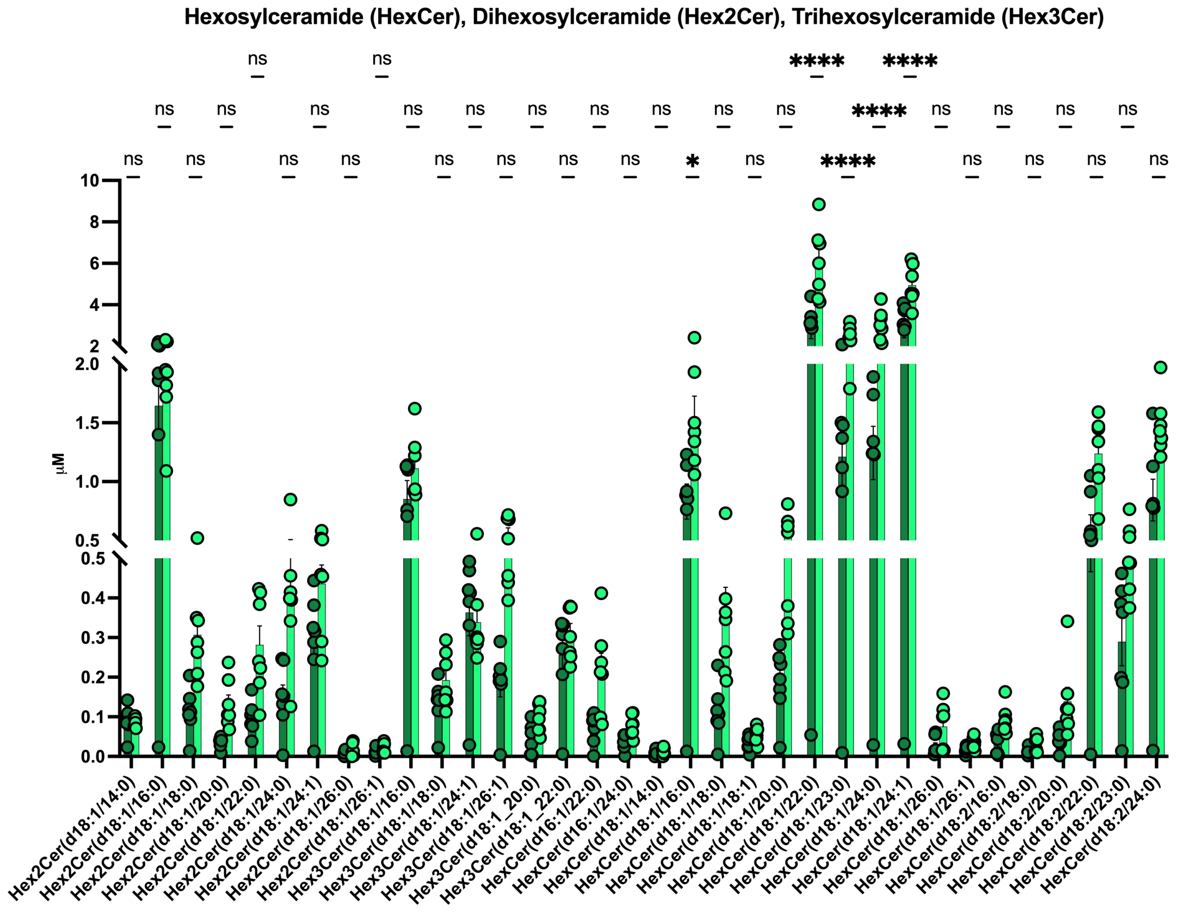

Supplementary Figure S6

K

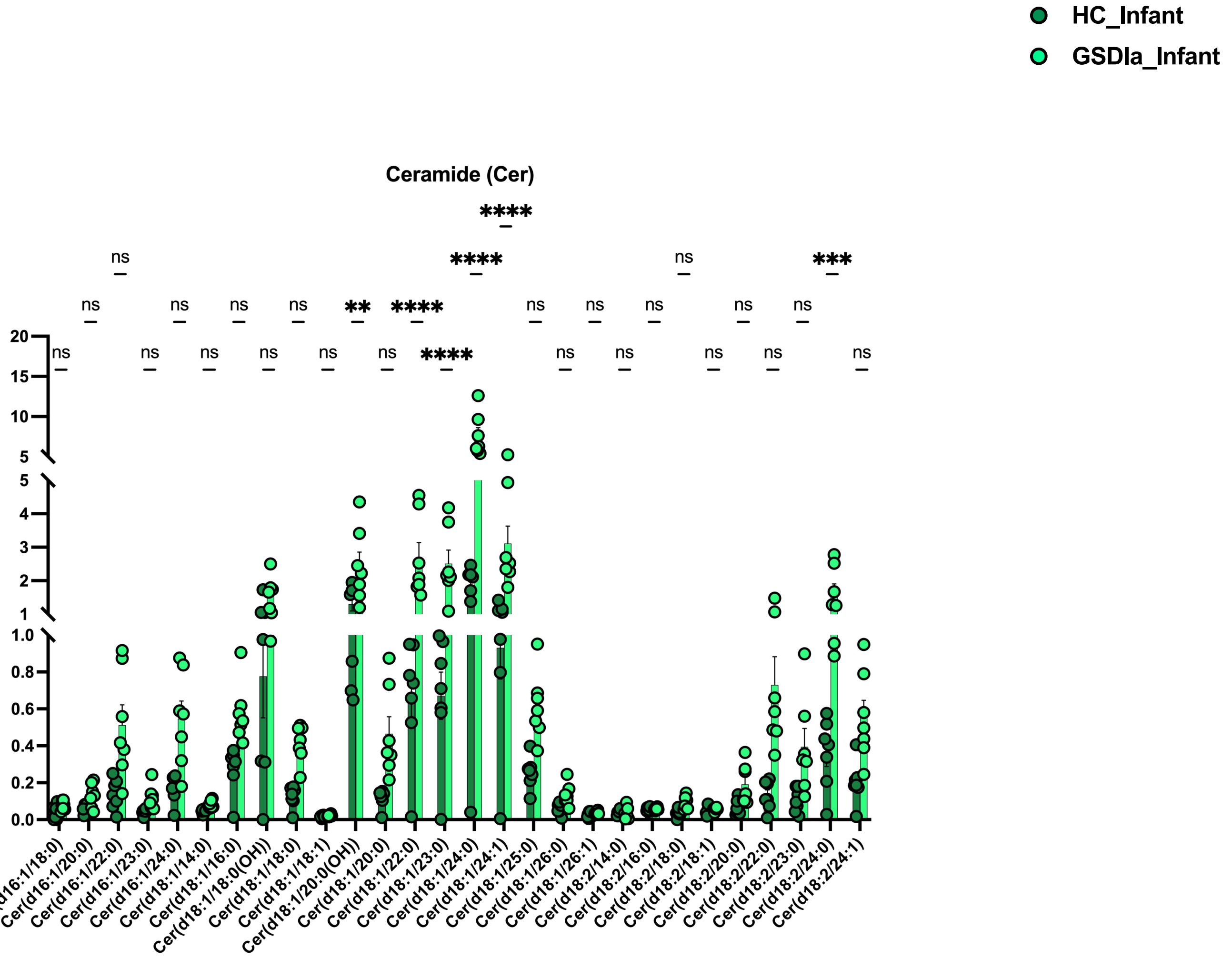

L

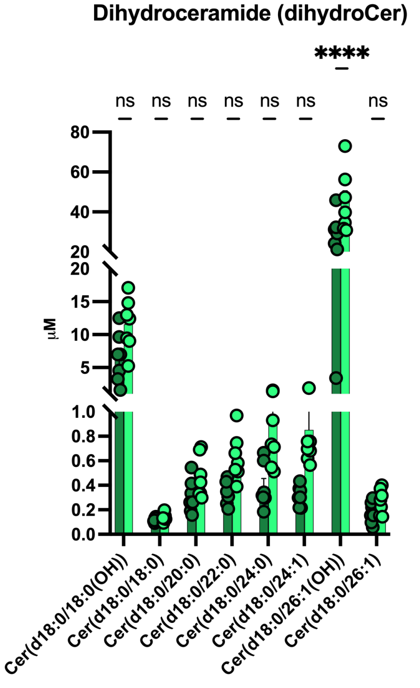

Supplementary Figure S7

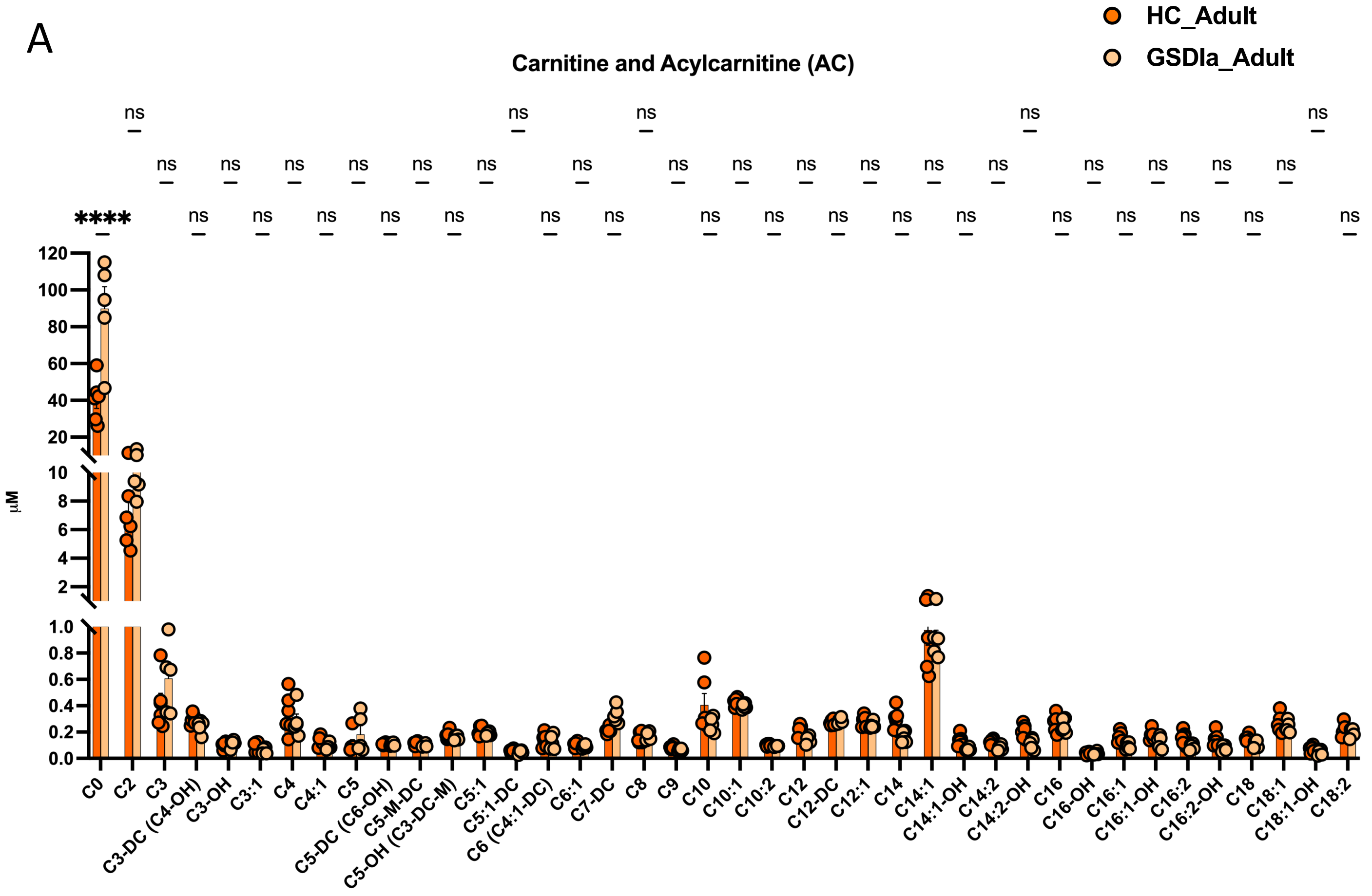

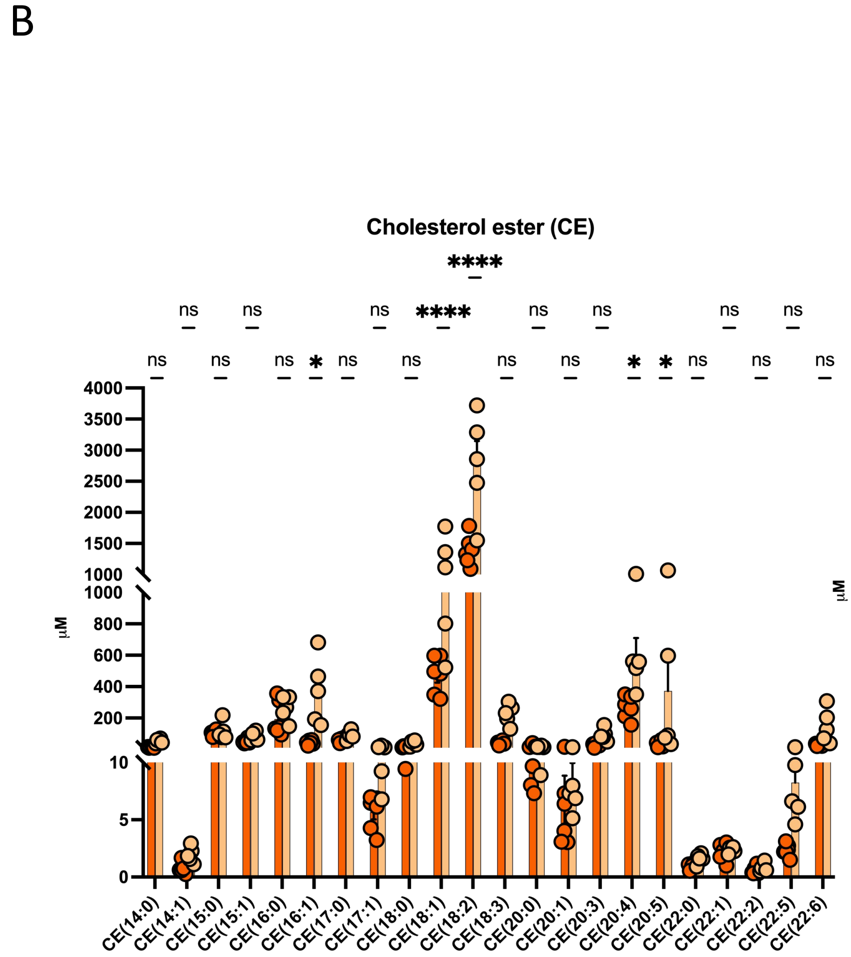

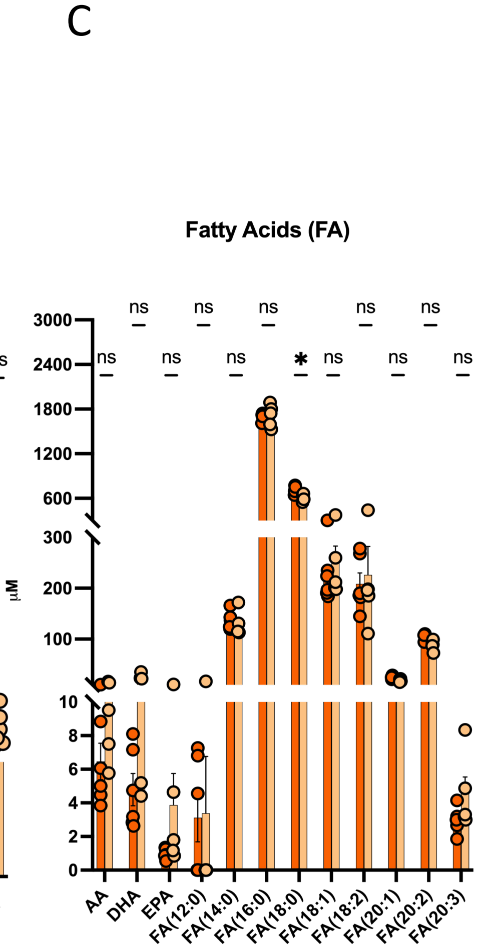

Supplementary Figure S7

D

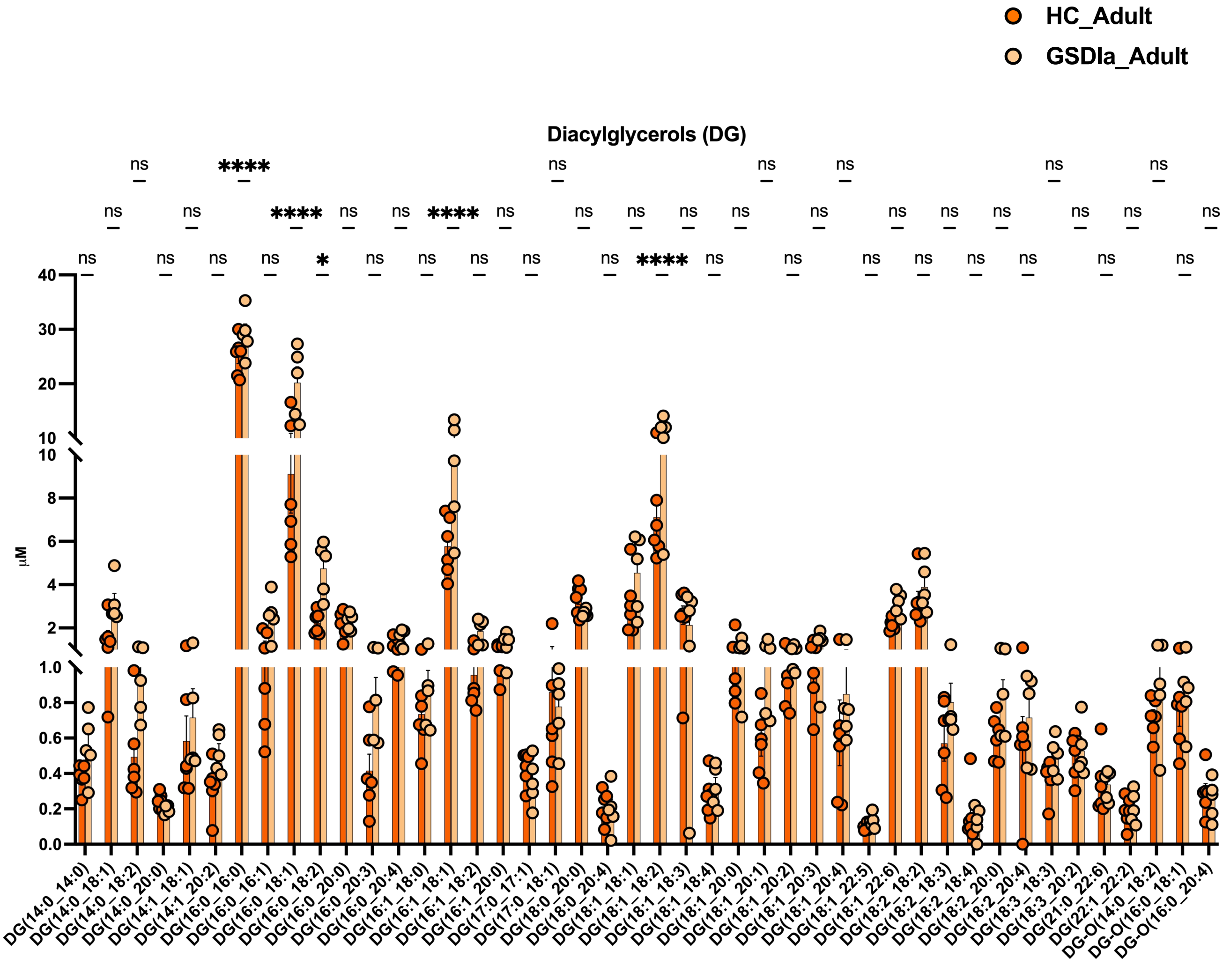

E

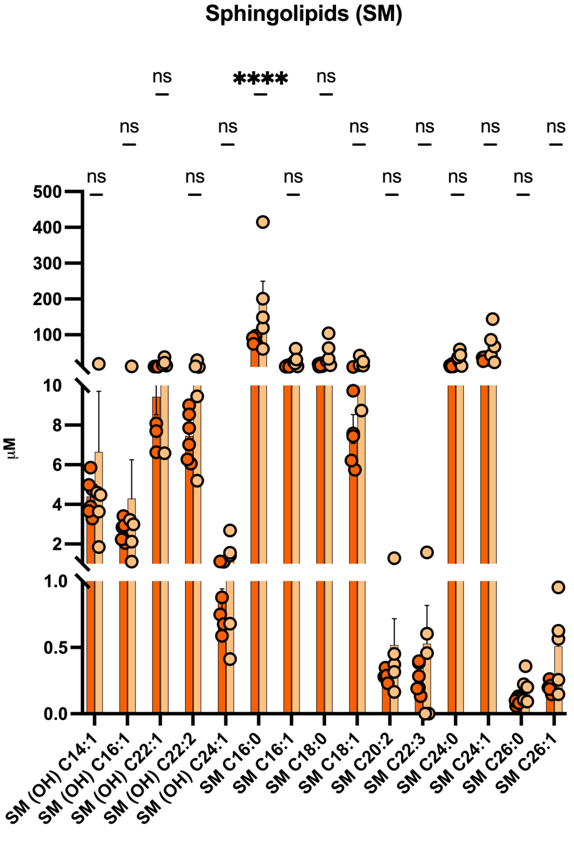

F

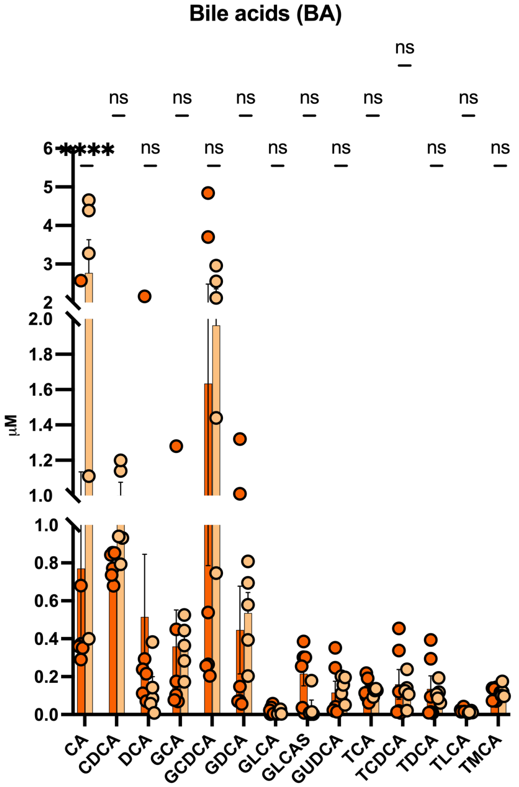

Supplementary Figure S7

G

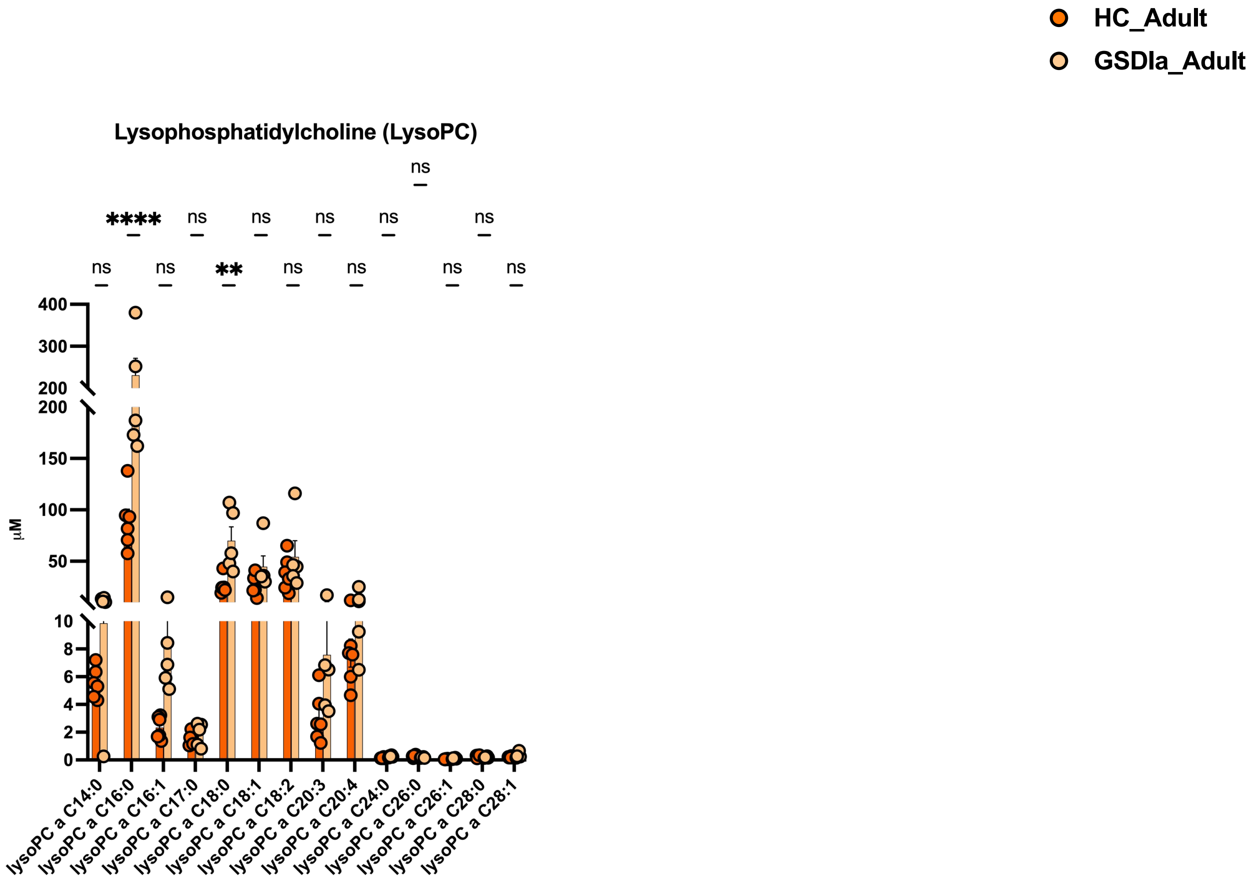

H

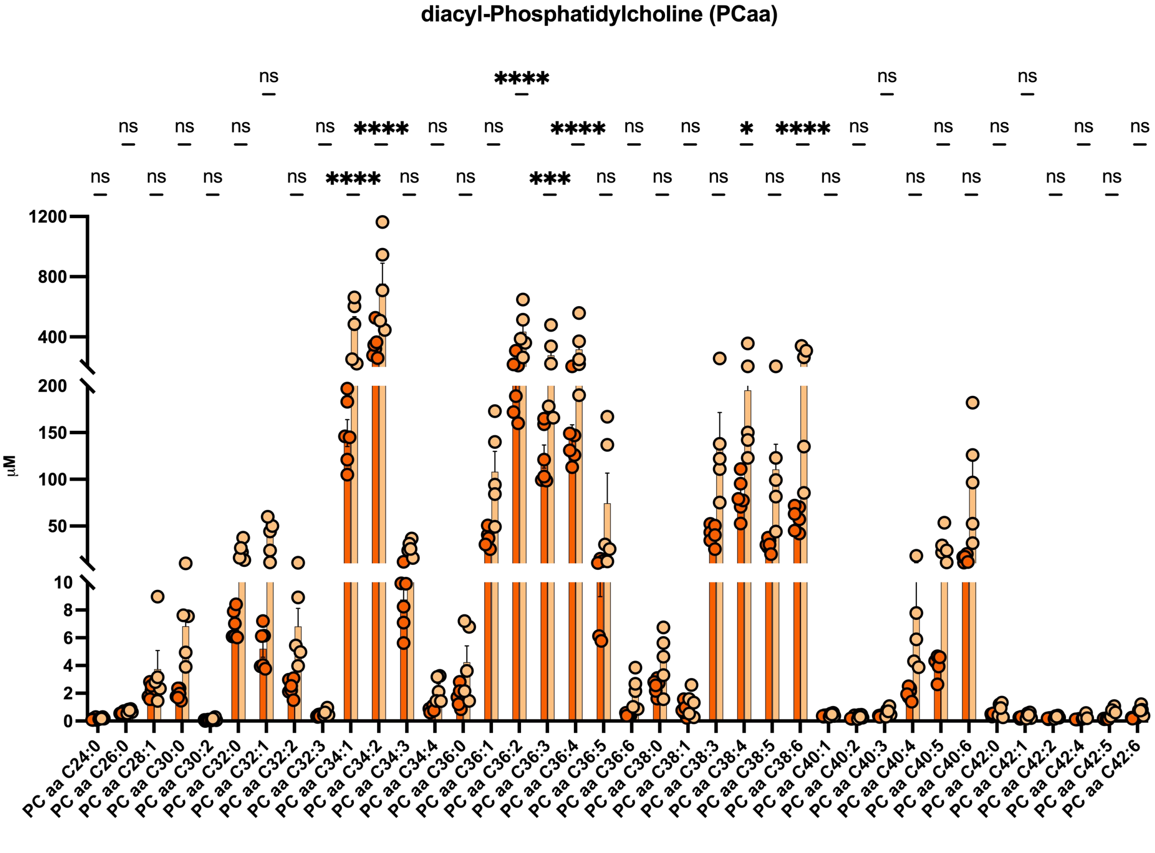

Supplementary Figure S7

I

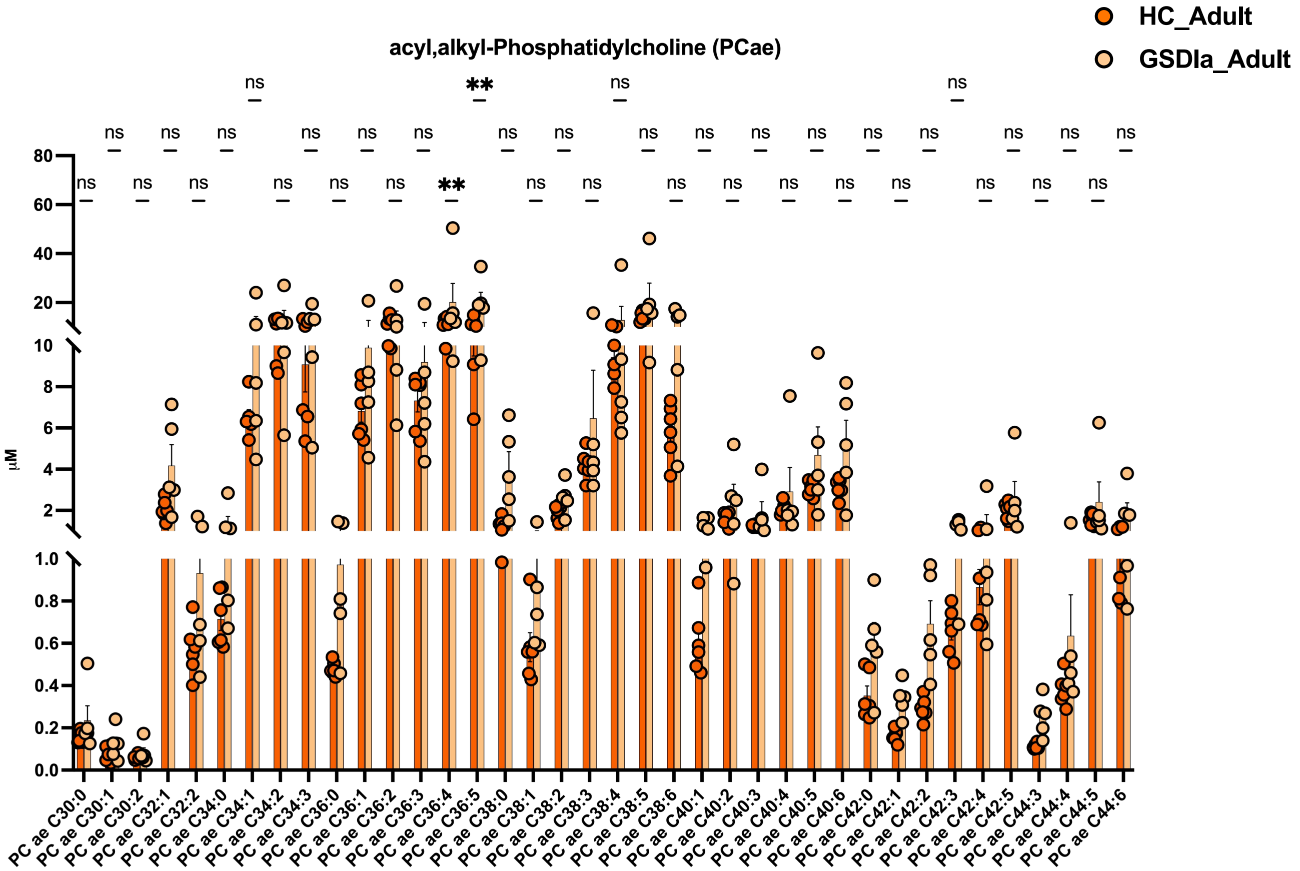

J

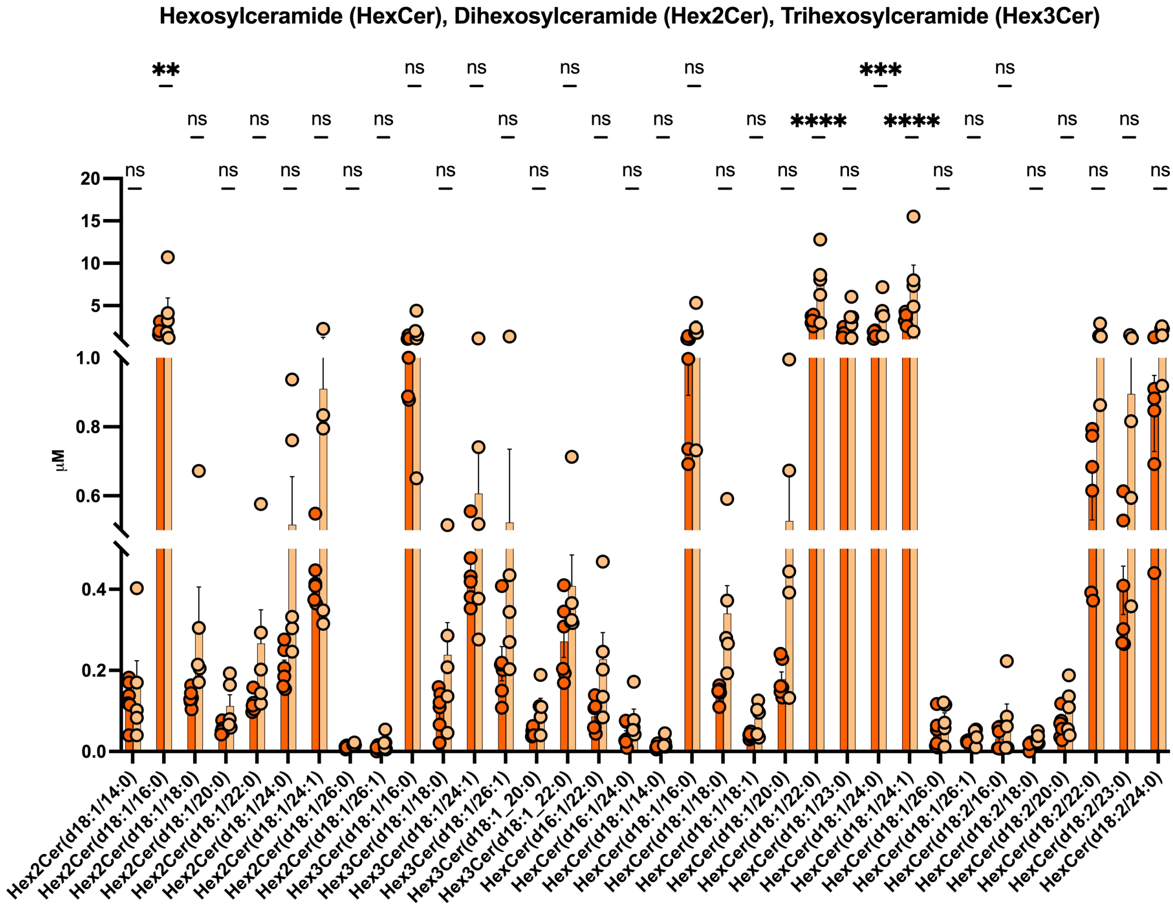

Supplementary Figure S7

K

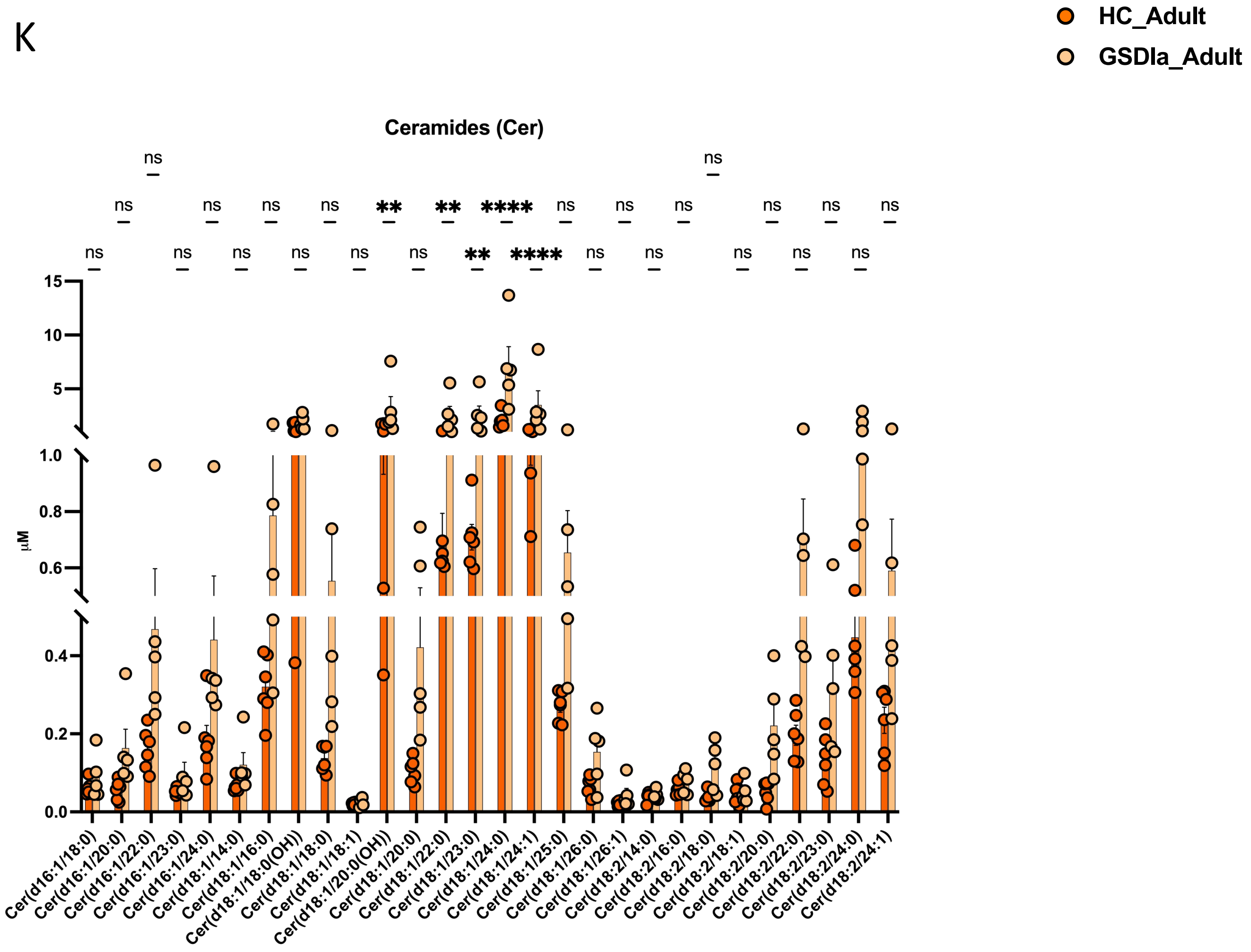

L

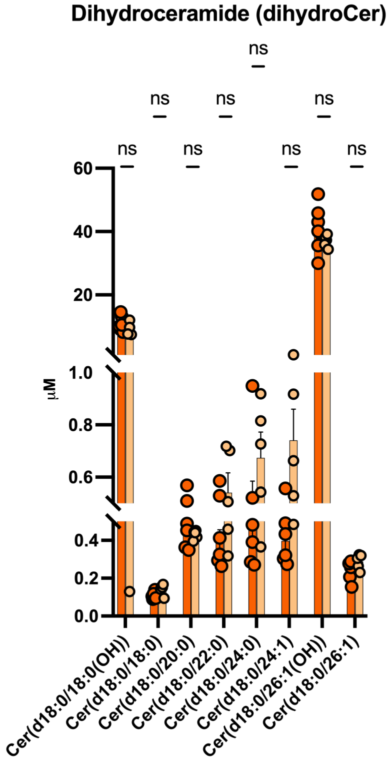

Supplementary Figure S8

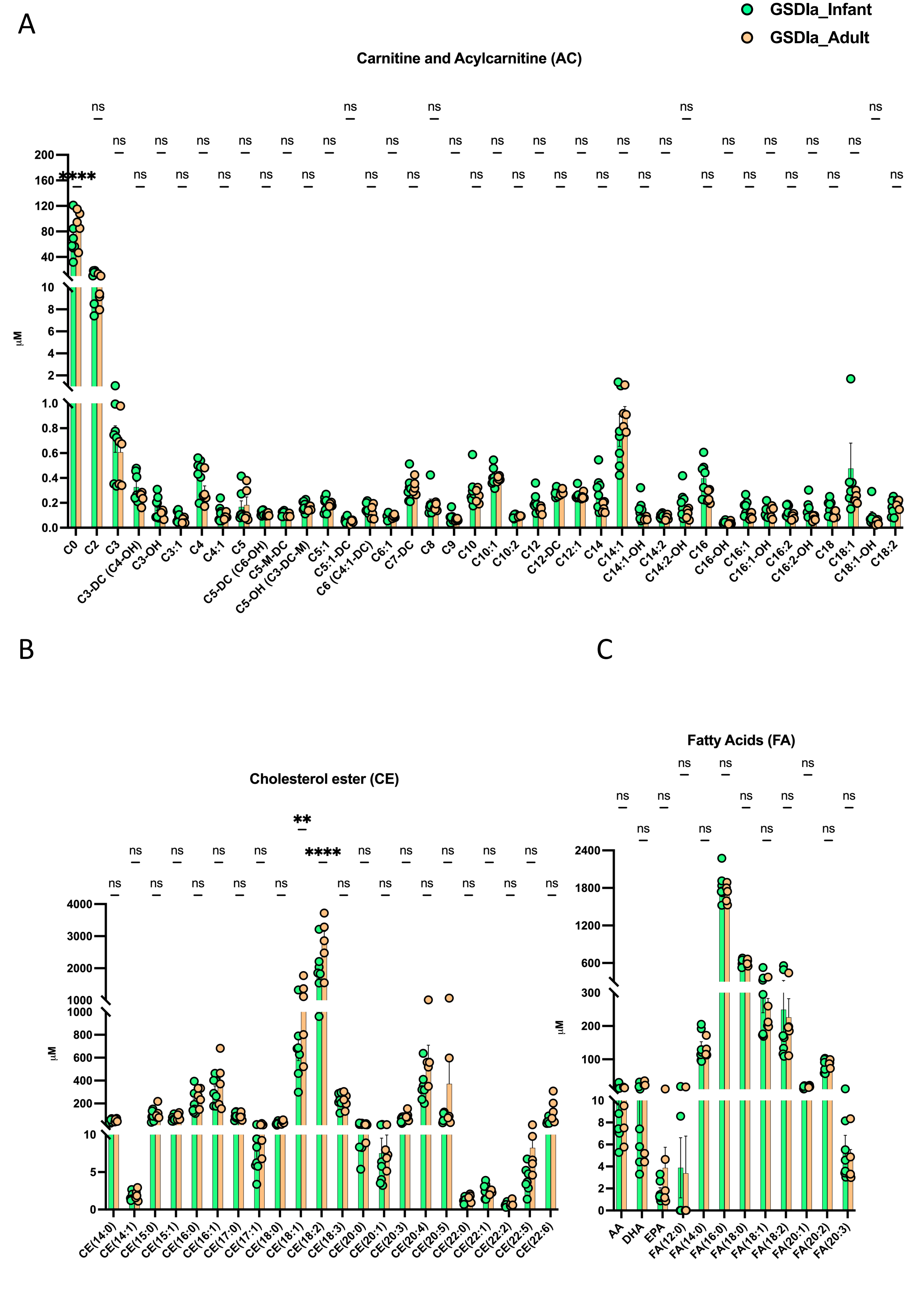

Supplementary Figure S8

D

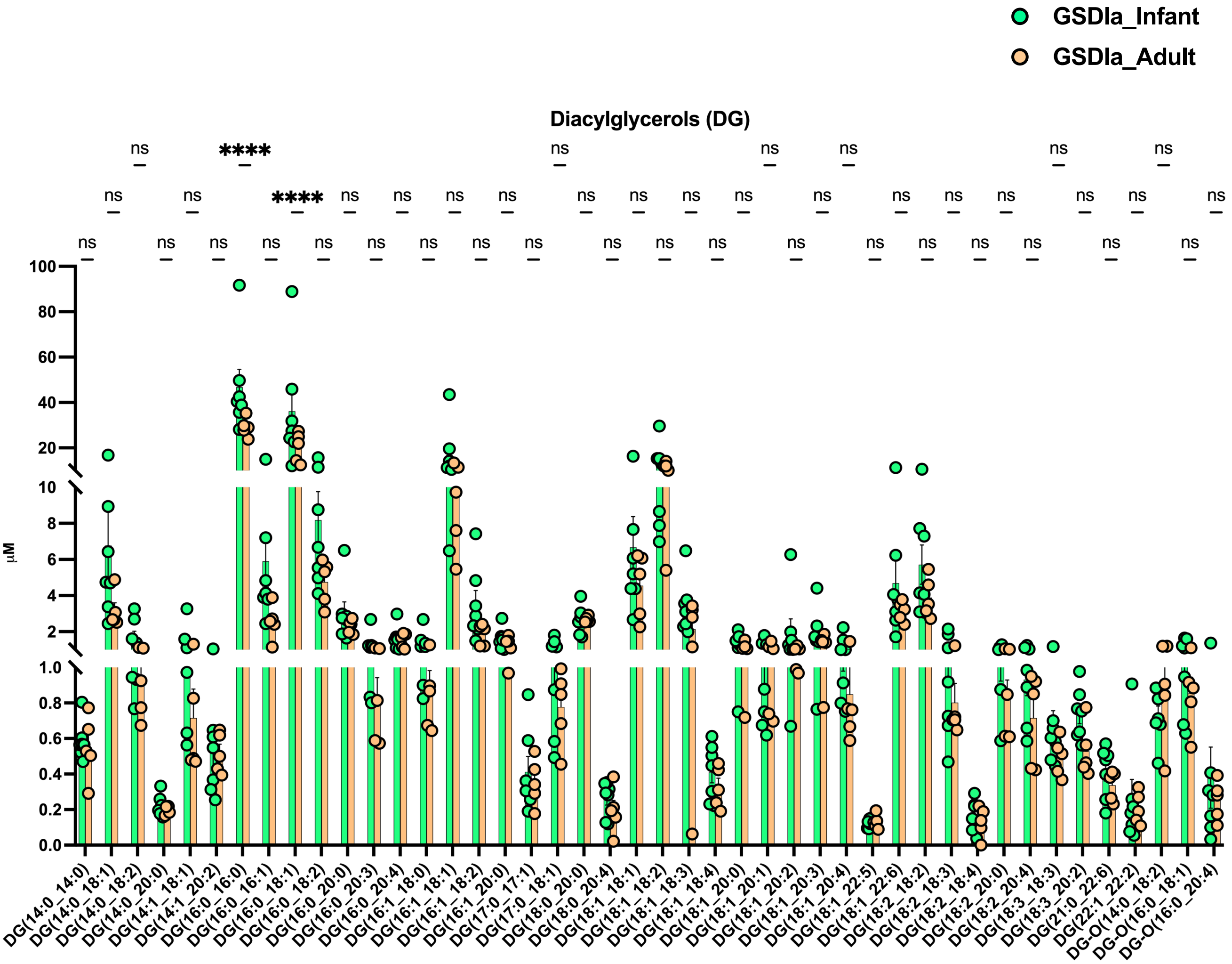

E

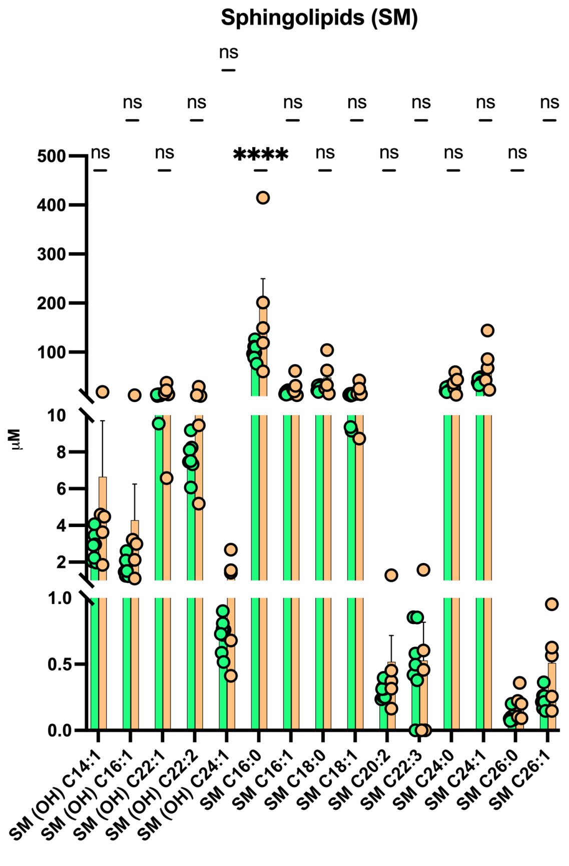

F

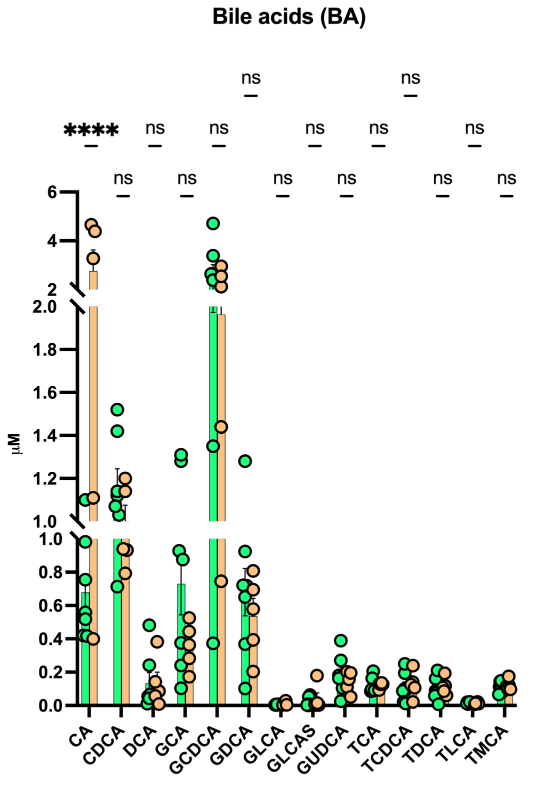

Supplementary Figure S8

G

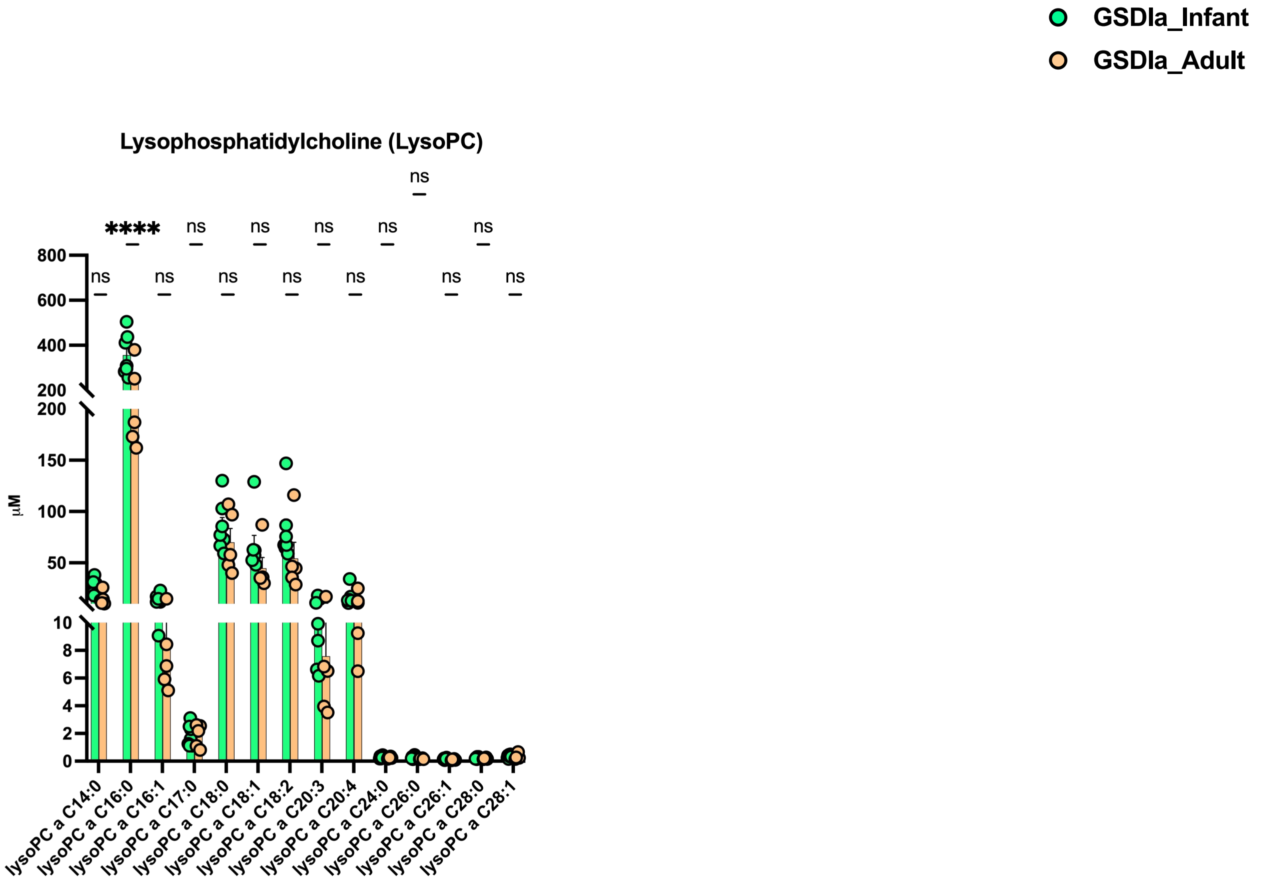

H

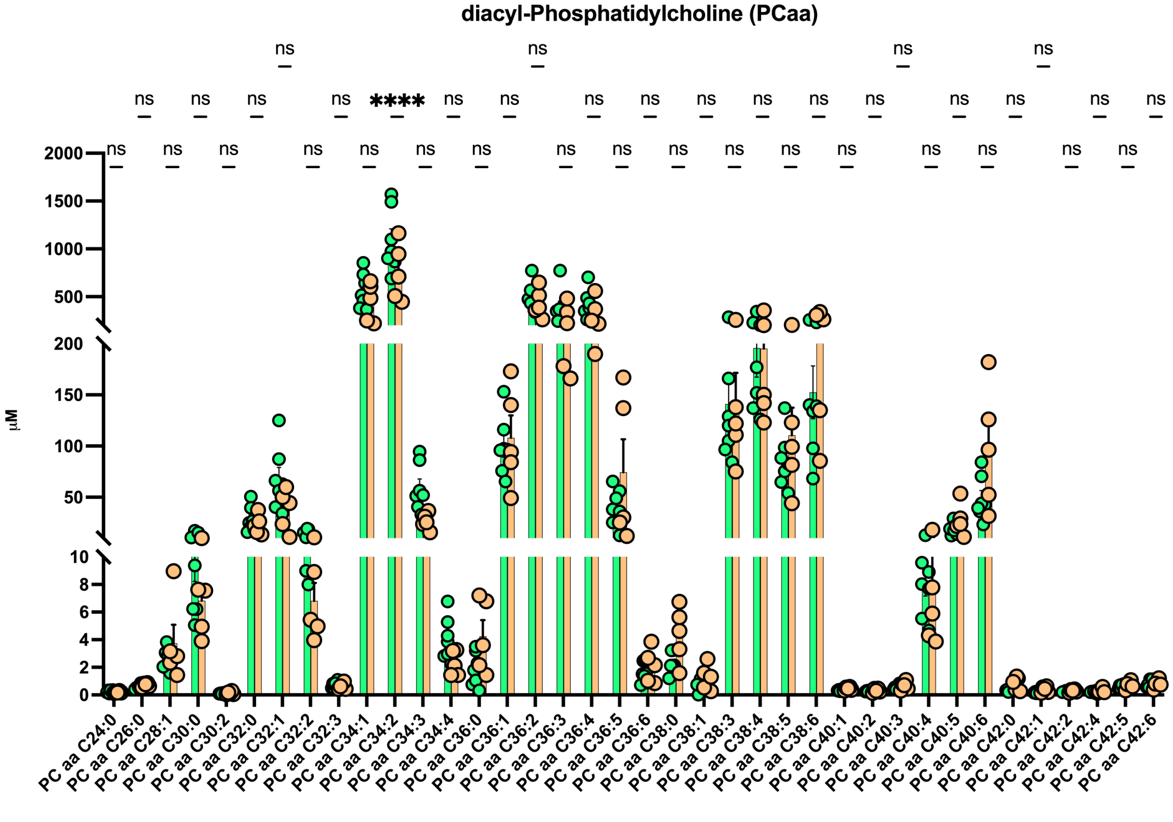

Supplementary Figure S8

I

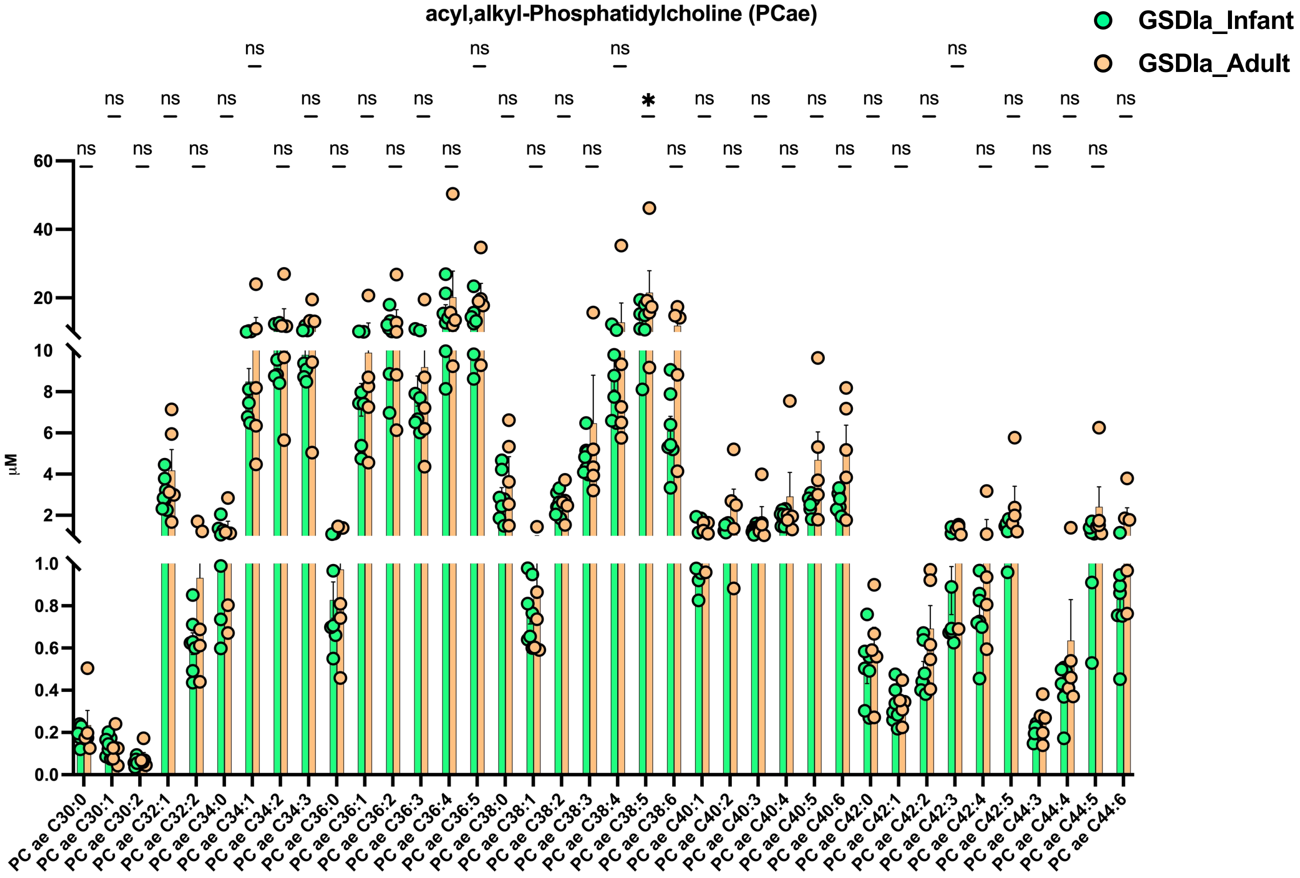

J

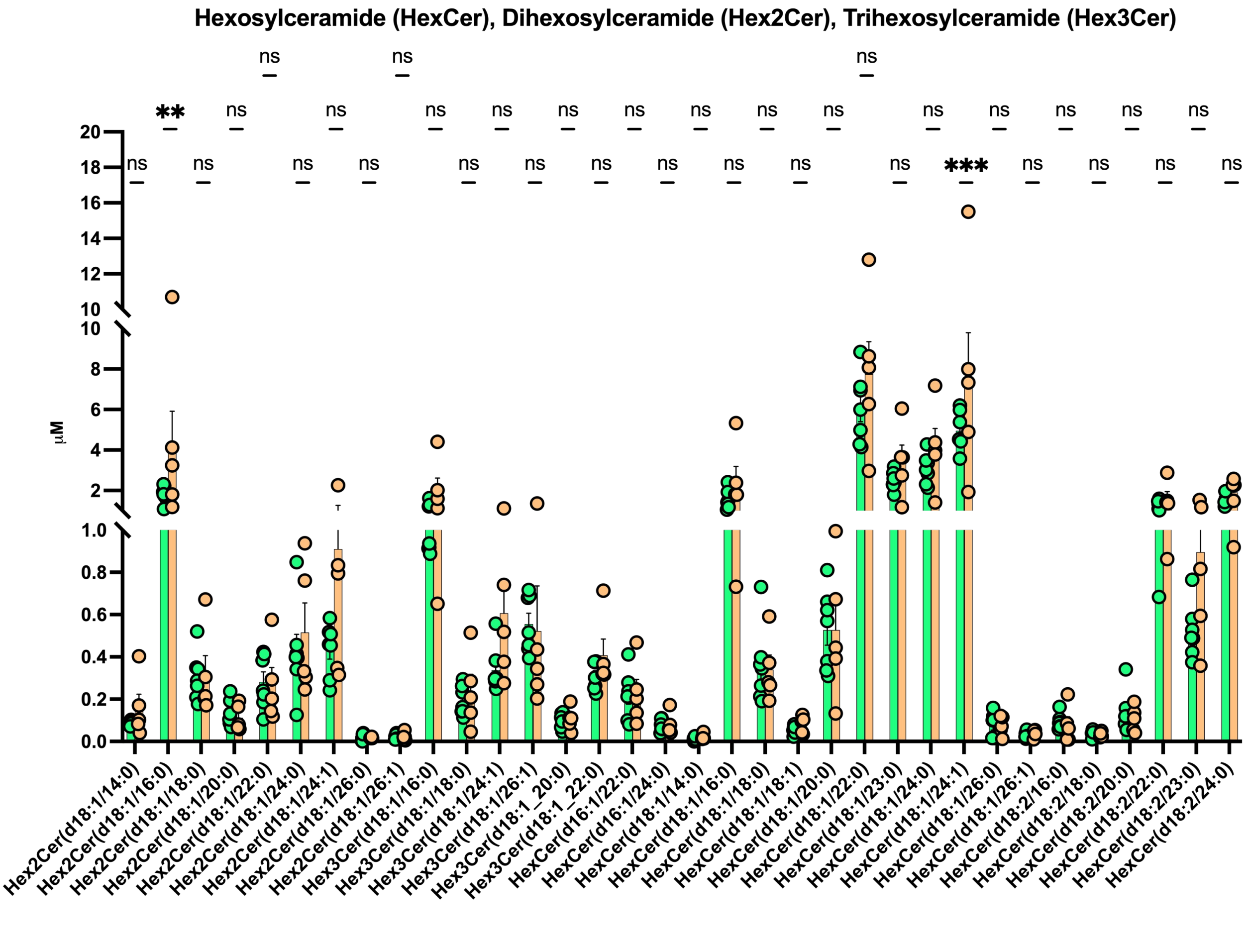

Supplementary Figure S8

K

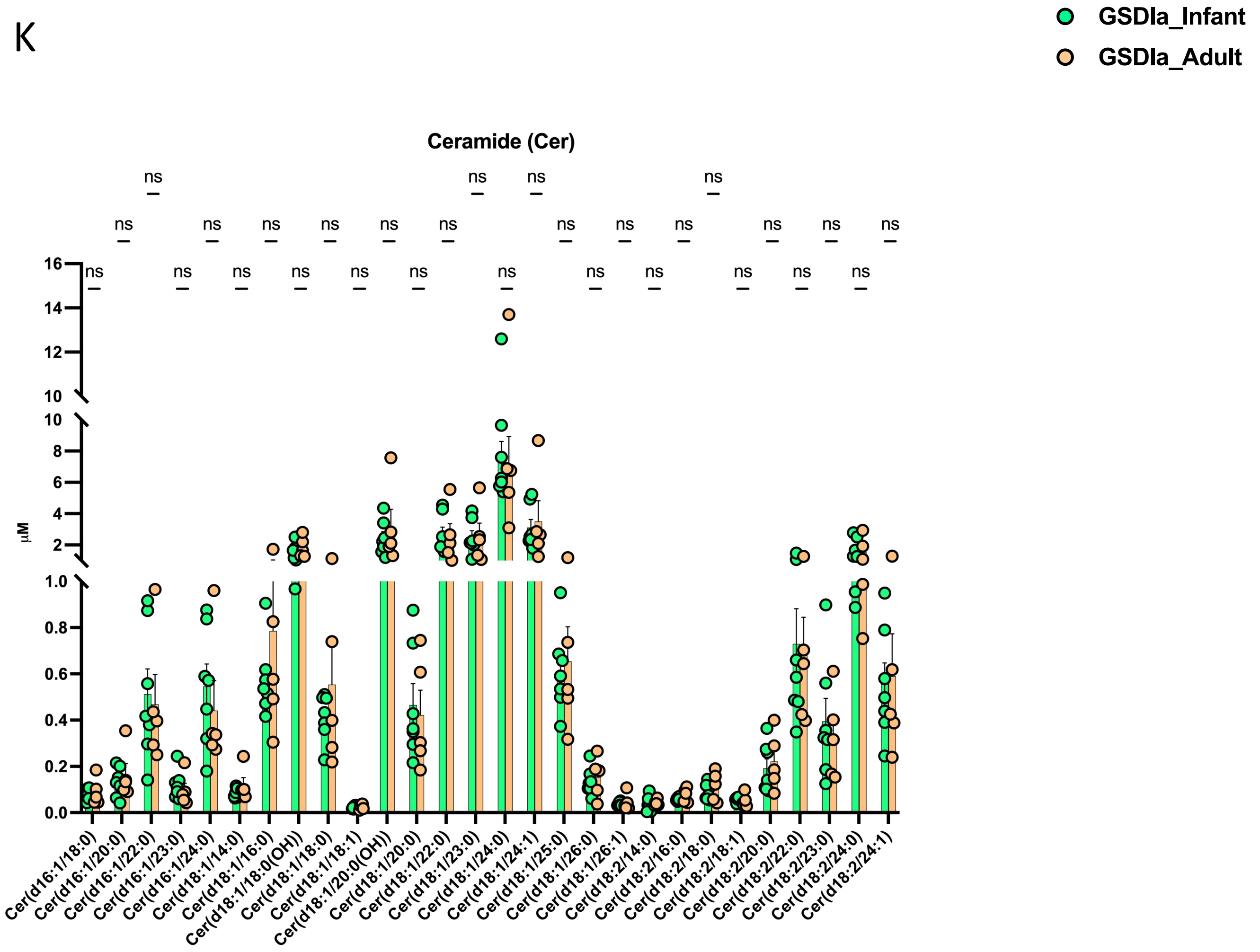

L

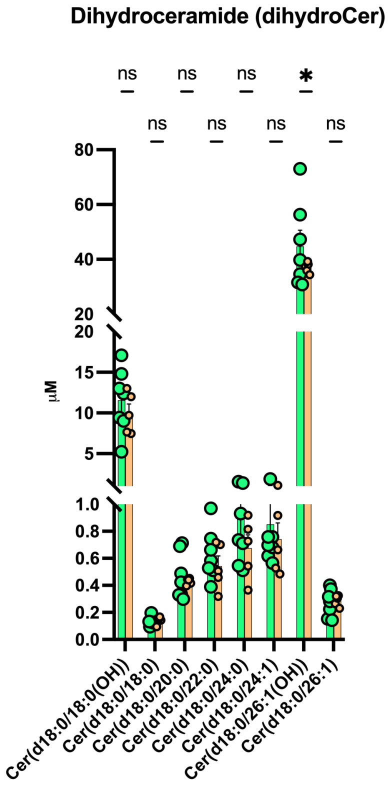

Supplement: Supplemental Figures [file mmc1.pdf]
